# Divergent Synthesis of 1‑Aminonaphthalene Derivatives via the Diels–Alder Reaction of 3‑Aminoarynes

**DOI:** 10.1021/acs.joc.5c03079

**Published:** 2026-02-27

**Authors:** Tomoka Okada, Mayu Kawada, Suguru Yoshida

**Affiliations:** Department of Biological Science and Technology, Faculty of Advanced Engineering, Tokyo University of Science, 6-3-1 Niijuku, Katsushika-ku, Tokyo, 125-8585, Japan

## Abstract

A divergent synthesis of 1-aminonaphthalenes by the Diels–Alder reaction of 3-aminoarynes followed by diversification is disclosed. The cycloaddition of 3-aminoarynes with 2-substituted furans exhibits unusual proximal selectivity. Owing to the significant versatility of the resulting tricyclic ethers, a wide variety of highly functionalized 1-aminonaphthalenes can be prepared, thereby enabling rapid development of fluorescent dyes.

## Graphical abstract

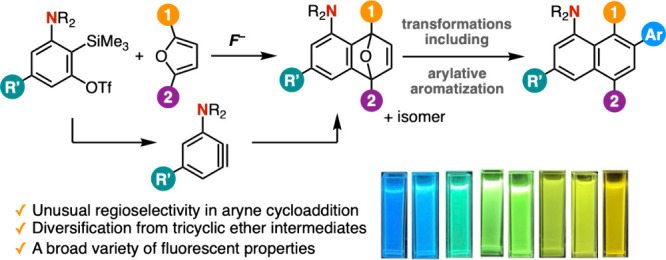

## Introduction

A wide range of 1-aminonaphthalenes play crucial roles across diverse fields, including organic chemistry, pharmaceutical sciences, materials science, and chemical biology (Figure 1A).^1,2^ Among them, the 1-(dimethylamino)­naphthalenesulfonyl (dansyl) group is widely used as a fluorescent tag for biomolecular labeling. Despite their broad utility, the synthesis of structurally diverse 1-aminonaphthalenes remains challenging due to difficulties associated with site-selective functionalization.^2^ For example, the synthesis of methyl 5-morpholinonaphthalene-1-carboxylate from naphthalene-1-carboxylic acid requires multiple steps, including regioselective bromination, esterification, and palladium-catalyzed amination (Figure 1B).^3,4^ However, access to analogs with different substitution patterns is often limited by the poor regioselectivity of electrophilic halogenation. Herein, we report an efficient strategy for the synthesis of a broad array of multisubstituted 1-aminonaphthalenes via the Diels–Alder reaction of 3-aminoaryne intermediates, followed by aromatization and subsequent diversification.

**Figure 1:**
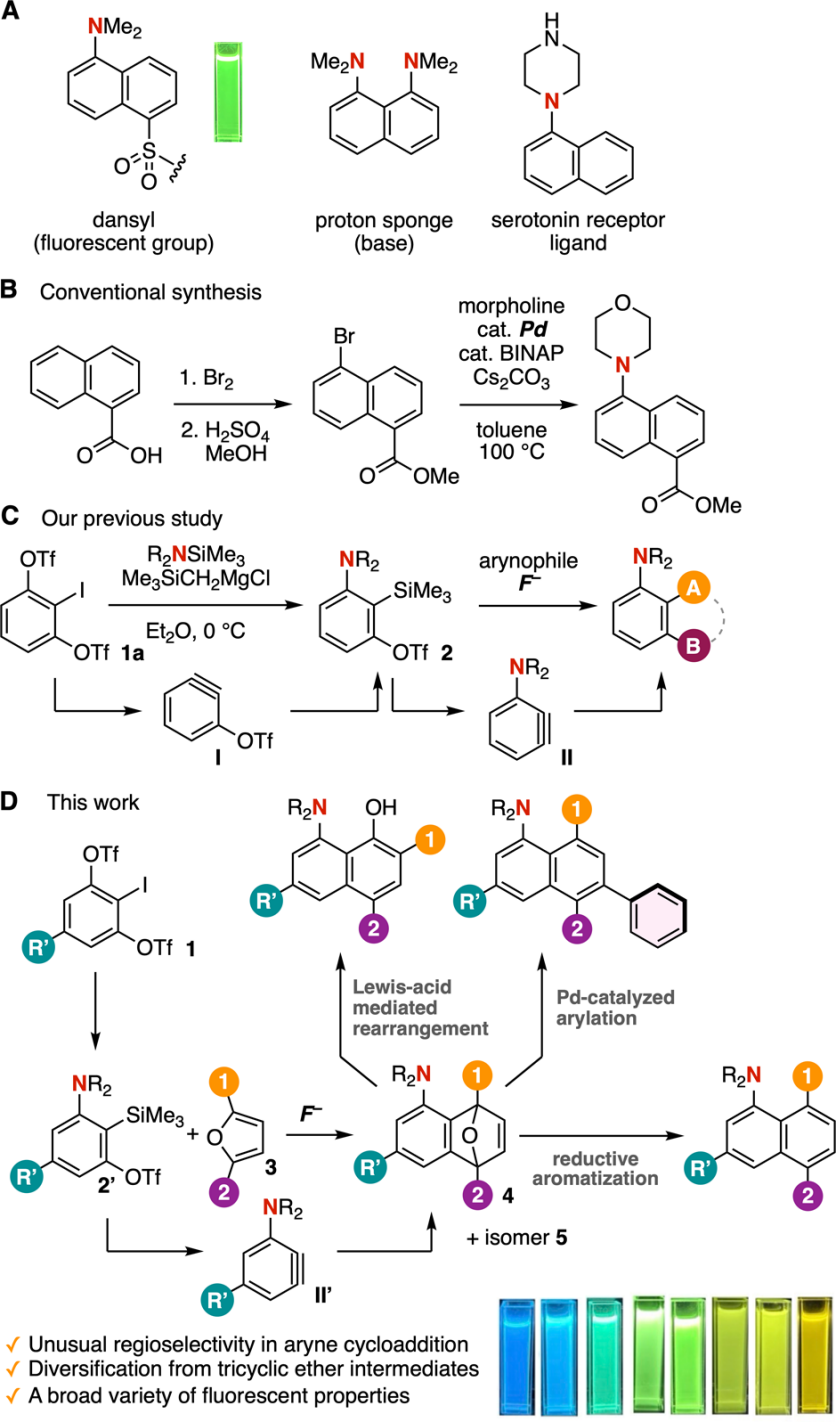
(A) Significant 1-aminonaphthalenes. (B) Conventional synthetic approach. (C) Our previous study. (D) This work.

Transformations of 3-aminoarynes have enabled the efficient synthesis of a wide range of functionalized anilines, as demonstrated in our recent studies (Figure 1C).^5,6^ In 2014, we successfully generated 3-(triflyloxy)­benzyne (**I**) from 1,3-bis­(triflyloxy)-2-iodobenzene (**1a**) with (trimethylsilyl)­methylmagnesium chloride as an activator.^7^ We subsequently developed an efficient method for synthesizing 3-amino-2-silylaryl triflates via regioselective aminosilylation of 3-(triflyloxy)­arynes by treating **1a** with the silylmethyl Grignard reagent in the presence of aminosilanes.^5,8^ Building on these achievements, we envisioned that structurally diverse 1-aminonaphthalenes could be accessed through the cycloaddition of 3-aminoarynes with furans, followed by aromatization-driven diversifications. A key feature of this transformation is the divergent synthetic utility of cycloadducts **4** and **5**, which enable the construction of multisubstituted frameworks.^9^ In this manuscript, we present both experimental and theoretical investigations into the unusual regioselectivity observed in the cycloaddition of 3-aminoarynes with furans. Furthermore, we demonstrate broad derivatizations of the resulting tricyclic ethers and provide a comprehensive analysis of their structure–fluorescence relationships.

## Results and Discussion

Various symmetric furans **3a**–**3c** efficiently reacted with 3-morpholinobenzyne (**IIa**), which was generated from 3-morpholino-2-(trimethylsilyl)­phenyl triflate (**2a**) triggered by fluoride ion (Figure 2A). For example, treatment of *o*-silylaryl triflate **2a** with 2,5-diphenylfuran (**3a**) in the presence of potassium fluoride and 18-crown-6 in tetrahydrofuran (THF) at room temperature provided tricyclic ether **4a** quantitatively without the cleavage of C–O bonds. When unsubstituted furan (**3b**) was used, cycloadduct **4b** was prepared in moderate yield. We also succeeded in the synthesis of anthracene derivative **4c** quantitatively by the Diels–Alder reaction of 3-morpholinobenzyne (**IIa**) with 1,3-diphenylisobenzofuran (**3c**). In addition, tetraphenyl-substituted 1-morpholinonaphthalene **7a** was synthesized from *o*-silylaryl triflate **2a** and 2,3,4,5-tetraphenyl-2,4-cyclopentadien-1-one (**6**) in moderate yield through cycloaddition followed by aromatization via carbon monoxide extrusion (Figure 2B).^10^

**Figure 2:**
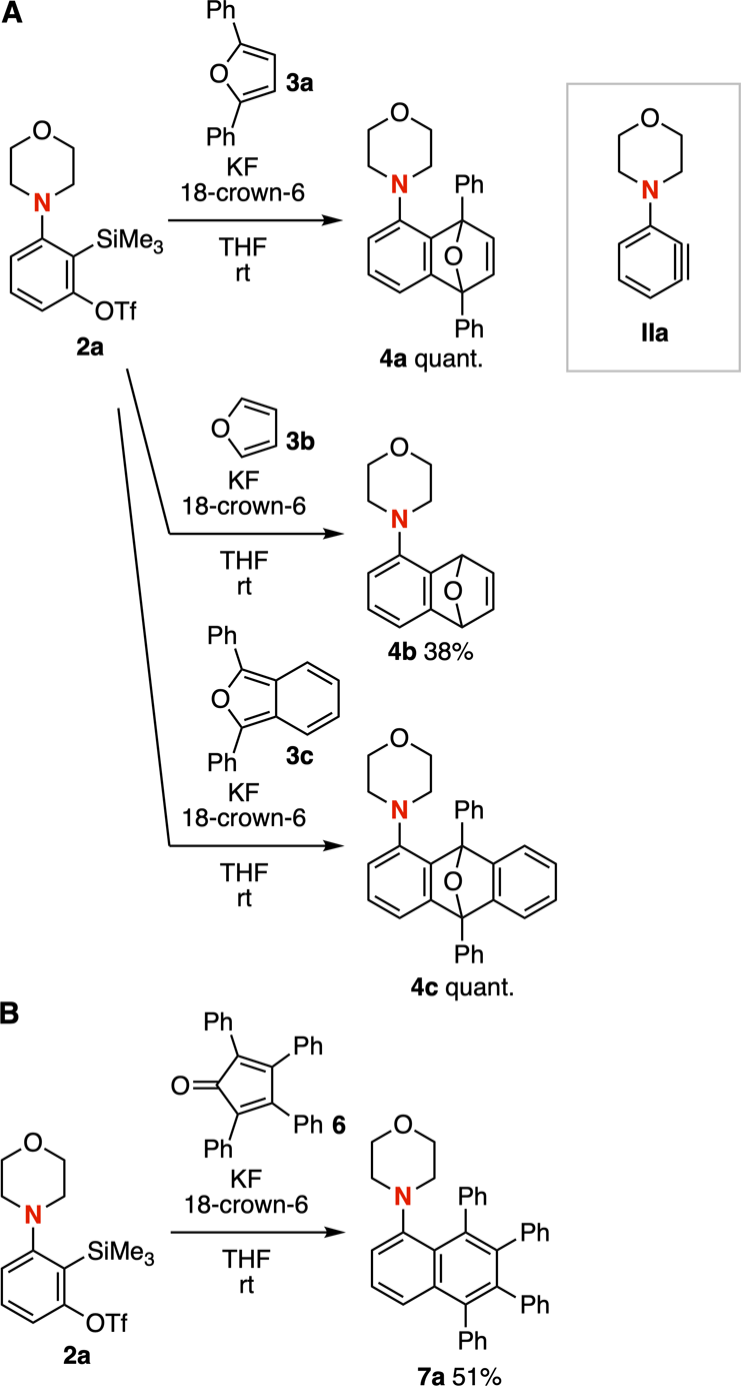
(A) Reactions of **2a** with furans **3a**–**3c**. (B) Reaction of **2a** with diene **6**. See the Supporting Information for details.

Unusual regioselectivities were observed in the Diels–Alder reactions between 3-morpholinobenzyne (**IIa**) and 2-substituted furans **3d**–**3i** (Figure 3). When we treated a mixture of 3-morpholinobenzyne precursor **2a** and 2-(methoxycarbonyl)­furan (**3d**) with a fluoride ion, a regioisomeric mixture of cycloadducts **4d** and **5d** was obtained in moderate combined yield, with the proximal isomer predominating despite steric hindrance from the substituent. The structure of **4d** was unambiguously confirmed by ^1^H and ^13^C NMR spectra and X-ray crystallographic analysis. A similar proximal selectivity was observed with furan **3e** having an electron-donating *n*-butyl group at the 2-position. Likewise, 2-cyanofuran smoothly reacted with 3-morpholinobenzyne to afford proximal cycloadduct **4f** as the major isomer, accompanied by a minor amount of distal isomer **5f**. The cycloaddition and subsequent regioselective ring-opening with aromatization proceeded efficiently to furnish naphthol **7b** in good yield, with no regioisomer detected, which is in good accordance with a previous report involving 3-methoxybenzyne.^11^ In contrast, the regioselectivity was markedly diminished when 2,3-dimethylfuran (**3h**) or 2-bromo-5-(methoxycarbonyl)­furan (**3i**) was employed, providing nearly 1:1 mixtures of regioisomers **4h**/**5h** and **4i**/**5i**, respectively.

**Figure 3:**
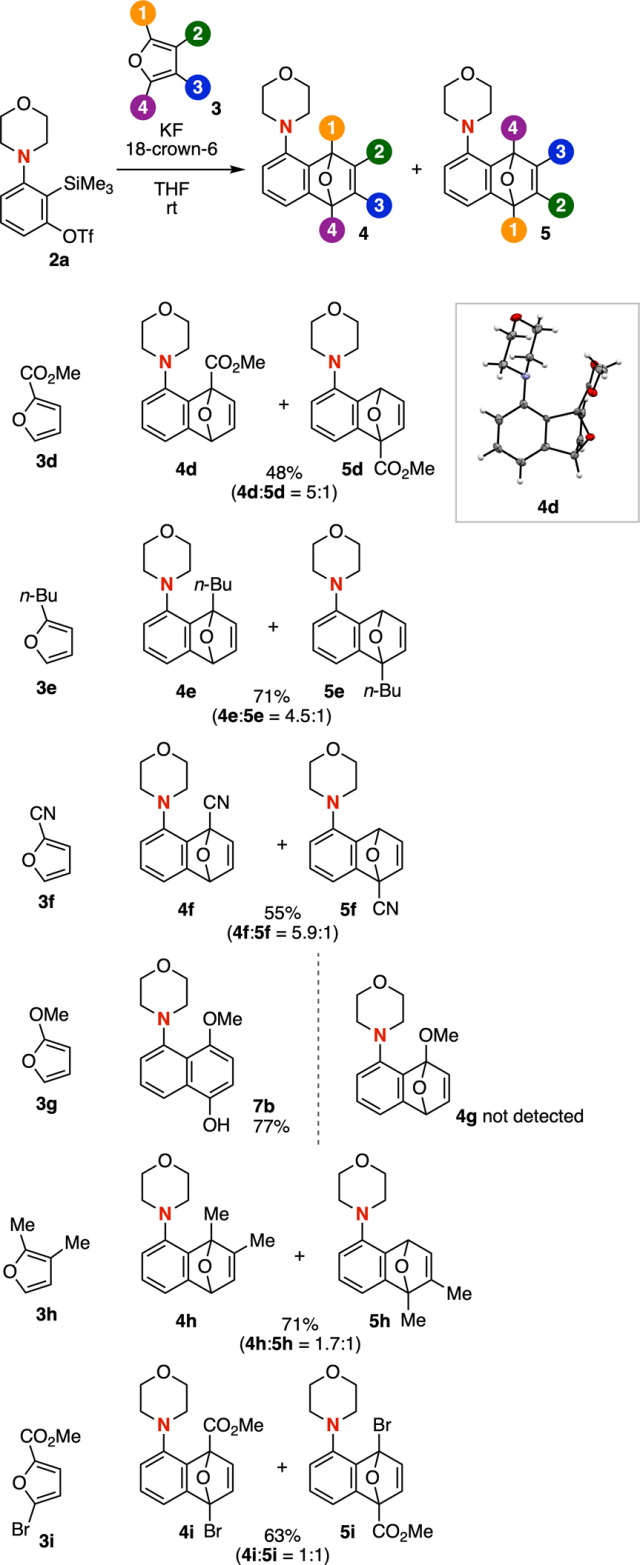
Cycloaddition of **2a** with various unsymmetric furans.

A broad range of 3-aminoaryne precursors successfully participated in the synthesis of 1-aminonaphthalene derivatives (Figure 4). Similar to our previous report on the preparation of **2a**, **2b**, and **2d**,^5a^ the synthesis of 3-aminoaryne precursors **2c** and **2e** was achieved from 1,3-bis­(triflyloxy)-2-iodobenzenes and the corresponding *N*-silylamines through generation of 3-(triflyloxy)­arynes followed by regioselective aminosilylation. In the cycloaddition of 3-aminoarynes with furans **3d** and **3e** bearing an electron-deficient ester moiety and an electron-donating *n*-butyl group, similar selectivity was observed, affording the proximal isomers as the major products (Figure 4A). When using 3-(dimethylamino)­benzyne precursor **2b**, we synthesized 1-aminonaphthalene derivatives **4j** and **4k** as single isomers (Figure 4B). Formation of proximal isomers **4l** and **4m** was also favored in the Diels–Alder reactions of *o*-silylaryl triflate **2c** with furans **3d** and **3e**. The proximal selectivities were slightly reduced when using 5-methyl- and 5-bromo-3-morpholinobenzyne precursors **2d** and **2e**.

**Figure 4:**
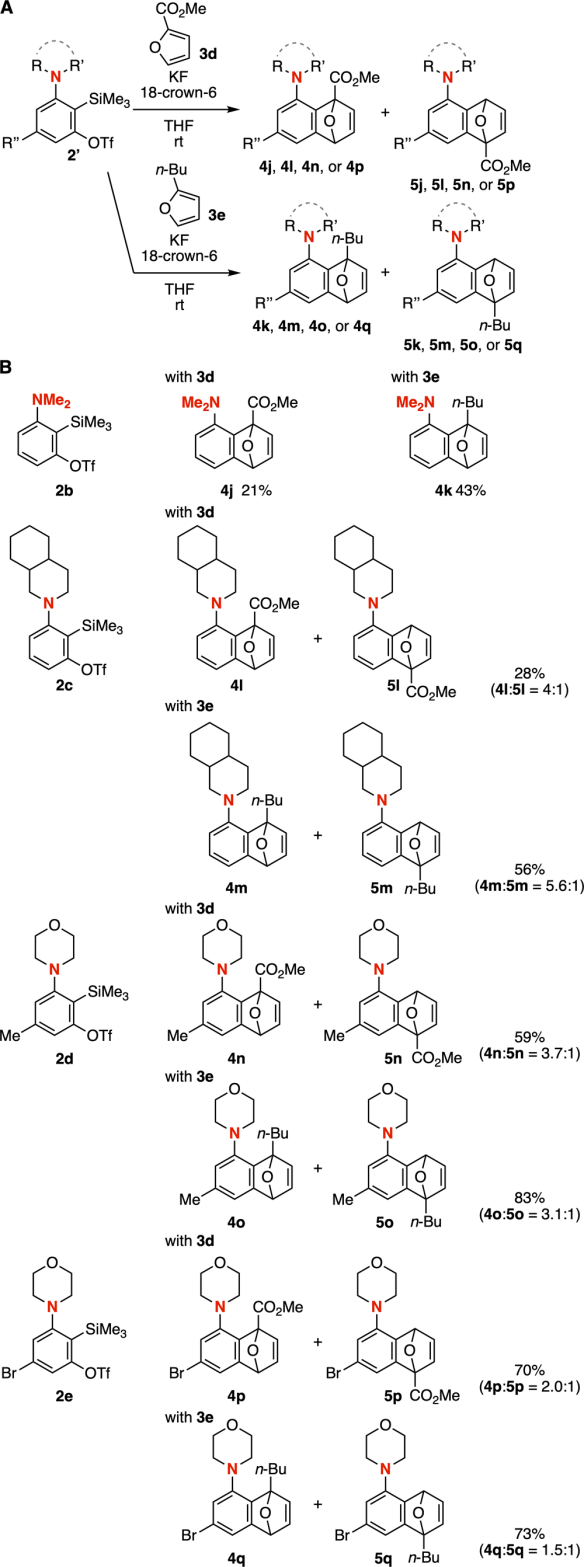
Reactions of 3-aminoaryne precursors with furans **3d** and **3e**. (A) General scheme. (B) Products. See the Supporting Information for details.

To gain insight into the proximal selectivity, we next focused on the Diels–Alder reaction of 3-methoxybenzyne with 2-substituted furans, in which the methoxy group is well-known as an electron-withdrawing substituent in aryne reactions due to the inductive effect of oxygen (Figure 5).^12,13^ In a study by Giles, Sargent et al., reported in 1991, cycloadducts **9a** and **10a** were obtained in an approximately 1:1 ratio from the Diels–Alder reaction of 2-(methoxycarbonyl)­furan (**3d**) with 3-methoxybenzyne (**III**) generated from anthranilic acid **8** through diazotization (Figure 5A).^14^ When we performed the Diels–Alder reaction of 3-methoxy-2-(trimethylsilyl)­phenyl triflate (**11**) as a 3-methoxybenzyne precursor with furans **3d** and **3e**, cycloadducts **9a**/**10a** and **9b**/**10b** were obtained in high yields, with the proximal selectivities decreased compared to that observed for 3-aminoarynes (Figure 5B). The unusual proximal selectivity was also observed in the Diels–Alder reaction of 6-(1-adamantyl)-3-silyloxybenzyne **IV** with 2-(1-adamantyl)­furan (**3j**) as reported by Yamaguchi and co-workers, in which the selectivity was attributed to London dispersion interaction (Figure 5C).^15^ Owing to the greater distortion of 3-methoxybenzyne relative to 3-morpholinobenzyne, the unusual proximal selectivity observed for 3-aminoarynes might be attributed to London dispersion interactions between the amino groups and the 2-substituents of furans.^16^

**Figure 5:**
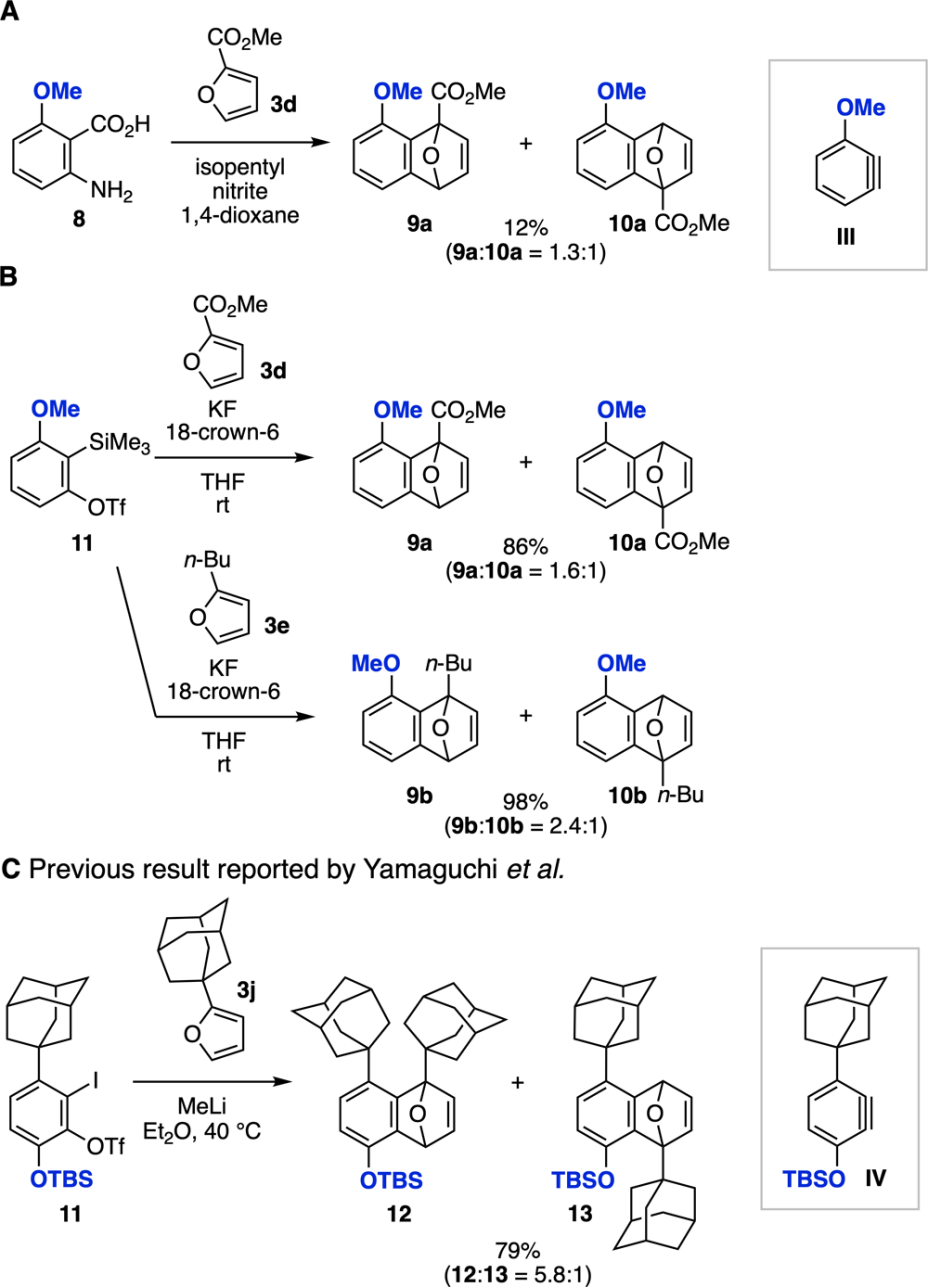
(A) Previous report by Giles, Sargent et al. (B) Reactions of **11** with furans **3d** and **3e**. (C) Similar selectivity reported by Yamaguchi et al.

The Diels–Alder reactions of 3-aminoarynes with 2-substituted furans were investigated by DFT calculations (B3LYP-D3/6-311+G­(d,p)) (Figure 6). The calculations revealed that the activation energies for forming proximal cycloadducts **4d** and **4r** were slightly lower than those for distal cycloadducts **5d** and **5r**. In addition, the HOMOs of furans **3d** and **3k** showed no significant deviation even in the presence of electron-withdrawing or -donating substituents. According to previous studies on the Diels–Alder reaction between adamantyl-substituted aryne **IV** and 2-(1-adamantyl)­furan (**3j**),^15^ the unusual proximal selectivity observed for 3-aminoarynes with 2-substituted furans can be attributed to London dispersion interactions involving the substituents.^16^

**Figure 6:**
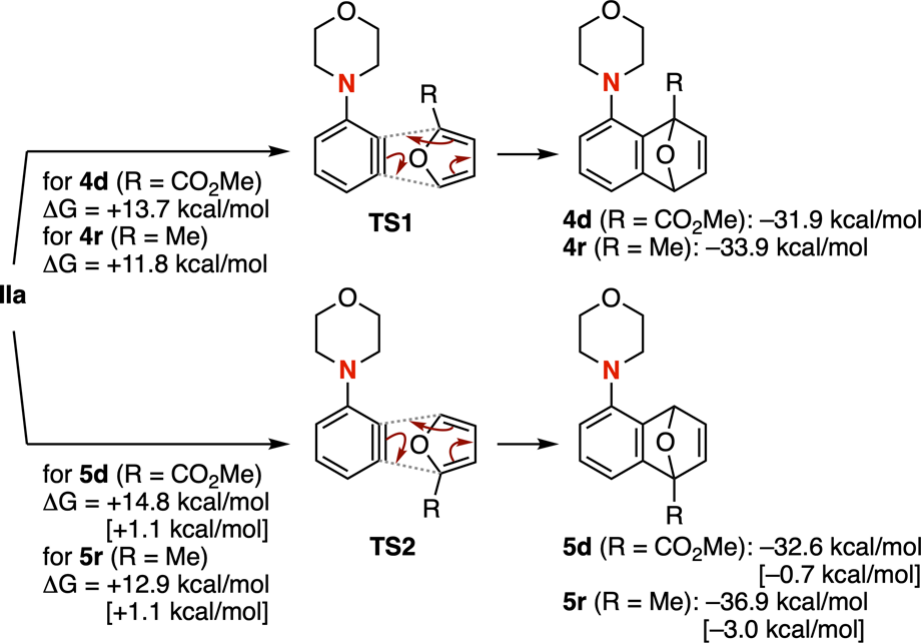
Calculated plausible reaction pathways for the synthesis of tricyclic ethers **4d**/**5d** and **4r**/**5r** by a DFT method (B3LYP-D3/6-311G­(d,p)). Respective energy differences were shown in the figures. See the Supporting Information for details.

The synthetic versatility of tricyclic ethers **4** and **5** allowed for the divergent synthesis of a wide variety of multisubstituted 1-aminonaphthalenes (Figure 7). For instance, reductive aromatization of tricyclic ether **4d** took place smoothly in the presence of sodium iodide and chlorotrimethylsilane to afford 8-(methoxycarbonyl)-1-morpholinonaphthalene (**7c**) in high yield (Figure 7A).^9a^ When tricyclic ether **4d** was treated with boron trifluoride, naphthol **7d** was obtained through aromatization accompanied by rearrangement of the ester moiety from the 8- to the 7-position.^9d^ In addition, the use of 1,1,1,3,3,3-hexafluoro-2-propanol (HFIP) led to a slight improvement in the yield of **7d**. Furthermore, palladium-catalyzed arylation of tricyclic ether **4d** proceeded efficiently to provide 1-morpholino-8-(methoxycarbonyl)-6-phenylnaphthalene (**7e**) by aromatization-driven arylation.^9b^ These results highlight the advantages of this strategy, in which aryne chemistry is combined with subsequent diversification, thereby significantly expanding the scope of accessible multisubstituted 1-aminonaphthalenes.

**Figure 7:**
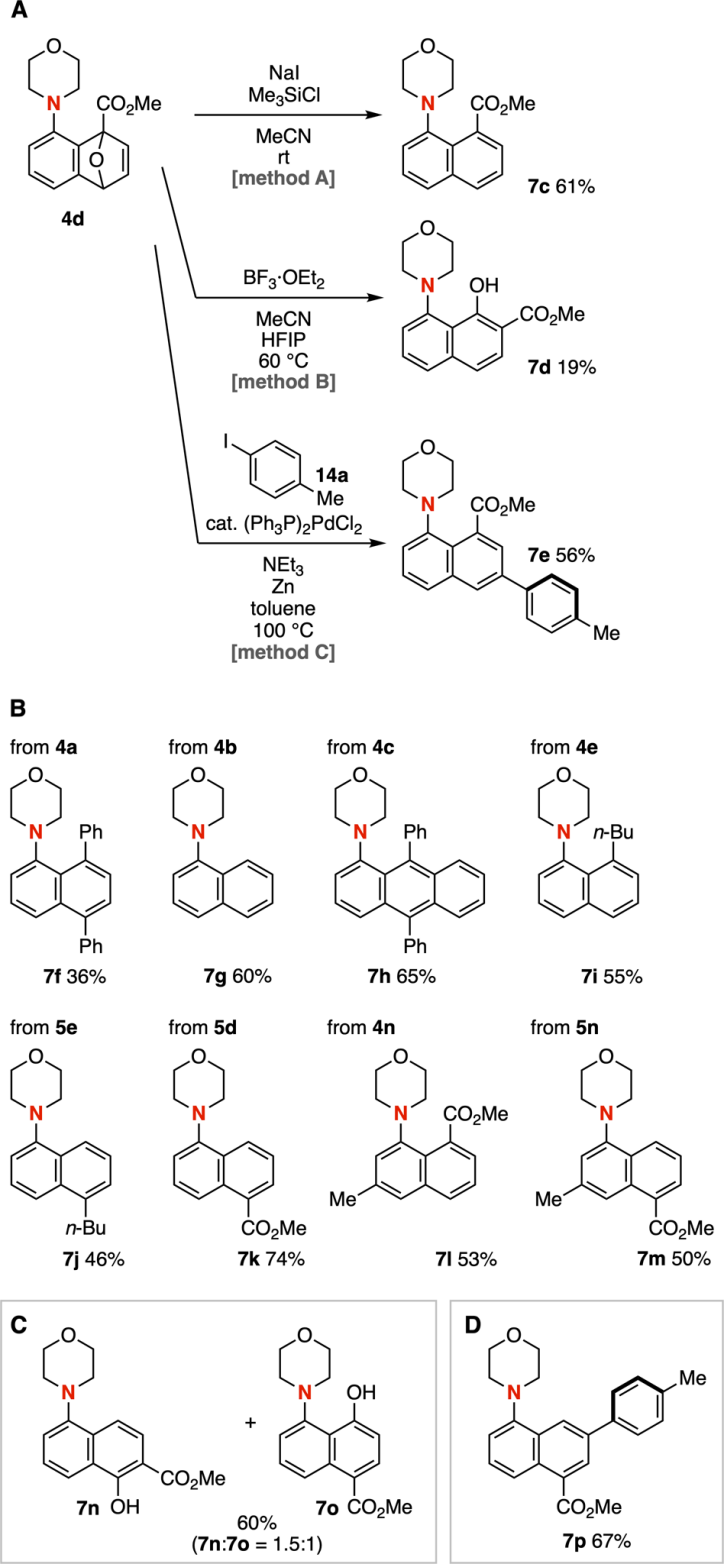
(A) Aromatization-driven transformations of **4d**. (B) Products prepared by method A. (C) Products prepared by method B. (D) Product prepared by method C.

We succeeded in synthesizing 1-morpholinonaphthalenes bearing a wide variety of substituent patterns from the common starting material **2a** (Figure 7B–7D). Reductive aromatization using sodium iodide and chlorotrimethylsilane enabled the preparation of mono-, di-, and trisubstituted naphthalenes and anthracenes **7f**–**7m** in moderate to good yields (Figure 7B). Treatment of tricyclic ether **5d** with boron trifluoride furnished naphthols **7n** and **7o** in high yield, in which rearrangement product **7n** was obtained as the major product (Figure 7C). Moreover, 7-arylation via aromatization was achieved from tricyclic ether **5d** to furnish trisubstituted naphthalene **7p** in good yield (Figure 7D). Therefore, diverse 1-amino-substituted naphthalenes with a broad range of substituent patterns can be accessed from 3-aminoaryne precursors through Diels–Alder reactions with furans followed by diverse aromatization-driven transformations.

The fluorescence properties of the 1-aminonaphthalene derivatives spanned a broad spectral range from blue to yellow (Figure 8). The 1-morpholino substituent in **7g** induced only weak blue emission. Incorporation of a methoxycarbonyl group resulted in a significant red-shift in the emission spectra along with improved quantum yields. For example, light blue fluorescence was observed for naphthalenes **7n** or **7d**, which bear an ester moiety at the 6- or 7-position together with a 5- or 8-hydroxy group, respectively. Introducing an ester moiety at the 5-position produced blue-green or light-green fluorescence in the emission spectra of **7o** and **7k**/**7p**, in which the presence of an 8-hydroxy group led to shorter emission wavelength. It is worth noting that 8-(methoxycarbonyl)­naphthalenes **7c** and **7e** exhibited yellow emission, which can be attributed to a twisted intramolecular charge transfer (TICT) process.^17^ Furthermore, relative to the blue fluorescence of 9,10-diphenylanthracene, 1-morpholino-9,10-diphenylanthracene (**7h**) exhibited yellow emission, with the pronounced red-shift originating from the morpholino group at the 1-position compared to the parent blue-fluorescent 9,10-anthracene.^18^ Notably, versatile fluorescent dyes can be developed from the common 3-morpholinobenzyne precursor **2a** with readily available starting materials including furans and aryl iodides. Since a wide range of 3-aminoaryne precursors **2** can be synthesized from 1,3-bis­(triflyloxy)-2-iodobenzenes,^5^ this approach will enable the practical and modular development of highly functionalized aminonaphthalene derivatives.

**Figure 8:**
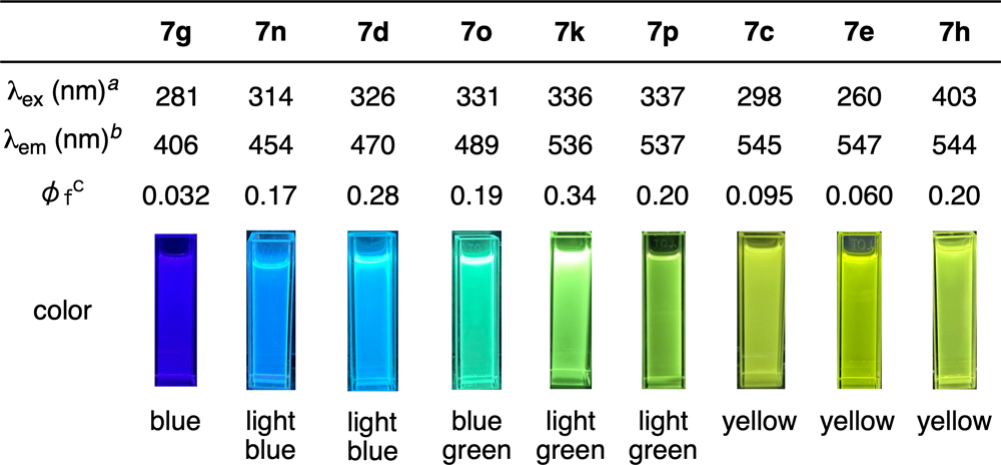
Fluorescent properties of aminonaphthalene derivatives. See the Supporting Information for details. ^*a*^Wavelength of maximum absorption. ^*b*^Wavelength of maximum fluorescent intensity. ^*c*^Quantum yields were determined with 9,10-diphenylanthracene as a standard.

The arylative aromatization of tricyclic ether **5d** with aryl iodide **14b** bearing a clickable functional group enabled the synthesis of 7-arylated 1-aminonaphthalene **7q** (Figure 9A). The high reliability of click chemistry, particularly the sulfur­(IV) fluoride exchange (SuFEx) reaction, facilitates efficient coupling with sulfonyl fluorides.^19^ For example, treatment of silyl ether **7q** with a sulfonyl fluoride in the presence of 1,8-diazabicyclo[5.4.0]­undec-7-ene (DBU) provided sulfonic acid ester **15** in high yield. The successful gram-scale synthesis of silyl ether **7q** from aryne precursor **2a** via cycloadducts **4d** and **5d** highlights the robustness and practicality of the synthetic protocol established in this study (Figure 9B and 9C). Since esters can be readily transformed into carboxylic acids and *N*-succinimidyl esters, the functionalization of methoxycarbonyl-substituted 1-aminonaphthalenes is expected to be broadly valuable in pharmaceutical sciences and chemical biology.

**Figure 9:**
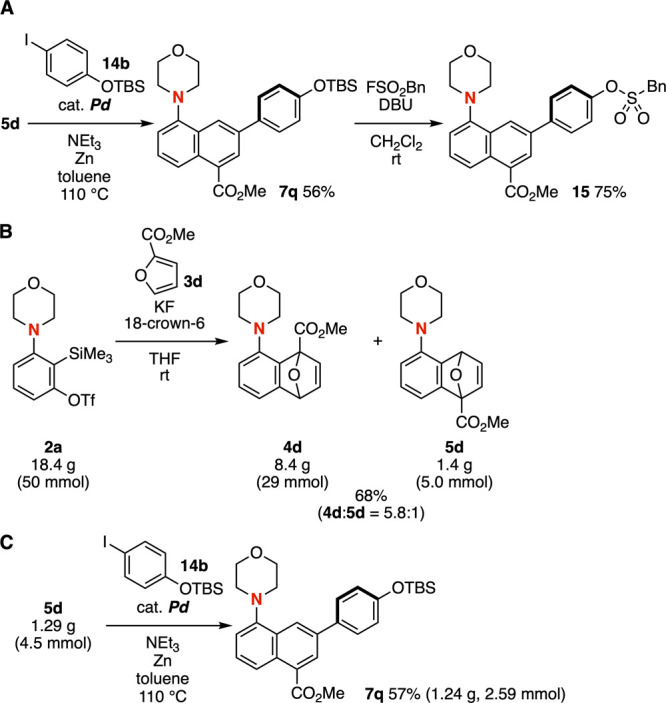
(A) Synthesis of silyl ether **7q** followed by SuFEx reaction. (B) A gram-scale synthesis of **4d** and **5d**. (C) A gram-scale synthesis of **7q**. See the Supporting Information for details.

## Conclusions

In summary, a wide range of 1-aminonaphthalenes were synthesized from 3-aminoaryne precursors and furans through a Diels–Alder reaction followed by subsequent transformations. The cycloaddition between 3-aminoarynes and 2-substituted furans exhibited unique proximal selectivity, which was rationalized by theoretical calculations. Owing to the remarkable synthetic versatility of the resulting tricyclic ethers, diverse and highly functionalized 1-aminonaphthalenes were readily prepared, thereby enabling the rapid development of novel fluorescent dyes. Further studies are underway in our laboratory, including detailed investigations into the unusual proximal selectivity of Diels–Alder reactions of various 3-substituted arynes, structural optimization of these aminonaphthalene derivatives, and applications toward the development of chemical probes.

## Supplementary Material



## Data Availability

The data underlying this study are available in the published article and its Supporting Information.

## Notes

Experimental procedures, characterization for new compounds including NMR spectra (PDF)
