# Supplementary material for: Divergent Synthesis of 1‑Aminonaphthalene Derivatives via the Diels–Alder Reaction of 3‑Aminoarynes
Source: J Org Chem. 2026 Feb 27;91(10):3744–50. doi: 10.1021/acs.joc.5c03079 (PMC12993852; doi:10.1021/acs.joc.5c03079)

Supporting Information

Divergent Synthesis of 1-Aminonaphthalene Derivatives via the Diels–Alder Reaction of 3-Aminoarynes

Tomoka Okada, Mayu Kawada, and Suguru Yoshida*

Department of Biological Science and Technology, Faculty of Advanced Engineering, Tokyo University of Science, 6-3-1 Niijuku, Katsushika-ku Tokyo 125-8585, Japan

Contents

General Information **S1**
Experimental Procedures **S2**
Absorption and Fluorescent Properties and Spectra **S5**
Computational Methods **S6**
Characterization Data of New Compounds **S15**
References for Supporting Information **S30**
^{1}H and ^{13}C NMR Spectra of Compounds **S31**

General Information

All reactions were performed with dry glassware under atmosphere of argon, unless otherwise noted. Analytical thin-layer chromatography (TLC) was performed on precoated (0.25 mm) silica-gel plates (Merck Chemicals, Silica Gel 60 F254, Cat. No. 1.05715). Column chromatography was conducted using silica-gel (Kanto Chemical Co., Inc., Silica Gel 60N, spherical neutral, particle size 40–50 μm, Cat. No. 37562-85 or particle size 63–210 μm, Cat. No. 37565-85). Preparative TLC (PTLC) was performed on silica gel (Wako Pure Chemical Industries Ltd., Wakogel B-5F, Cat. No. 230-00043). Melting points (Mp) were measured on an OptiMelt MPA100 (Stanford Research Systems), and are uncorrected. ^{1}H NMR spectra were obtained with a Bruker AVANCE 400 spectrometer at 400 MHz. ^{13}C{^{1}H} NMR spectra were obtained with a Bruker AVANCE 400 spectrometer at 101 MHz. ^{19}F NMR spectra were obtained with a Bruker AVANCE 400 spectrometer at 376 MHz. All NMR measurements were carried out at 25 ºC. $CDCl_3$ (Kanto Chemical Co. Inc., Cat. No. 07663-23) was used as a solvent for obtaining NMR spectra. Chemical shifts (δ) are given in parts per million (ppm) downfield from the solvent peak (δ 7.26 for ^{1}H NMR in $CDCl_3$, δ 77.0 for ^{13}C NMR in $CDCl_3$) as an internal reference with coupling constants (*J*) in hertz (Hz). The abbreviations s, d, t, q, and m signify singlet, doublet, triplet, quartet, and multiplet, respectively. High-resolution mass spectra (HRMS) were measured on a JEOL JMS-T100CS "AccuTOF CS" mass spectrometer under positive electrospray ionization (ESI^+) conditions. The absorbance spectra (UV/Vis) and fluorescence spectra (FL) were measured with a JASCO UV-750 spectrophotometer and a JASCO FP-8250 spectrofluorophotometer, respectively, at 25 °C using a quartz cuvette (10 mm light path). Fluorescence quantum yields were estimated by using 9,10-diphenylanthracene (9,10-DPA) in cyclohexane (ϕ = 0.95).

Unless otherwise noted, materials obtained from commercial suppliers were used without further purification. 3-Morpholino-2-(trimethylsilyl)phenyl trifluoromethanesulfonate (**2a**), 3-(dimethylamino)-2-(trimethylsilyl)phenyl trifluoromethanesulfonate (**2b**), 5-methyl-3-morpholino-2-(trimethylsilyl)phenyl triflate (**2d**), 5-bromo-3-morpholino-2-(trimethylsilyl)phenyl triflate (**2e**),[S1] 3-methoxy-2-(trimethylsilyl)phenyl trifluoromethanesulfonate (**11**),[S2] and 1-dimethyl-*tert*-butylsilyl-4-iodophenol (**14b**)[S3] were prepared according to the reported methods.

Experimental Procedures

*General procedure for the synthesis of o-silylaryl triflates **2***

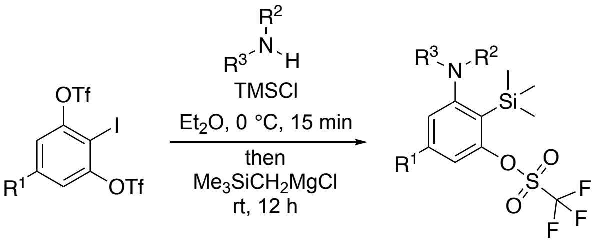

*General procedure for the synthesis of cycloadducts **4** and **5***

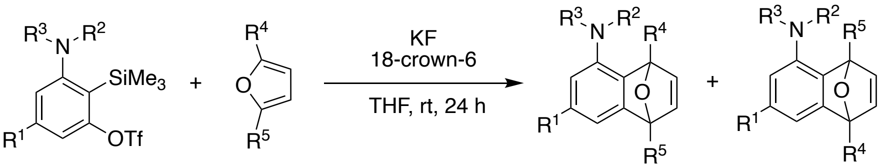

*A typical procedure for the synthesis of o-silylaryl triflates **2***

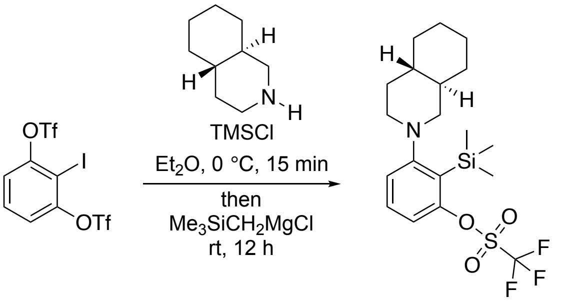

To a solution of 1,3-bis(triflyloxy)-2-iodobenzene (**1a**) (2.50 g, 5.00 mmol, 1.0 equiv) and *trans*-decahydroisoquinoline (2.23 mL, 15.2 mmol, 3.0 equiv) dissolved in Et_2O (25 mL) was added chloro(trimethyl)silane (2.53 mL, 20.0 mmol, 4.0 equiv) at 0 °C. After stirring for 15 min at the same temperature, to the mixture was slowly added (trimethylsilyl)methylmagnesium chloride (0.92 M, Et_2O solution, 6.51 mL, 6.0 mmol, 1.2 equiv) at 0 °C. Then, the resulting mixture was warmed to room temperature. After stirring for 12 h at the same temperature, to the mixture was added water (50 mL). The mixture was extracted with EtOAc (50 mL × 3). The combined organic extract was washed with brine (50 mL) and dried with Na_2SO_4. After filtration, the filtrate was concentrated under reduced pressure. The residue was purified by flash column chromatography (silica-gel 88 g, *n*-hexane/EtOAc = 12/1) to give 3-((4a*S**,8a*R**)-octahydroisoquinolin-2(1*H*)-yl)-2-(trimethylsilyl)phenyl trifluoromethanesulfonate (**2c**) (1.28 g, 2.95 mmol, 59%) as a colorless oil.

*A typical procedure for the synthesis of cycloadducts **4** and **5***

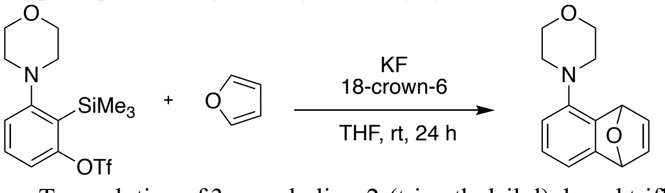

To a solution of 3-morpholino-2-(trimethylsilyl)phenyl trifluoromethanesulfonate (**2a**) (75.6 mg, 0.197 mmol, 1.0 equiv) and furan (69.7 mg, 1.02 mmol, 5.1 equiv) dissolved in THF (1.0 mL) were added potassium fluoride (34.9 mg, 0.600 mmol, 3.0 equiv) and 18-crown-6-ether (159 mg, 0.603 mmol, 3.0 equiv) at room temperature. After stirring for 24 h at the same temperature, to the mixture was added water (3 mL). The mixture was extracted with EtOAc (3 mL × 3). The combined organic extract was washed with brine (3 mL) and dried with Na_2SO_4. After filtration, the filtrate was concentrated under reduced pressure. The residue was purified by preparative TLC (*n*-hexane/EtOAc = 5/1) to give 4-(1,4-dihydro-1,4-epoxynaphthalen-5-yl)morpholine (**4b**) (16.9 mg, 75.0 μmol, 38%) as a colorless solid.

Similarly, cycloadducts **4a**, **4d**–**4q**/**5d**–**5q**, and **7b** were prepared from the corresponding *o*-silylaryl triflates and furans. Cycloadduct **4c** was prepared from **2a** (2.0 equiv) and 9,10-diphenylisobenzofuran (**3c**) (1.0 equiv).

A typical procedure for reductive aromatization [method A]

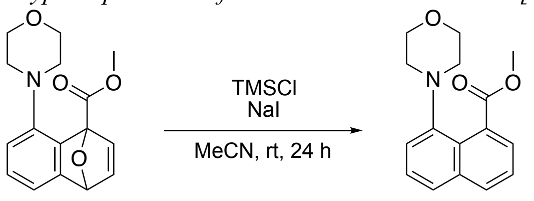

To a solution of methyl 8-morpholino-1,4-epoxynaphthalene-1(4*H*)-carboxylate (**4d**) (29.6 mg, 0.103 mmol, 1.0 equiv) in acetonitrile (3.7 mL) was added sodium iodide (150 mg, 1.00 mmol, 10.0 equiv) at room temperature. To the mixture was added trimethysilyl chloride (126 µL, 1.00 mmol, 10.0 equiv) at 0 °C. The mixture was stirred at room temperature for 24 h. To the mixture was added an aqueous saturated sodium thiosulfate (5 mL). The mixture was extracted with EtOAc (15 mL × 3). The mixture was washed with brine (15 mL) and dried with Na_2SO_4. After filtration, the filtrate was concentrated under reduced pressure. The residue was purified by preparative TLC (*n*-hexane/EtOAc = 5/1) to give methyl 8-morpholino-1-naphthoate (**7c**) (17.0 mg, 63.0 µmol, 61%) as a colorless solid.

A typical procedure for rearrangement with $BF_3{\cdot}OEt_2$ [method B]

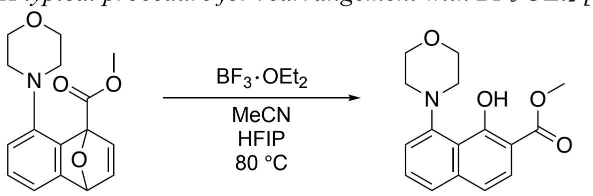

To a mixture of methyl 8-morpholino-1,4-epoxynaphthalene-1(4*H*)-carboxylate (**4d**) (28.4 mg, 0.100 mmol, 1.0 equiv) dissolved in acetonitrile : hexafluoro-2-propanol = 1 : 1 (total 1.0 mL) was added boron trifluoride diethyl ether complex (16.5 µL, 0.131 mmol, 1.3 equiv) at room temperature. After stirring for 24 h at 80 °C, the mixture was cooled to room temperature. To the mixture was added an aqueous saturated sodium hydrogen carbonate solution (3 mL). The mixture was extracted with EtOAc (3 mL × 3). The mixture was washed with brine (3 mL) and dried with Na_2SO_4. After filtration, the filtrate was concentrated under reduced pressure. The residue was purified by preparative TLC (*n*-hexane/EtOAc = 5/1) to give methyl 1-hydroxy-8-morpholino-2-naphthoate (**7d**) (5.4 mg, 19 µmol, 19%) as a colorless solid.

A typical procedure for arylative aromatization [method C]

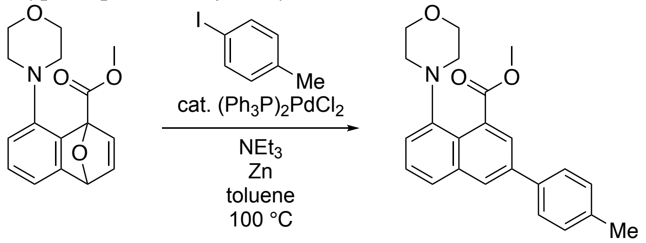

To a solution of methyl 8-morpholino-1,4-epoxynaphthalene-1(4*H*)-carboxylate (**4d**) (28.4 mg, 0.0988 mmol, 1.0 equiv) in toluene (2.0 mL) was added 1-iodo-4-methylbenzene (21.8 mg, 0.100 mmol, 1.0 equiv), triethylamine (140 µL, 1.00 mmol, 10 equiv), Zn powder (32.2 mg, 0.493 mmol, 5.0 equiv), and $(Ph_3P)_2PdCl_2$ (3.5 mg, 5.0 µmol, 5 mol %) at room temperature. After stirring for 24 h at 100 °C, the mixture was cooled to room temperature. The mixture was filtered with a short pad of celite. After filtration, the filtrate was concentrated under reduced pressure. The residue was purified by preparative TLC (*n*-hexane/EtOAc = 3/1) to give methyl 8-morpholino-3-(*p*-tolyl)-1-naphthoate (**7e**) (20.3 mg, 56.2 µmol, 56%) as a colorless solid.

*Synthesis of aryl sulfonate **15***

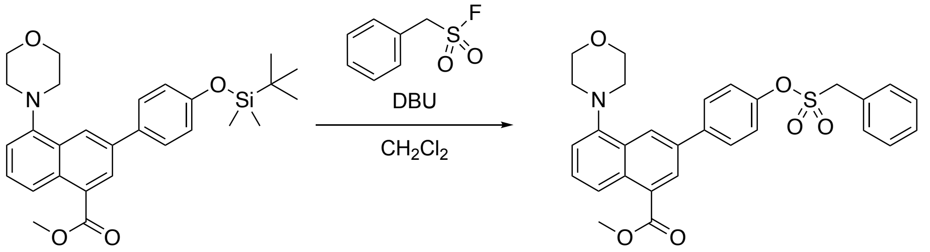

To a solution of methyl 3-(4-((*tert*-butyldimethylsilyl)oxy)phenyl)-5-morpholino-1-naphthoate (**7r**) (7.0 mg, 15 µmol, 1.0 equiv) in CH_2Cl_2 (200 µL) were added phenylmethanesulfonyl fluoride (**14b**) (3.1 mg, 18 µmol, 1.2

equiv) and 1,8-diazabicyclo[5.4.0]-7-undecene (DBU) (2.7 mg, 18 µmol, 1.2 equiv) at room temperature. After stirring for 24 h at the same temperature, the mixture was concentrated under reduced pressure. The residue was purified by preparative TLC (*n*-hexane/EtOAc = 3/1) to give methyl 3-(4-((benzylsulfonyl)oxy)phenyl)-5-morpholino-1-naphthoate (**15**) (5.7 mg, 11 µmol, 75%) as a yellow solid.

*A gram-scale synthesis of silyl ether **7r***

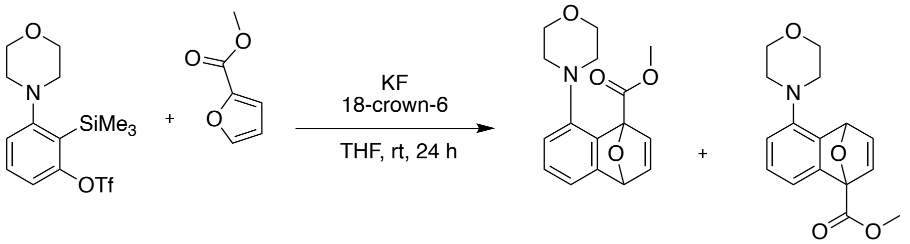

To a solution of 3-morpholino-2-(trimethylsilyl)phenyl trifluoromethanesulfonate (**2a**) (18.4 g, 50.0 mmol, 1.0 equiv) and methyl furan-2-carboxylate (31.6 g, 251 mmol, 5.0 equiv) dissolved in THF (500 mL) were added potassium fluoride (8.72 g, 150 mmol, 3.0 equiv) and 18-crown-6-ether (39.8 g, 150 mmol, 3.0 equiv) at room temperature. After stirring for 24 h at the same temperature, to the mixture was added water (150 mL). The mixture was extracted with EtOAc (100 mL × 3). The combined organic extract was washed with brine (100 mL) and dried with Na_2SO_4. After filtration, the filtrate was concentrated under reduced pressure. The residue was purified by recrystallization (*n*-hexane/EtOAc = 3/1) and the resulting mother liquor, after concentration under reduced pressure, was purified by flash column chromatography (silica-gel 200 g, *n*-hexane/EtOAc = 2/1) to give methyl 8-morpholino-1,4-epoxynaphthalene-1(4*H*)-carboxylate (**4d**) (8.39 g, 29.2 mmol, 59%) as a colorless solid and methyl 5-morpholino-1,4-epoxynaphthalene-1(4*H*)-carboxylate (**5d**) (1.43 g, 5.00 mmol, 10%) as a colorless oil.

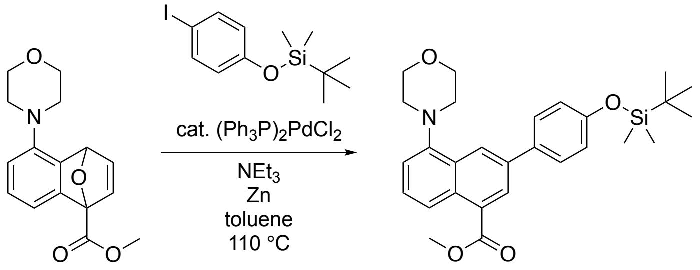

To a solution of methyl 5-morpholino-1,4-epoxynaphthalene-1(4*H*)-carboxylate (**5d**) (1.29 g, 4.50 mmol, 1.0 equiv) in toluene (90 mL) were added *tert*-butyl(4-iodophenoxy)dimethylsilane (1.50 g, 4.50 mmol, 1.0 equiv), triethylamine (4.54 g, 45.2 mmol, 10 equiv), Zn powder (1.48 g, 22.6 mmol, 5.0 equiv), and $(Ph_3P)_2PdCl_2$ (158 mg, 0.225 mmol, 5 mol %) at room temperature. After stirring for 24 h at 110 °C, the mixture was cooled to room temperature. The mixture was filtered with a short pad of celite. After filtration, the filtrate was concentrated under reduced pressure. The residue was purified by flash column chromatography (silica-gel 91.6 g, *n*-hexane/EtOAc = 5/1) to give methyl 3-(4-((*tert*-butyldimethylsilyl)oxy)phenyl)-5-morpholino-1-naphthoate (**7r**) (1.24 g, 2.59 mmol, 57%) as a yellow solid.

Absorption and fluorescent properties and spectra

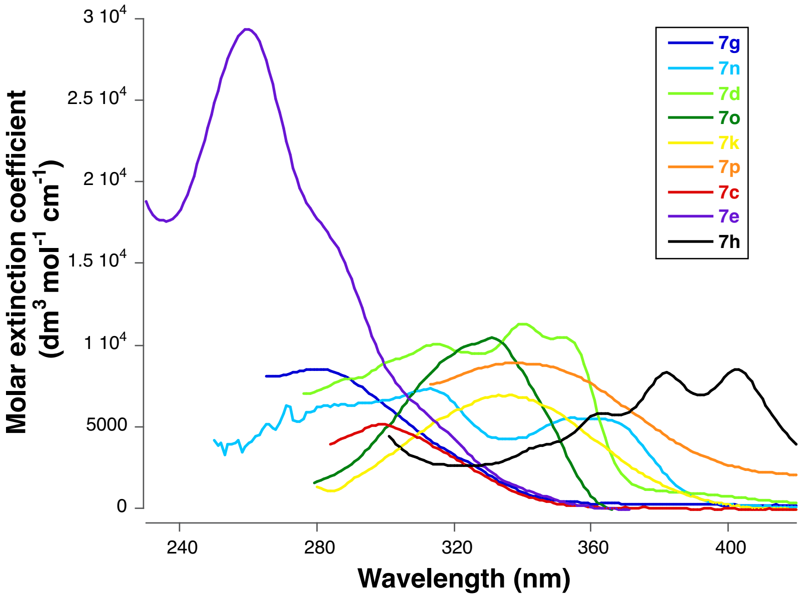

Fig. S1: UV-Vis spectra of **7g, 7n, 7d, 7o, 7k, 7p, 7c, 7e,** and **7h** in CH_2Cl_2 and summary table of observed representative absorption peaks **7g, 7n, 7d, 7o, 7k, 7p, 7c, 7e,** and **7h**. Concentrations: **7g** = 4.4 × 10^{-6} M, **7n** = 1.0 × 10^{-5} M, **7d** = 5.2 × 10^{-6} M, **7o** = 3.9 × 10^{-6} M, **7k** = 3.7 × 10^{-6} M, **7p** = 5.5 × 10^{-6} M, **7c** = 4.4 × 10^{-6} M, **7e** = 3.5 × 10^{-6} M, **7h** = 4.4 × 10^{-6} M.

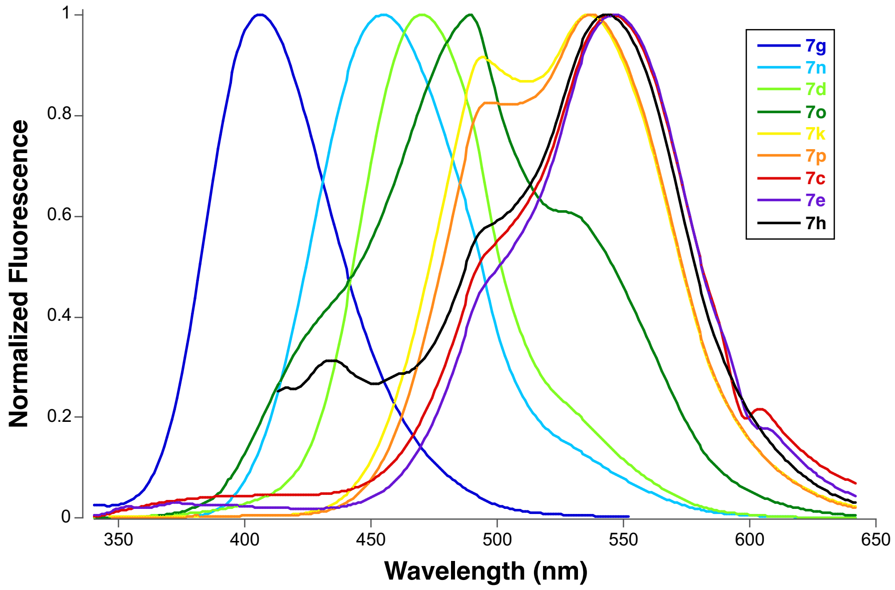

Fig. S2: Emission spectra of **7g, 7n, 7d, 7o, 7k, 7p, 7c, 7e,** and **7h** in CH_2Cl_2.
Excitation wavelengths for each compound: **7g** (281 nm), **7n** (355 nm), **7d** (326 nm), **7o** (331 nm), **7k** (336 nm), **7p** (337 nm), **7c** (298 nm), **7e** (300 nm), and **7h** (403 nm). Concentrations: **7g** = 4.4 × 10^{-6} M, **7n** = 1.0 × 10^{-5} M, **7d** = 5.2 × 10^{-6} M, **7o** = 3.9 × 10^{-6} M, **7k** = 3.7 × 10^{-6} M, **7p** = 5.5 × 10^{-6} M, **7c** = 4.4 × 10^{-6} M, **7e** = 3.5 × 10^{-6} M, **7h** = 4.4 × 10^{-6} M. The excitation wavelength was 281 nm (**7g**), 355 nm (**7n**), 326 nm (**7d**), 331 nm (**7o**), 336 nm (**7k**), 337 nm (**7p**), 298 nm (**7c**), 300 nm (**7e**), or 403 nm (**7h**).

Computational Methods

Geometry optimizations and frequency calculations were performed at B3LYP-D3/6-311+G(d,p) level of theory with Spartan 18 program (Wavefunction,. Inc. Irvine, CA) in the gas phase unless otherwise noted. Cartesian coordinates obtained by the DFT calculation with B3LYP-D3/6-311+G(d,p) were shown as calculated geometries described below. All the stationary geometries were confirmed to be energy minima by achieving vibrational frequency analyses. Transition structures were also confirmed to be true transition states on the potential energy surfaces by achieving vibrational frequency analyses.

Calculated Geometries

Optimized structure of **IIa**

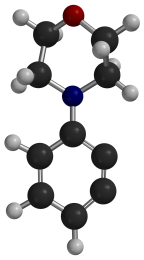

black: carbon, grey: hydrogen, red: oxygen, blue: nitrogen

G = –517.511568 hartrees

O	-3.149701	-0.057414	0.434545
C	-2.596404	-0.218013	-0.868299
H	-2.689262	0.717320	-1.439180
H	-3.201671	-0.977230	-1.367125
C	-2.246545	0.494939	1.401526
H	-1.856116	-0.315698	2.028938
H	-2.826319	1.172657	2.033759
C	-1.080869	1.236220	0.759074
H	-0.406950	1.598053	1.537138
C	-1.137322	-0.657407	-0.840770
H	-1.052327	-1.646472	-0.365906
H	-0.780056	-0.751840	-1.872939
H	-1.441898	2.110277	0.204799
N	-0.342327	0.333256	-0.121392
C	1.017309	0.194735	-0.012503
C	3.945726	-0.041445	0.237352
C	1.776666	-0.811091	-0.683924
C	1.849618	1.011504	0.765238
C	3.081932	0.861548	0.819715
C	3.169241	-0.912513	-0.559862
H	1.269616	-1.529139	-1.316571
H	3.678524	-1.702828	-1.104883
H	5.019135	-0.109420	0.331267

Optimized structure of **3d**

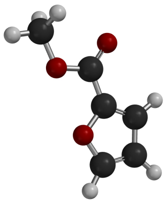

black: carbon, grey: hydrogen, red: oxygen

G = –457.963208 hartrees

C	-2.567592	0.070554	-0.418493
H	-3.507203	0.134141	0.105361
C	-2.240069	-0.015959	-1.739714
H	-2.928584	-0.037438	-2.569365

C	-0.817229	-0.070073	-1.787517
H	-0.179472	-0.142018	-2.653294
C	-0.387540	-0.012766	-0.491572
O	-1.458125	0.073590	0.354594
C	0.973361	-0.032660	0.044518
O	1.959531	-0.104762	-0.655656
O	0.996349	0.037800	1.388075
C	2.304416	0.026328	1.987777
H	2.831408	-0.895360	1.735031
H	2.130595	0.089340	3.059692
H	2.890153	0.879282	1.640564

Optimized structure of **3r**

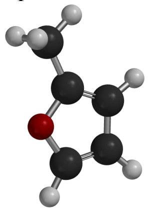

black: carbon, grey: hydrogen, red: oxygen

G = –269.357106 hartrees

C	0.378077	1.787349	-0.181440
H	0.585593	2.803383	0.108634
C	0.273185	1.156303	-1.377849
H	0.390179	1.604817	-2.351631
C	-0.022263	-0.217006	-1.079563
H	-0.174847	-1.022505	-1.780817
C	-0.075097	-0.320628	0.276245
O	0.168709	0.902298	0.839030
C	-0.330704	-1.451145	1.206565
H	0.527582	-1.627504	1.862285
H	-0.520950	-2.361822	0.636489
H	-1.199463	-1.253541	1.842053

Transition structure **TS1d**

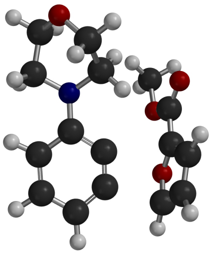

black: carbon, grey: hydrogen, red: oxygen, blue: nitrogen

G = –975.452992 hartrees

Imaginary frequency: *i* 247 cm^{-1}

O	-3.115196	-2.613703	0.543505
C	-2.823103	-2.464724	-0.844251
H	-3.271480	-1.536350	-1.227268
H	-3.299749	-3.304523	-1.354532
C	-2.175495	-1.968557	1.414045
H	-1.455158	-2.712944	1.779625
H	-2.741073	-1.589165	2.268823
C	-1.431597	-0.830208	0.726547
H	-0.719218	-0.383906	1.421567
C	-1.326407	-2.462079	-1.130386
H	-0.890372	-3.422687	-0.813430

H	-1.170865	-2.369482	-2.211581
H	-2.135877	-0.040162	0.446153
N	-0.705451	-1.337137	-0.441645
C	0.643307	-1.082933	-0.586868
C	3.483794	-0.506104	-0.853245
C	1.502630	-1.854532	-1.419562
C	1.314206	-0.035038	0.050363
C	2.558768	0.185010	-0.088869
C	2.867558	-1.567070	-1.541985
H	1.105113	-2.698561	-1.970965
H	3.470832	-2.194006	-2.192358
H	4.538675	-0.283566	-0.948994
C	2.749936	1.977571	2.080535
H	3.502075	1.679097	2.793414
C	2.972982	2.249437	0.727343
H	3.879139	2.494866	0.196872
C	1.369550	2.015456	2.258242
H	0.791005	1.764803	3.132319
C	0.831168	2.345421	1.017931
O	1.815675	2.687073	0.156452
C	-0.582043	2.518694	0.639896
O	-0.730716	2.895219	-0.637730
C	-2.088063	3.010653	-1.104713
H	-2.644733	3.726302	-0.497497
H	-2.585412	2.039421	-1.064050
H	-2.009209	3.354303	-2.133727
O	-1.495197	2.314111	1.410022

Transition structure **TS1r**

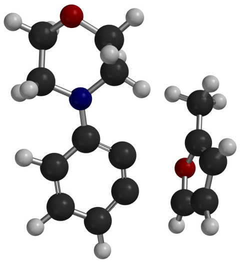

black: carbon, grey: hydrogen, red: oxygen, blue: nitrogen

G = –786.849899 hartrees

Imaginary frequency: *i* 163 cm^{-1}

O	-3.481956	-2.272891	0.218515
C	-2.940816	-2.493205	-1.081314
H	-3.283956	-1.709905	-1.772044
H	-3.347321	-3.446193	-1.425557
C	-2.736495	-1.348736	1.023638
H	-2.149933	-1.914789	1.759319
H	-3.463148	-0.733344	1.563021
C	-1.801953	-0.467098	0.204589
H	-1.218163	0.166177	0.875187
C	-1.416656	-2.544039	-1.088657
H	-1.068227	-3.404290	-0.496633
H	-1.079116	-2.697341	-2.120963
H	-2.384972	0.200911	-0.446499
N	-0.885680	-1.292890	-0.567969
C	0.456130	-0.977401	-0.657290
C	3.258866	-0.260217	-0.879620
C	1.413917	-1.844462	-1.254330
C	0.998688	0.220364	-0.171147
C	2.234972	0.475867	-0.314750

C	2.763340	-1.485993	-1.361754
H	1.111268	-2.814943	-1.630368
H	3.449506	-2.183969	-1.832927
H	4.297582	0.029773	-0.971885
C	2.545838	2.289961	1.743061
H	3.348569	1.939413	2.372309
C	2.652273	2.600221	0.396672
H	3.502754	2.819973	-0.227370
C	1.179519	2.377250	2.057607
H	0.697689	2.118311	2.987073
C	0.533175	2.787312	0.907121
O	1.444134	3.057552	-0.057286
C	-0.883972	3.133746	0.616996
H	-0.996678	4.213220	0.469285
H	-1.519822	2.832599	1.451289
H	-1.229359	2.629055	-0.287320

Optimized structure of **4d**

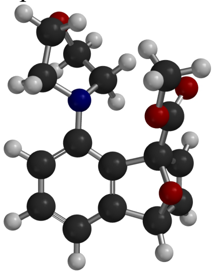

black: carbon, grey: hydrogen, red: oxygen, blue: nitrogen

G = –975.525402 hartrees

C	0.797971	0.563163	0.594230
C	1.300573	0.011805	3.360451
C	0.093195	-0.401057	1.300800
C	1.830143	1.219929	1.308723
C	2.071038	0.947715	2.649902
C	0.320145	-0.652898	2.655753
H	2.443921	1.957451	0.808402
H	2.869808	1.477495	3.156975
H	1.493248	-0.194118	4.406671
C	-0.994494	-1.434207	0.908348
C	-0.664612	-1.779322	3.001430
H	-0.431746	-2.409733	3.855349
O	-0.598052	-2.531496	1.764461
C	-2.295960	-0.990482	1.600650
H	-3.138095	-0.557673	1.082582
C	-2.089725	-1.199827	2.898382
H	-2.731380	-0.965365	3.735137
N	0.493976	0.828610	-0.755809
C	1.542181	1.399274	-1.603472
H	1.940533	2.348619	-1.214202
H	2.375917	0.693727	-1.668720
C	0.971134	1.668025	-2.988361
H	0.568791	0.737188	-3.411887
H	1.753559	2.037101	-3.655152
C	-0.863440	1.328190	-1.082138
H	-1.458869	1.381012	-0.168557
H	-1.373195	0.640673	-1.761003
C	-0.785791	2.706763	-1.729148
H	-0.322239	3.424916	-1.039381
H	-1.786835	3.067990	-1.975616
O	-0.047320	2.670682	-2.956375

C	-1.104703	-1.851110	-0.544856
O	-2.055224	-1.589333	-1.244121
O	-0.030180	-2.529183	-0.950378
C	-0.031814	-2.930654	-2.334243
H	-0.896503	-3.560781	-2.548615
H	0.894167	-3.482900	-2.476741
H	-0.060123	-2.050189	-2.979472

Optimized structure of **4r**

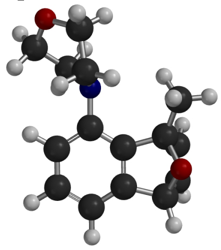

black: carbon, grey: hydrogen, red: oxygen, blue: nitrogen

G = –786.921353 hartrees

C	0.266653	0.550921	0.414757
C	0.918743	0.107070	3.152163
C	0.071943	-0.679738	1.013929
C	0.827585	1.574778	1.215279
C	1.139853	1.360694	2.551006
C	0.391597	-0.891103	2.364500
H	1.019654	2.544913	0.769415
H	1.570687	2.167086	3.134674
H	1.160421	-0.058936	4.196405
C	-0.531908	-2.030205	0.544193
C	-0.039641	-2.340577	2.628860
H	0.444733	-2.871057	3.445479
O	0.278883	-2.929326	1.352274
C	-1.881116	-2.151228	1.290428
H	-2.848602	-2.024090	0.824069
C	-1.581495	-2.356575	2.570557
H	-2.240807	-2.436803	3.423350
N	-0.062550	0.746803	-0.964786
C	1.072751	0.535802	-1.879151
H	1.965974	1.098561	-1.562075
H	1.342675	-0.523397	-1.885494
C	0.682016	0.973014	-3.283228
H	-0.199279	0.404232	-3.612232
H	1.497515	0.774225	-3.982961
C	-0.847144	1.951729	-1.302131
H	-1.202600	2.427620	-0.385556
H	-1.733090	1.654161	-1.876478
C	-0.043767	2.951528	-2.130913
H	0.817224	3.316704	-1.552546
H	-0.660753	3.813793	-2.398143
O	0.407004	2.376583	-3.359015
C	-0.508391	-2.431359	-0.909611
H	0.522099	-2.516958	-1.259409
H	-1.024835	-1.685334	-1.514854
H	-0.992033	-3.403530	-1.032756

Transition structure **TS2d**

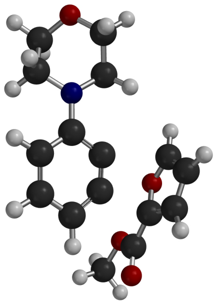

black: carbon, grey: hydrogen, red: oxygen, blue: nitrogen

G = –975.451159 hartrees

O	-4.509454	-2.138499	0.091062
C	-3.670800	-2.478670	-1.009636
H	-3.650217	-1.653312	-1.735261
H	-4.130497	-3.342792	-1.494304
C	-3.846273	-1.414472	1.133384
H	-3.585444	-2.109680	1.942390
H	-4.564512	-0.688214	1.523444
C	-2.585838	-0.701802	0.658997
H	-2.117443	-0.198944	1.508303
C	-2.242670	-2.812933	-0.588398
H	-2.237886	-3.715202	0.038806
H	-1.664903	-3.034447	-1.491263
H	-2.839465	0.076304	-0.077921
N	-1.651916	-1.675777	0.112682
C	-0.315691	-1.348804	-0.020430
C	2.475977	-0.621228	-0.340517
C	0.656093	-2.263679	-0.515807
C	0.210679	-0.100257	0.336694
C	1.445936	0.159848	0.142643
C	1.998283	-1.903238	-0.672421
H	0.366908	-3.281601	-0.752010
H	2.694285	-2.643563	-1.055866
H	3.507501	-0.317962	-0.460718
C	1.851490	1.990241	2.084835
H	2.748184	1.736000	2.625968
C	1.800914	2.258055	0.709219
C	0.530114	1.974886	2.523000
H	0.156949	1.701020	3.496759
C	-0.245383	2.269471	1.407853
O	0.530022	2.643094	0.375424
H	-1.305075	2.425483	1.287845
C	2.911892	2.547307	-0.216910
O	4.062316	2.276016	0.042645
O	2.488342	3.081197	-1.369939
C	3.504472	3.286759	-2.371950
H	3.962669	2.333760	-2.643845
H	4.275298	3.965402	-2.001028
H	2.985142	3.720232	-3.223728

Transition structure **TS2r**

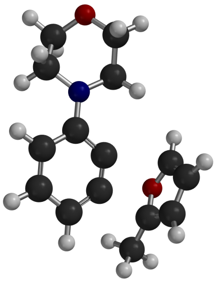

black: carbon, grey: hydrogen, red: oxygen, blue: nitrogen

G = –786.848171 hartrees

Imaginary frequency: *i* 174 cm^{-1}

O	-4.128803	-1.952108	-0.017000
C	-3.350365	-2.238783	-1.175511
H	-3.386003	-1.389919	-1.872967
H	-3.822972	-3.095350	-1.661372
C	-3.422436	-1.239565	1.005402
H	-3.107721	-1.948730	1.782844
H	-4.130729	-0.535514	1.450977
C	-2.202799	-0.491969	0.481764
H	-1.692911	-0.004649	1.316109
C	-1.894319	-2.558083	-0.849167
H	-1.836916	-3.479188	-0.252146
H	-1.366363	-2.740739	-1.790702
H	-2.511349	0.303449	-0.215369
N	-1.285764	-1.431548	-0.147833
C	0.036923	-1.077846	-0.336503
C	2.792331	-0.280511	-0.765182
C	0.996463	-1.952042	-0.921212
C	0.565117	0.161410	0.043898
C	1.780170	0.461864	-0.187342
C	2.321565	-1.556008	-1.131809
H	0.713209	-2.963397	-1.191011
H	3.008426	-2.262689	-1.588898
H	3.811880	0.037197	-0.943169
C	2.188152	2.326981	1.742939
H	3.090439	2.068248	2.274767
C	2.120971	2.647468	0.387475
C	0.868416	2.271246	2.213386
H	0.524643	1.953430	3.184833
C	0.068357	2.582642	1.134005
O	0.824144	2.970813	0.082759
H	-0.996870	2.700867	1.025030
C	3.156865	3.094930	-0.584590
H	4.103870	2.598050	-0.369478
H	3.309831	4.177100	-0.522598
H	2.854547	2.842942	-1.602328

Optimized structure of **5d**

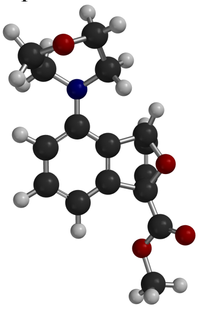

black: carbon, grey: hydrogen, red: oxygen, blue: nitrogen

G = –975.521883 hartrees

C	-0.178184	1.063267	-0.509387
C	0.590880	0.548072	2.214317
C	-0.156411	-0.223335	0.026573
C	0.222163	2.103249	0.371252
C	0.583479	1.850116	1.687235
C	0.229529	-0.460619	1.350320
H	0.274455	3.121802	0.010217
H	0.884480	2.681842	2.314233
H	0.860778	0.349079	3.243253
C	-0.594985	-1.613742	-0.482514
H	-0.577015	-1.818136	-1.545903
C	0.029009	-1.986806	1.524679
O	0.362425	-2.443432	0.208456
C	-1.886081	-1.980106	0.277458
H	-2.882355	-1.965209	-0.140794
C	-1.505972	-2.209638	1.530255
H	-2.089139	-2.421682	2.413040
N	-0.583563	1.302843	-1.826616
C	-0.185312	0.372396	-2.893177
H	0.680737	-0.200395	-2.553626
H	-0.988959	-0.336184	-3.143353
C	0.210266	1.146891	-4.159566
H	-0.674813	1.470381	-4.726102
H	0.798038	0.501621	-4.813938
C	-0.855893	2.648790	-2.306934
H	-1.155040	3.294062	-1.482283
H	-1.719599	2.604163	-2.979383
C	0.354468	3.242969	-3.047424
H	1.095715	3.626171	-2.341719
H	0.028460	4.069374	-3.695319
O	1.034135	2.251709	-3.828133
C	0.832426	-2.697998	2.584980
O	1.703351	-3.500279	2.390777
O	0.415092	-2.300882	3.807238
C	1.092573	-2.915080	4.922667
H	2.160309	-2.693037	4.887681
H	0.953010	-3.997107	4.899483
H	0.637544	-2.485132	5.812056

Optimized structure of **5r**

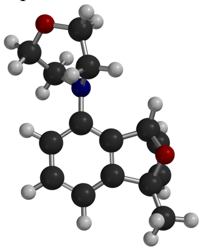

black: carbon, grey: hydrogen, red: oxygen, blue: nitrogen

G = –786.922999 hartrees

C	0.063867	-0.020554	-0.691083
C	0.761840	2.710104	-0.236621
C	-0.004642	0.554920	0.566356
C	0.533427	0.806201	-1.738674
C	0.864661	2.137446	-1.516892
C	0.331297	1.894326	0.788257
H	0.640303	0.395799	-2.736359
H	1.217102	2.738720	-2.348085
H	1.010335	3.752795	-0.076010
C	-0.523583	0.066112	1.922878
H	-0.478712	-0.997110	2.136277
C	-0.003316	2.135432	2.270554
O	0.334974	0.825362	2.802570
C	-1.867621	0.787729	2.146802
H	-2.841826	0.330740	2.049325
C	-1.548681	2.062560	2.355291
H	-2.198418	2.918668	2.471474
N	-0.311912	-1.382205	-0.874214
C	0.692872	-2.355010	-0.403777
H	1.699164	-2.097141	-0.769724
H	0.734823	-2.352804	0.688745
C	0.334255	-3.749733	-0.895422
H	-0.659934	-4.033833	-0.523206
H	1.056984	-4.476832	-0.519574
C	-0.926608	-1.775347	-2.153902
H	-1.227236	-0.887590	-2.712139
H	-1.841197	-2.344501	-1.949432
C	0.003787	-2.638450	-3.001222
H	0.913257	-2.077408	-3.258972
H	-0.486827	-2.936093	-3.932379
O	0.366082	-3.846288	-2.322218
C	0.707429	3.252343	2.985106
H	1.788077	3.114583	2.912230
H	0.419756	3.277074	4.037564
H	0.446222	4.209985	2.526477

Characterization Data of New Compounds

1-Morpholinonaphthalene (**7g**),[S4] 1-morpholino-5-(methoxycarbonyl)naphthalene (**7k**),[S5] ethyl 8-methoxy-1,4-epoxynaphthalene-1(4*H*)-carboxylate (**9a**),[S6] and ethyl 8-methoxy-1,4-epoxynaphthalene-1(4*H*)-carboxylate (**10a**)[S4] were identical in spectra data with those reported in the literature.

3-((4a*S**,8a*R**)-Octahydroisoquinolin-2(1*H*)-yl)-2-(trimethylsilyl)phenyl trifluoromethanesulfonate (**2c**)

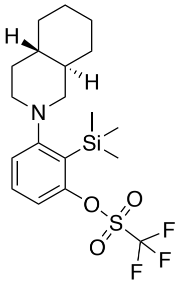

Yield: 59% (1.28 g, 2.95 mmol); Colorless oil; TLC R_f 0.49 (*n*-hexane/EtOAc = 10/1); ^{1}H NMR ($CDCl_3$, 400 MHz): δ 7.38 (dd, 1H, *J* = 8.1, 8.1 Hz), 7.26 (d, 1H, *J* = 8.1 Hz), 7.09 (d, 1H, *J* = 8.1 Hz), 3.00–2.63 (m, 3H), 2.35 (t, 1H, *J* = 2.7 Hz), 1.82–1.55 (m, 4H), 1.55–1.22 (m, 5H), 1.14–0.91 (m, 3H), 0.39 (s, 9H); ^{13}C{^{1}H} NMR ($CDCl_3$, 101 MHz): δ 163.3, 154.7, 131.2, 131.0, 122.1, 118.6 (q, *J* = 312 Hz), 117.4, 61.7, 60.3, 55.7, 41.7, 41.5, 32.9, 30.4, 26.4, 26.0, 1.7; ^{19}F{^{1}H} NMR ($CDCl_3$, 377 MHz): δ –72.9 (s); IR (NaCl, cm^{-1}) 846, 957, 1140, 1212, 1248, 1418, 1422, 2923; HRMS (ESI) *m/z*: [M+H]$^+$ Calcd for $C_{19}H_{29}F_3NO_3SSi^+$ 436.1590; Found 436.1582.

4-(1,4-Diphenyl-1,4-dihydro-1,4-epoxynaphthalen-5-yl)morpholine (**4a**)

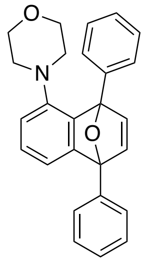

Yield: quant. (75.7 mg, 0.197 mmol); Colorless solid; Mp 174–177 °C; TLC R_f 0.25 (*n*-hexane/EtOAc = 6/1); ^{1}H NMR ($CDCl_3$, 400 MHz): δ 7.79–7.72 (m, 2H), 7.72–7.65 (m, 2H), 7.59–7.39 (m, 8H), 7.00 (dd, 1H, *J* = 8.1, 7.1 Hz), 6.77 (d, 1H, *J* = 6.6 Hz), 6.66 (d, 1H, *J* = 8.1 Hz), 3.36–3.26 (AA'BB', 2H), 3.13–2.89 (AA'BB', 2H), 2.83–2.65 (m, 4H); ^{13}C{^{1}H} NMR ($CDCl_3$, 101 MHz): δ 154.7, 147.5, 146.1, 142.7, 140.6, 135.6, 134.6, 129.6, 128.9, 128.4, 128.1, 127.9, 126.6, 126.4, 115.3, 114.8, 94.4, 93.3, 66.0, 51.6 (br); IR (NaCl, cm^{-1}) 1112, 1265, 1449, 2963, 3383, 3425, 3440, 3478; HRMS (ESI) *m/z*: [M+Na]$^+$ Calcd for $C_{26}H_{23}NNaO_2^+$ 404.1627; Found 404.1623.

4-(1,4-Dihydro-1,4-epoxynaphthalen-5-yl)morpholine (**4b**)

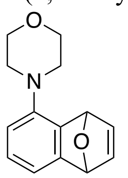

Yield: 38% (16.9 g, 75.0 μmol); Colorless solid; Mp 77–80 °C; TLC R_f 0.23 (*n*-hexane/EtOAc = 6/1); ^{1}H NMR ($CDCl_3$, 400 MHz): δ 7.11–7.05 (m, 2H), 6.99–6.92 (m, 2H), 6.60 (dd, 1H, *J* = 6.9, 2.1 Hz), 5.89 (s, 1H), 5.69 (s, 1H), 3.97–3.80 (AA'BB', 4H), 3.07–2.95 (AA'BB', 4H); ^{13}C{^{1}H} NMR ($CDCl_3$, 101 MHz): δ 150.3, 145.8, 143.5, 142.0, 139.3, 126.3, 115.0, 114.8, 82.3, 81.2, 67.0, 51.7; IR (NaCl, cm^{-1}) 856, 973, 1233, 1476, 1601, 2822, 2855, 2962; HRMS (ESI) *m/z*: [M+H]$^+$ Calcd for $C_{14}H_{16}NO_2^+$ 230.1181; Found 230.1172.

4-(9,10-Diphenyl-9,10-dihydro-9,10-epoxyanthracen-1-yl)morpholine (**4c**)

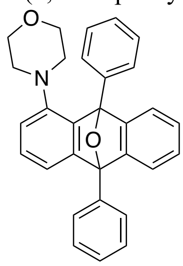

Yield: quant. (43.0 mg, 99.6 μmol); Colorless solid; Mp 188−191 °C; TLC R_f 0.60 (*n*-hexane/EtOAc = 3/1); ^{1}H NMR ($CDCl_3$, 400 MHz): δ 7.99–7.94 (m, 2H), 7.87–7.82 (m, 2H), 7.57–7.48 (m, 6H), 7.47–7.38 (m, 2H), 7.17–7.00 (m, 4H), 6.64 (dd, 1H, J = 6.7, 2.3 Hz), 3.44–3.36 (AA'BB', 2H), 3.20–3.00 (AA'BB', 2H), 2.96–2.86 (AA'BB', 2H), 2.89–2.76 (AA'BB', 2H); ^{13}C{^{1}H} NMR ($CDCl_3$, 101 MHz): δ 153.0, 152.4, 148.2, 146.2, 139.2, 134.9, 133.0, 130.7, 129.3, 128.5, 128.1, 128.0, 127.2, 126.8, 125.6, 125.5, 121.8, 120.8, 115.5, 114.6, 92.4, 89.8, 66.0, 50.9; IR (NaCl, cm^{-1}) 960, 1115, 1266, 1448, 1478, 2822, 2857, 2959; HRMS (ESI) *m/z*: [M+H]$^+$ Calcd for $C_{30}H_{26}NO_2^+$ 432.1958; Found 432.1957.

Methyl 8-morpholino-1,4-epoxynaphthalene-1(4*H*)-carboxylate (**4d**)

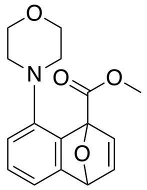

Yield: 40% (1.13 g, 3.93 mmol); Colorless solid; Mp 147–149 °C; TLC R_f 0.26 (*n*-hexane/EtOAc = 6/1); ^{1}H NMR ($CDCl_3$, 400 MHz): δ 7.29–7.23 (m, 1H), 7.12–6.99 (m, 3H), 6.77 (dd, 1H, J = 8.0, 0.9 Hz), 5.77 (d, 1H, J = 1.9 Hz), 3.97 (s, 3H), 3.90–3.82 (m, 2H), 3.74–3.66 (m, 2H), 2.95–2.85 (m, 2H), 2.79–2.70 (m, 2H); ^{13}C{^{1}H} NMR ($CDCl_3$, 101 MHz): δ 168.3, 149.6, 146.4, 144.2, 142.1, 140.5, 127.3, 117.0, 116.7, 90.4, 83.2, 66.9, 53.0 (br), 52.8; IR (NaCl, cm^{-1}) 939, 1115, 1248, 1348, 1475, 1743, 1746, 2955; HRMS (ESI) *m/z*: [M+Na]$^+$ Calcd for $C_{16}H_{17}NNaO_4^+$ 310.1055; Found 310.1056.

Recrystallization was carried out by slow evaporation of a solution of 4d in ethyl acetate in an inner vial, placed in an outer vial containing silica gel. Selected crystal data of **4d** recorded on a Rigaku R-axis Rapid II imaging plate diffractometer equipped with Cu Kα rotating anode X-ray tube. Data collection, integration, and scaling were performed using the Rigaku RAPID AUTO software: triclinic, P −1 (No. 2), a = 6.1178(4) Å, b = 8.1302(5) Å, c =14.8205(10) Å, V = 676.00(8) Å^3, Z = 2, R1 = 0.0451, wR_2 = 0.1133, CCDC 2503248.

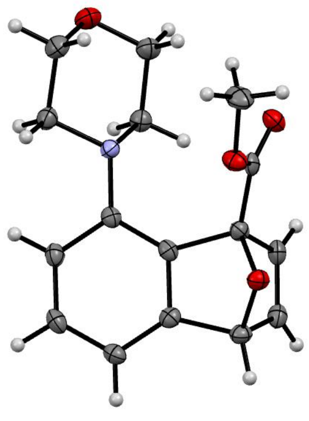

ORTEP drawing of **4d** (50% probability ellipsoids); grey: carbon, white: hydrogen, red: oxygen, blue: nitrogen.

Methyl 5-morpholino-1,4-epoxynaphthalene-1(4*H*)-carboxylate (**5d**)

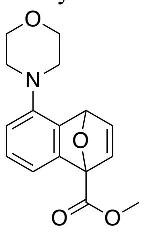

Yield: 8% (235 mg, 0.82 mmol); Colorless oil; TLC R_f 0.16 (*n*-hexane/EtOAc = 6/1); ^{1}H NMR ($CDCl_3$, 400 MHz): δ 7.14–7.08 (m, 2H), 7.04 (d, 1H, *J* = 8.0 Hz), 6.97 (dd, 1H, *J* = 8.0, 8.0 Hz), 6.64 (d, 1H, *J* = 8.0 Hz), 5.97 (d, 1H, *J* = 1.5 Hz), 4.00–3.76 (m, 3H + 4H), 3.08–2.91 (m, 4H); $^{13}C\{^1H\}$ NMR ($CDCl_3$, 101 MHz): δ 168.2, 148.5, 146.1, 142.9, 142.7, 138.1, 126.6, 115.9, 114.3, 90.3, 81.4, 67.0, 52.8, 51.8; IR (NaCl, cm^{-1}) 803, 962, 1121, 1232, 1447, 1739, 1761, 2958; HRMS (ESI) *m/z*: $[M+Na]^+$ Calcd for $C_{16}H_{17}NNaO_4^+$ 310.1055; Found 310.1060.

We found that the regiochemistry can be predicted based on the chemical shift values of the morpholino protons and the protons at the 8- or 5-positions in the ^{1}H NMR spectrum.

4-(4-Butyl-1,4-dihydro-1,4-epoxynaphthalen-5-yl)morpholine (**4e**)

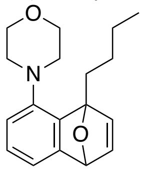

Yield: 58% (32.7 mg, 0.115 mmol); Brown oil; TLC R_f 0.52 (*n*-hexane/EtOAc = 6/1); ^{1}H NMR ($CDCl_3$, 400 MHz): δ 7.08–6.92 (m, 3H), 6.83 (d, 1H, *J* = 5.5 Hz), 6.75 (dd, 1H, *J* = 7.8, 1.1 Hz), 5.59 (d, 1H, *J* = 1.8 Hz), 3.96–3.88 (m, 2H), 3.85–3.75 (m, 2H), 2.97–2.76 (AA'BB', 4H), 2.57–2.31 (m, 2H), 1.71–1.41 (AA'BB', 4H), 0.97 (t, 3H, *J* = 7.1 Hz); $^{13}C\{^1H\}$ NMR ($CDCl_3$, 101 MHz): δ 152.4, 146.7, 144.9, 144.4, 142.5, 126.3, 116.8, 116.0, 94.1, 81.2, 67.1, 53.0, 30.2, 27.3, 23.4, 14.2; IR (NaCl, cm^{-1}) 886, 950, 1116, 1228, 1451, 1471, 2955, 2958; HRMS (ESI) *m/z*: $[M+H]^+$ Calcd for $C_{18}H_{24}NO_2{}^+$ 286.1802; Found 286.1802.

The regiochemistry of **4e** was determined by the NOESY experiments.

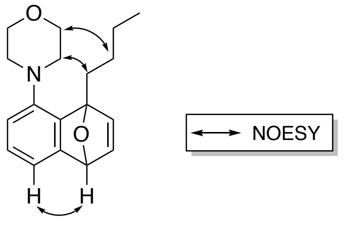

4-(1-Butyl-1,4-dihydro-1,4-epoxynaphthalen-5-yl)morpholine (**5e**)

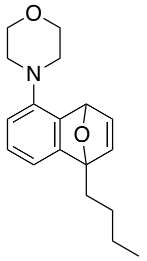

Yield: 13% (7.4 mg, 26 μmol); Brown oil; TLC R_f 0.25 (*n*-hexane/EtOAc = 6/1); ^{1}H NMR ($CDCl_3$, 400 MHz): δ 7.05 (dd, 1H, *J* = 5.4, 1.6 Hz), 6.96 (dd, 1H, *J* = 8.1, 8.1 Hz), 6.86 (br d, 1H, *J* = 8.1 Hz), 6.83 (d, 1H, *J* = 5.4 Hz), 6.59 (dd, 1H, *J* = 8.1, 0.5 Hz), 5.81 (d, 1H, *J* = 1.6 Hz), 3.95–3.78 (AA'BB', 4H), 3.06–2.94 (AA'BB', 4H), 2.38–2.17 (m, 2H), 1.69–1.39 (m, 4H), 0.96 (t, 3H, *J* = 7.2 Hz); $^{13}C\{^1H\}$ NMR ($CDCl_3$, 101 MHz): δ 152.0, 145.5, 145.3, 143.3, 141.3, 126.2, 114.8, 113.8, 92.7, 80.5, 67.1, 51.8, 29.0, 26.8, 23.2, 14.0; IR (NaCl, cm^{-1}) 1236, 1451, 1601, 1634, 2855, 2969, 3400, 3430, 3466; HRMS (ESI) *m/z*: $[M+Na]^+$ Calcd for $C_{18}H_{23}NNaO_2{}^+$ 308.1627; Found 308.1623.

8-Morpholino-1,4-epoxynaphthalene-1(4*H*)-carbonitrile (**4f**)

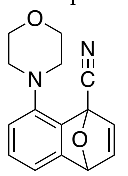

Yield: 39% (19.5 mg, 76.7 μmol); Colorless solid; Mp 188–190 °C; TLC R_f 0.24 (*n*-hexane/EtOAc = 6/1); ^{1}H NMR ($CDCl_3$, 400 MHz): δ 7.19 (dd, 1H, *J* = 5.4, 2.0 Hz), 7.11–6.99 (m, 3H), 6.76 (dd, 1H, *J* = 7.5, 1.5 Hz), 5.75 (d, 1H, *J* = 2.0 Hz), 4.10–3.95 (AA'BB', 2H), 3.95–3.81 (AA'BB', 2H), 3.05–2.87 (m, 4H); ^{13}C{^{1}H} NMR ($CDCl_3$, 101 MHz): δ 147.7, 146.7, 144.6, 141.0, 137.0, 127.9, 116.6, 116.0, 115.5, 82.8, 78.3, 66.4, 52.7; IR (NaCl, cm^{-1}) 980, 1112, 1263, 1455, 1733, 2856, 2956; HRMS (ESI) *m/z*: [M+Na]$^+$ Calcd for $C_{15}H_{14}N_2NaO_2^+$ 277.0953; Found 277.0951.

5-Morpholino-1,4-epoxynaphthalene-1(4*H*)-carbonitrile (**5f**)

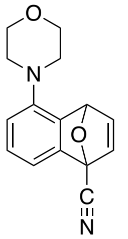

Yield: 16% (8.2 mg, 32 μmol); Yellow solid; Mp 101–104 °C; TLC R_f 0.23 (*n*-hexane/EtOAc = 6/1); ^{1}H NMR ($CDCl_3$, 400 MHz): δ 7.18 (dd, 1H, *J* = 5.4, 1.7 Hz), 7.15 (br d, 1H, *J* = 8.2 Hz), 7.09–7.03 (m, 2H), 6.69 (dd, 1H, *J* = 8.2, 0.5 Hz), 5.94 (d, 1H, *J* = 1.7 Hz), 3.94–3.79 (AA'BB', 4H), 3.08–2.94 (AA'BB', 4H); ^{13}C{^{1}H} NMR ($CDCl_3$, 101 MHz): δ 147.3, 146.4, 143.1, 141.8, 136.3, 127.3, 116.5, 115.2, 113.9, 82.1, 79.4, 66.9, 51.7; IR (NaCl, cm^{-1}) 923, 1118, 1238, 1265, 1478, 1603, 2827, 2855, 2923, 2959; HRMS (ESI) *m/z*: [M+Na]$^+$ Calcd for $C_{15}H_{14}N_2NaO_2{}^+$ 277.0953; Found 277.0944.

4-(3,4-Dimethyl-1,4-dihydro-1,4-epoxynaphthalen-5-yl)morpholine (**4h**)

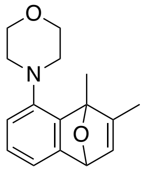

Yield: 44% (22.6 mg, 87.8 μmol); Yellow solid; Mp 130–134 °C; TLC R_f 0.40 (*n*-hexane/EtOAc = 6/1); ^{1}H NMR ($CDCl_3$, 400 MHz): δ 7.01–6.94 (m, 2H), 6.87–6.79 (m, 1H), 6.54–6.49 (m, 1H), 5.46 (d, 1H, *J* = 1.4 Hz), 3.93–3.78 (AA'BB', 4H), 2.96–2.88 (AA'BB', 4H), 2.01 (s, 3H), 1.86 (s, 3H); ^{13}C{^{1}H} NMR ($CDCl_3$, 101 MHz): δ 154.3, 153.3, 147.2, 143.7, 136.6, 126.5, 118.2, 116.0, 91.5, 80.4, 67.2, 53.7, 15.6, 13.2; IR (NaCl, cm^{-1}) 884, 1115, 1261, 1471, 1600, 2846, 2939, 2955; HRMS (ESI) *m/z*: [M+Na]$^+$ Calcd for $C_{16}H_{19}NNaO_2^+$ 280.1314; Found 280.1313.

4-(1,2-Dimethyl-1,4-dihydro-1,4-epoxynaphthalen-5-yl)morpholine (**5h**)

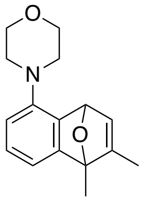

Yield: 26% (13.2 mg, 51.3 μmol); Beige solid; Mp 58–60 °C; TLC R_f 0.43 (*n*-hexane/EtOAc = 6/1); ^{1}H NMR ($CDCl_3$, 400 MHz): δ 6.96 (dd, 1H, *J* = 8.1, 8.1 Hz), 6.88 (d, 1H, *J* = 8.1 Hz), 6.62 (d, 1H, *J* = 8.1 Hz), 6.52 (br d, 1H, *J* = 1.3 Hz), 5.72 (d, 1H, *J* = 1.3 Hz), 3.94–3.79 (AA'BB', 4H), 3.05–2.94 (AA'BB', 4H), 1.82–1.76 (m, 6H); ^{13}C{^{1}H} NMR ($CDCl_3$, 101 MHz): δ 155.0, 152.5, 144.7, 141.6, 135.2, 125.8, 115.0, 113.0, 90.3, 79.6, 67.0, 51.7, 13.7, 12.9; IR (NaCl, cm^{-1}) 974, 1119, 1236, 1451, 1477, 1603, 2857, 2933, 2956, 3443; HRMS (ESI) *m/z*: [M+Na]$^+$ Calcd for $C_{16}H_{19}NNaO_2^+$ 280.1314; Found 280.1314.

The regiochemistry of **5h** was determined by the NOESY experiments.

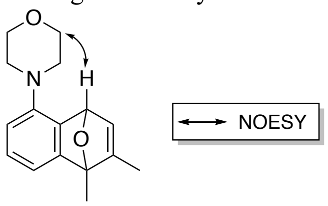

Methyl 4-bromo-8-morpholino-1,4-epoxynaphthalene-1(4*H*)-carboxylate (**4i**)

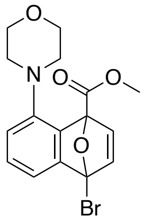

Yield: 31% (23.1 mg, 63.1 μmol); Colorless solid; Mp 141–143 °C; TLC R_f 0.22 (*n*-hexane/EtOAc = 6/1); ^{1}H NMR ($CDCl_3$, 400 MHz): δ 7.29 (d, 1H, *J* = 5.4 Hz), 7.20–7.08 (m, 2H), 7.03 (d, 1H, *J* = 5.4 Hz), 6.82 (dd, 1H, *J* = 7.9, 0.8 Hz), 3.97 (s, 3H), 3.87–3.80 (AA'BB', 2H), 3.74–3.65 (AA'BB', 2H), 2.92–2.83 (AA'BB', 2H), 2.79–2.72 (AA'BB', 2H); ^{13}C{^{1}H} NMR ($CDCl_3$, 101 MHz): δ 166.7, 149.4, 147.1, 146.1, 141.6, 140.6, 127.9, 118.0, 116.8, 91.4, 89.4, 66.9, 53.1, 53.0; IR (NaCl, cm^{-1}) 934, 1116, 1235, 1604, 1746, 2856, 2956, 3424; HRMS (ESI) *m/z*: [M+Na]$^+$ Calcd for $C_{16}H_{16}BrNNaO_4^+$ 388.0154 ; Found 388.0160

Methyl 4-bromo-5-morpholino-1,4-epoxynaphthalene-1(4*H*)-carboxylate (**5i**)

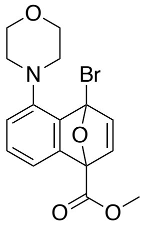

Yield: 31% (23.3 mg, 63.6 μmol); Yellow oil; TLC R_f 0.22 (*n*-hexane/EtOAc = 6/1); ^{1}H NMR ($CDCl_3$, 400 MHz): δ 7.13 (d, 1H, *J* = 5.2 Hz), 7.09–6.99 (m, 3H), 6.77 (dd, 1H, *J* = 7.1, 2.1 Hz), 4.03–3.94 (m, 2H + 3H), 3.88–3.79 (m, 2H), 3.03–2.87 (AA'BB', 4H); ^{13}C{^{1}H} NMR ($CDCl_3$, 101 MHz): δ 166.7, 147.6, 147.4, 145.7, 144.1, 136.8, 127.7, 117.4, 114.3, 89.8, 89.1, 66.7, 53.1, 52.8; IR (NaCl, cm^{-1}) 1039, 1119, 1447, 1641, 2085, 2962, 3298, 3421; HRMS (ESI) *m/z*: [M+Na]$^+$ Calcd for $C_{16}H_{16}BrNNaO_4^+$ 388.0160; Found 388.0614.

Methyl 8-(dimethylamino)-1,4-epoxynaphthalene-1(4*H*)-carboxylate (**4j**)

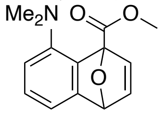

Yield: 21% (10.1 mg, 41.2 μmol); Yellow solid; Mp 59–61 °C; TLC R_f 0.58 (*n*-hexane/EtOAc = 6/1); ^{1}H NMR ($CDCl_3$, 400 MHz): δ 7.30 (d, 1H, *J* = 5.5 Hz), 7.09 (dd, 1H, *J* = 5.5, 1.9 Hz), 7.04–6.95 (m, 2H), 6.76 (dd, 1H, *J* = 7.7, 1.3 Hz), 5.75 (d, 1H, *J* = 1.9 Hz), 3.91 (s, 3H), 2.59 (s, 6H); ^{13}C{^{1}H} NMR ($CDCl_3$, 101 MHz): δ 168.3, 149.3, 147.7, 143.9, 141.6, 140.5, 126.9, 116.9, 115.8, 90.5, 83.2, 52.6, 45.2; IR (NaCl, cm^{-1}) 1019, 1120, 1480, 1743, 2923, 2955, 3417, 3428; HRMS (ESI) *m/z*: [M+Na]$^+$ Calcd for $C_{14}H_{15}NNaO_3^+$ 268.0950; Found 268.0945.

The regiochemistry of **4j** was determined by the NOESY experiments.

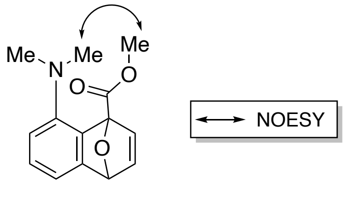

4-Butyl-*N*,*N*-dimethyl-1,4-dihydro-1,4-epoxynaphthalen-5-amine (**4k**)

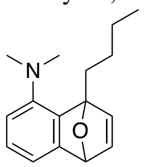

Yield: 43% (20.9 mg, 85.9 μmol); Brown oil; TLC R_f 0.75 (*n*-hexane/EtOAc = 6/1); ^{1}H NMR ($CDCl_3$, 400 MHz): δ 7.04 (dd, 1H, *J* = 5.4, 1.7 Hz), 6.96–6.88 (m, 3H), 6.76–6.70 (m, 1H), 5.58 (d, 1H, *J* = 1.7 Hz), 2.64 (s, 6H), 2.58–2.36 (m, 2H), 1.64–1.38 (m, 4H), 0.95 (t, 3H, *J* = 7.2 Hz); ^{13}C{^{1}H} NMR ($CDCl_3$, 101 MHz): δ 152.1, 148.1, 144.8, 144.3, 141.8, 125.9, 116.2, 114.9, 94.4, 81.2, 45.0, 29.7, 27.4, 23.2, 14.0; IR (NaCl, cm^{-1}) 897, 1316, 1480, 1598, 2827, 2860, 2933, 2956; HRMS (ESI) *m/z*: [M+H]$^+$ Calcd for $C_{16}H_{22}NO^+$ 244.1701; Found 244.1696.

Methyl 8-((4aS^*,8aR^*)-octahydroisoquinolin-2(1*H*)-yl)-1,4-epoxynaphthalene-1(4*H*)-carboxylate (**4l**)

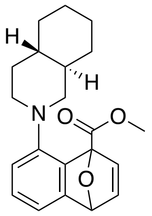

The product was obtained as an inseparable mixture of diastereomers (1:1). The ratio was judged from ^{1}H NMR ($CDCl_3$). Yield: 22% (22.6 mg, 66.6 μmol); Colorless solid; Mp 81–85 °C; TLC R_f 0.50 (*n*-hexane/EtOAc = 6/1); ^{1}H NMR ($CDCl_3$, 400 MHz): δ 7.30 (d, 1H, *J* = 5.5 Hz), 7.07 (dd, 1H, *J* = 5.5, 1.9 Hz), 7.03–6.94 (m, 2H), 6.76 (d, 1H, *J* = 7.8 Hz), 5.75 (d, 1H, *J* = 1.9 Hz), 3,93 (s, 3H), 3.09–2.63 (m, 2.5H), 2.49–2.38 (m, 1H), 2.09 (t, 0.5H, *J* = 11 Hz), 1.82–1.15 (m, 8.5H), 1.10–0.82 (m, 3.5H); ^{13}C{^{1}H} NMR ($CDCl_3$, 101 MHz): δ 168.03, 168.00, 149.2 (two signals overlapped), 147.52, 147.46, 143.8 (two signals overlapped), 141.8 (two signals overlapped), 140.55, 140.51, 126.90, 126.87, 117.3, 117.2, 115.83, 115.80, 90.40, 90.38, 83.22, 83.20, 63.0, 57.2, 56.8, 52.71, 52.65, 50.9, 42.5, 41.4, 41.3, 41.2, 33.5, 33.01, 32.99, 32.6, 30.5, 30.1, 26.42, 26.38, 26.0, 25.9; IR (NaCl, cm^{-1}) 1097, 1203, 1346, 1445, 1747, 1754, 2850, 2922; HRMS (ESI) *m/z*: [M+H]$^+$ Calcd for $C_{21}H_{26}NO_3^+$ 340.1913; Found 340.1905.

Methyl 5-(octahydroisoquinolin-2(1*H*)-yl)-1,4-epoxynaphthalene-1(4*H*)-carboxylate (**5l**)

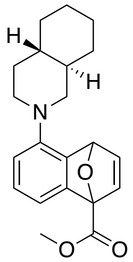

The product was obtained as an inseparable mixture of diastereomers (1:1). The ratio was judged from ^{1}H NMR ($CDCl_3$). Yield: 6% (5.7 mg, 17 μmol); Colorless solid; Mp 103–104 °C; TLC R_f 0.37 (*n*-hexane/EtOAc = 6/1); ^{1}H NMR ($CDCl_3$, 400 MHz): δ 7.14–7.09 (m, 2H), 6.99–6.90 (m, 2H), 6.66–6.61 (m, 1H), 5.98–5.94 (m, 1H), 3,96 (s, 3H), 3.42–3.33 (m, 0.5H), 3.24–3.13 (m, 1H), 3.04–2.96 (m, 0.5H), 2.86–2.67 (m, 1H), 2.49–2.33 (m, 1H), 1.85–1.22 (m, 9H + H_2O), 1.14–0.93 (m, 3H); ^{13}C{^{1}H} NMR ($CDCl_3$, 101 MHz): δ 168.4 (two signals overlapped), 148.2 (two signals overlapped), 146.9 (two signals overlapped), 142.8 (two signals overlapped), 142.7, 142.6, 137.6, 137.5, 126.3 (two signals overlapped), 116.22, 116.16, 113.2 (two signals overlapped), 90.4 (two signals overlapped), 81.70, 81.66, 59.8, 57.2, 53.9, 52.7 (two signals overlapped), 51.3, 42.1, 42.0, 41.64, 41.57, 33.3, 33.1, 32.92, 32.86, 30.4, 30.3, 26.4 (two signals overlapped), 26.0, 25.9; IR (NaCl, cm^{-1}) 1445, 1480, 1743, 1746, 1766, 2850, 2916, 2920, 2923; HRMS (ESI) *m/z*: [M+H]$^+$ Calcd for $C_{21}H_{26}NO_3{}^+$ 340.1907; Found 340.1908.

2-(4-Butyl-1,4-dihydro-1,4-epoxynaphthalen-5-yl)decahydroisoquinoline (**4m**)

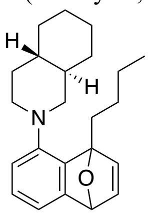

The product was obtained as an inseparable mixture of diastereomers (1:1). The ratio was judged from ^{1}H NMR ($CDCl_3$). Yield: 47% (47.9 mg, 0.142 mmol); Colorless oil; TLC R_f 0.47 (*n*-hexane/EtOAc = 15/1); ^{1}H NMR ($CDCl_3$, 400 MHz): δ 7.03 (dd, 1H, J = 5.4, 1.6 Hz), 6.96–6.89 (m, 2H), 6.87 (dd, 1H, J = 5.3, 5.3 Hz), 6.76–6.70 (m, 1H), 5.57 (d, 1H, J = 1.1 Hz), 3.18–2.60 (m, 2.5H), 2.56–2.43 (m, 2H), 2.41–2.30 (m, 1H), 2.17 (t, 0.5H, J = 11 Hz), 1.87–1.18 (m, 13H), 1.15–0.90 (m, 6H); ^{13}C{^{1}H} NMR ($CDCl_3$, 101 MHz): δ 152.1 (two signals overlapped), 147.9, 147.9, 144.7, 144.7, 144.6 (two signals overlapped), 142.1 (two signals overlapped), 125.9, 125.9, 116.9, 116.9, 115.2, 115.1, 94.3 (two signals overlapped), 81.2 (two signals overlapped), 63.1 (two signals overlapped), 57.1, 56.9, 50.9 (two signals overlapped), 42.6, 42.3, 41.6, 41.4, 33.6, 33.4, 33.1, 33.0, 30.6, 30.3, 30.0, 29.8, 27.3, 27.3, 26.4, 26.4, 26.0, 23.4, 14.2, 14.2; IR (NaCl, cm^{-1}) 844, 1143, 1212, 1418, 1464, 2855, 2926, 2952; HRMS (ESI) *m/z*: [M+H]$^+$ Calcd for $C_{23}H_{32}NO^+$ 338.2484; Found 338.2472.

2-(1-Butyl-1,4-dihydro-1,4-epoxynaphthalen-5-yl)decahydroisoquinoline (**5m**)

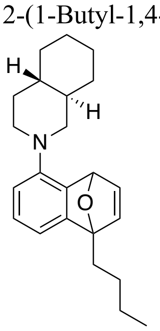

The product was obtained as an inseparable mixture of diastereomers (1:1). The ratio was judged from ^{1}H NMR ($CDCl_3$). Yield: 8% (8.5 mg, 25 μmol); Yellow oil; TLC R_f 0.33 (*n*-hexane/EtOAc = 6/1); ^{1}H NMR ($CDCl_3$, 400 MHz): δ 7.10–7.04 (m, 1H), 6.91 (dd, 1H, J = 7.6, 7.6 Hz), 6.83–6.76 (m, 2H), 6.58 (dd, 1H, J = 7.6, 1.5 Hz), 5.80 (dd, 1H, J = 2.0, 2.0 Hz), 3.41–2.98 (m, 2H), 2.85–2.64 (m, 1H), 2.48–2.16 (m, 3H), 1.82–1.61 (m, 5H), 1.51–1.23 (m, 8H), 1.13–0.92 (m, 6H); ^{13}C{^{1}H} NMR ($CDCl_3$, 101 MHz): δ 151.7 (two signals overlapped), 146.3 (two signals overlapped), 145.1, 145.1, 143.5 (two signals overlapped), 140.9, 140.8, 125.9 (two signals overlapped), 115.2, 115.1, 112.8 (two signals overlapped), 92.63, 92.62, 80.8, 80.7, 59.9, 57.1, 54.0, 51.2, 42.2, 42.0, 41.74, 41.67, 33.4, 33.2, 33.0, 32.9, 30.5, 30.3, 29.7 (two signals overlapped), 29.1(two signals overlapped), 26.8, 26.4, 26.02, 25.99, 23.2 (two signals overlapped), 14.0 (two signals overlapped); IR (NaCl, cm^{-1}) 1030, 1265, 1464, 1621, 2855, 2928, 3380, 3410; HRMS (ESI) *m/z*: [M+H]$^+$ Calcd for $C_{23}H_{32}NO^+$ 338.2484; Found 338.2473.

Methyl 6-methyl-8-morpholino-1,4-epoxynaphthalene-1(4*H*)-carboxylate (**4n**)

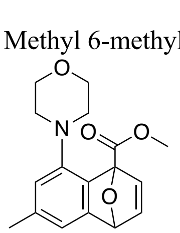

Yield: 47% (14.0 mg, 46.5 μmol); Colorless oil; TLC R_f 0.20 (*n*-hexane/EtOAc = 6/1); ^{1}H NMR ($CDCl_3$, 400 MHz): δ 7.30–7.25 (m, 1H), 7.08 (dd, 1H, J = 5.5, 1.9 Hz), 6.93 (s, 1H), 6.57 (s, 1H), 5.74 (d, 1H, J = 1.9 Hz), 3.98 (s, 3H), 3.90–3.82 (AA'BB', 2H), 3.76–3.68 (AA'BB', 2H), 2.95–2.87 (AA'BB', 2H), 2.80–2.72 (AA'BB', 2H), 2.31 (s, 3H); ^{13}C{^{1}H} NMR ($CDCl_3$, 101 MHz): δ 168.3, 149.7, 146.0, 143.7, 140.6, 138.9, 137.4, 117.8, 117.0, 90.2, 83.1, 66.9, 52.9, 52.7, 21.3; IR (NaCl, cm^{-1}) 844, 939, 1115, 1640, 1743, 3403, 3446, 3478; HRMS (ESI) *m/z*: [M+Na]$^+$ Calcd for $C_{17}H_{19}NNaO_4^+$ 324.1212; Found 324.1211.

The regiochemistry of **4n** was determined by the NOESY experiments.

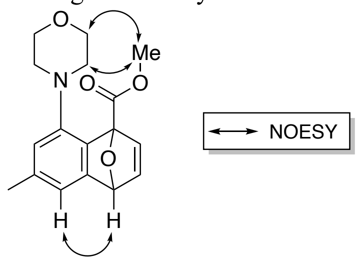

Methyl 7-methyl-5-morpholino-1,4-epoxynaphthalene-1(4*H*)-carboxylate (**5n**)

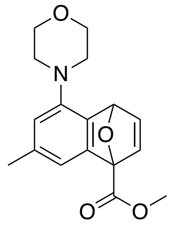

Yield: 12% (3.8 mg, 13 μmol); Colorless oil; TLC R_f 0.47 (*n*-hexane/EtOAc = 1/1); ^{1}H NMR ($CDCl_3$, 400 MHz): δ 7.11–7.07 (m, 2H), 6.88 (s, 1H), 6.44 (s, 1H), 5.94 (s, 1H), 3.97 (s, 3H), 3.93–3.80 (AA'BB', 4H), 3.07–2.93 (AA'BB', 4H), 2.28 (s, 3H); ^{13}C{^{1}H} NMR ($CDCl_3$, 101 MHz): δ 168.3, 148.8, 145.8, 142.9, 142.5, 136.8, 134.9, 116.1, 115.4, 90.2, 81.4, 67.0, 52.8, 51.8, 21.4; IR (NaCl, cm^{-1}) 1119, 1259, 1451, 1739, 1743, 2855, 2923, 2956; HRMS (ESI) *m/z*: [M+Na]$^+$ Calcd for $C_{17}H_{19}NNaO_4^+$ 324.1212; Found 324.1213.

4-(4-Butyl-7-methyl-1,4-dihydro-1,4-epoxynaphthalen-5-yl)morpholine (**4o**)

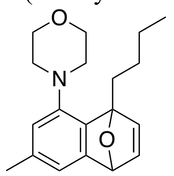

Yield: 63% (18.7 mg, 62.5 μmol); Colorless oil; TLC R_f 0.60 (*n*-hexane/EtOAc = 6/1); ^{1}H NMR ($CDCl_3$, 400 MHz): δ 7.01 (dd, 1H, J = 5.4, 1.8 Hz), 6.86–6.79 (m, 2H), 6.53 (s, 1H), 5.54 (d, 1H, J = 1.8 Hz), 3.93–3.85 (m, 2H), 3.82–3.75 (m, 2H), 2.92–2.77 (AA'BB', 4H), 2.52–2.25 (m, 2H+3H), 1.69–1.40 (m, 4H), 0.99–0.92 (m, 3H); ^{13}C{^{1}H} NMR ($CDCl_3$, 101 MHz): δ 152.8, 146.4, 144.7, 144.6, 139.4, 136.3, 117.4, 116.9, 94.0, 81.1, 67.2, 53.2 (br), 30.2, 27.3, 23.4, 21.3, 14.2 ; IR (NaCl, cm^{-1}) 843, 874, 1451, 1455, 1471, 1593, 2955; HRMS (ESI) *m/z*: [M+Na]$^+$ Calcd for $C_{19}H_{25}NNaO_2^+$ 322.1783; Found 322.1783.

The regiochesmistry of **4o** was determined by the NOESY experiments.

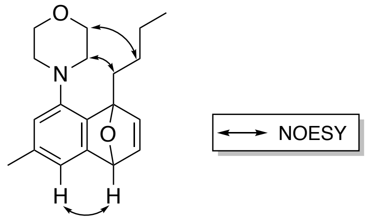

4-(1-Butyl-7-methyl-1,4-dihydro-1,4-epoxynaphthalen-5-yl)morpholine (**5o**)

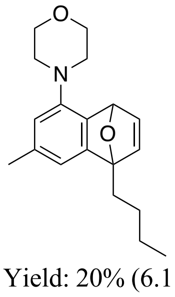

Yield: 20% (6.1 mg, 3.0 μmol); Colorless solid; Mp 105–108 °C; TLC R_f 0.25 (*n*-hexane/EtOAc = 6/1); ^{1}H NMR ($CDCl_3$, 400 MHz): δ 7.04 (dd, 1H, J = 5.4, 1.5 Hz), 6.80 (d, 1H, J = 5.4 Hz), 6.69 (s, 1H), 6.38 (s, 1H), 5.78 (d, 1H, J = 1.5Hz), 3.93–3.77 (AA'BB', 4H), 3.05–2.94 (AA'BB', 4H), 2.36–2.14 (m, 5H), 1.52–1.38 (m, 4H), 0.96

(t, 3H, J = 1.6Hz); $^{13}C\{^1H\}$ NMR ($CDCl_3$, 101 MHz): δ 152.3, 145.2, 145.0, 143.5, 138.1, 136.2, 115.13, 115.09, 92.5, 80.5, 67.1, 51.8, 29.0, 26.8, 23.2, 21.5, 14.0; IR (NaCl, cm^{-1}) 876, 1119, 1233, 1451, 1468, 1600, 2857, 2932, 2956; HRMS (ESI) *m/z*: $[M+Na]^+$ Calcd for $C_{19}H_{25}NNaO_2^+$ 322.1783; Found 322.1780.

Methyl 6-bromo-8-morpholino-1,4-epoxynaphthalene-1(4*H*)-carboxylate (**4p**)

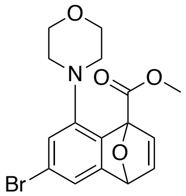

Yield: 46% (8.8 mg, 20 μmol); Collorless solid; Mp 159–163 °C; TLC R_f 0.26 (*n*-hexane/EtOAc = 6/1); ^1H NMR ($CDCl_3$, 400 MHz): δ 7.30–7.25 (m, 1H), 7.22 (d, 1H, J = 1.4 Hz), 7.09 (dd, 1H, J = 5.5, 1.9 Hz), 6.90 (d, 1H, J = 1.4 Hz), 5.75 (d, 1H, J = 1.9 Hz), 3.98 (s, 3H), 3.88–3.81 (AA'BB', 2H), 3.75–3.67 (AA'BB', 2H), 2.92–2.84 (AA'BB', 2H), 2.79–2.71 (AA'BB', 2H); $^{13}C\{^1H\}$ NMR ($CDCl_3$, 101 MHz): δ 167.7, 151.6, 147.0, 143.6, 141.2, 140.5, 120.3, 120.04, 120.02, 90.2, 82.8, 66.7, 52.9, 52.7; IR (NaCl, cm^{-1}) 1099, 1116, 1202, 1222, 1447, 1588, 1743, 1747, 2856, 2955; HRMS (ESI) *m/z*: $[M+Na]^+$ Calcd for $C_{16}H_{16}BrNNaO_4^+$ 388.0160; Found 388.0160.

Methyl 7-bromo-5-morpholino-1,4-epoxynaphthalene-1(4*H*)-carboxylate (**5p**)

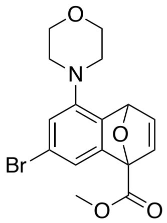

Yield: 24% (4.5 mg, 12 μmol); Yellow solid; Mp 158–162 °C; TLC R_f 0.13 (*n*-hexane/EtOAc = 6/1); ^1H NMR ($CDCl_3$, 400 MHz): δ 7.20–7.17 (m, 1H), 7.12–7.07 (m, 2H), 6.76 (d, 1H, J = 1.4 Hz), 5.93–5.90 (m, 1H), 3.98 (s, 3H), 3.90–3.78 (AA'BB', 4H), 3.04–2.91 (AA'BB', 4H); $^{13}C\{^1H\}$ NMR ($CDCl_3$, 101 MHz): δ 167.6, 150.5, 146.8, 142.8, 142.6, 137.2, 119.8, 118.7, 117.7, 90.0, 81.3, 66.7, 53.0, 51.4; IR (NaCl, cm^{-1}) 1053, 1067, 1086, 1116, 1445, 1588, 2852, 2919; HRMS (ESI) *m/z*: $[M+Na]^+$ Calcd for $C_{16}H_{16}BrNNaO_4^+$ 388.0160; Found 388.0157.

4-(7-Bromo-4-butyl-1,4-dihydro-1,4-epoxynaphthalen-5-yl)morpholine (**4q**)

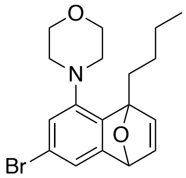

Yield: 44% (7.9 mg, 22 μmol); Yellow oil; TLC R_f 0.57 (*n*-hexane/EtOAc = 6/1); ^1H NMR ($CDCl_3$, 400 MHz): δ 7.12 (d, 1H, J = 1.4 Hz), 7.00 (dd, 1H, J = 5.5, 1.8 Hz), 6.86 (d, 1H, J = 1.4 Hz), 6.82 (d, 1H, J = 5.5 Hz), 5.55 (d, 1H, J = 1.8 Hz), 3.93–3.85 (m, 2H), 3.83–3.75 (m, 2H), 2.88–2.77 (m, 4H), 2.48–2.28 (m, 2H), 1.40–1.58 (m, 4H), 0.96 (t, 3H, J = 7.1 Hz); $^{13}C\{^1H\}$ NMR ($CDCl_3$, 101 MHz): δ 154.7, 147.5, 144.55, 144.48, 142.0, 119.9, 119.6, 119.2, 94.1, 80.9, 67.0, 52.9 (br), 29.9, 27.2, 23.3, 14.2; IR (NaCl, cm^{-1}) 893, 1118, 1225, 1263, 1445, 1588, 2856, 2930, 2956; HRMS (APCI) *m/z*: $[M+H]^+$ Calcd for $C_{18}H_{23}BrNO_2^+$ 364.0912; Found 364.0913.

4-(7-Bromo-1-butyl-1,4-dihydro-1,4-epoxynaphthalen-5-yl)morpholine (**5q**)

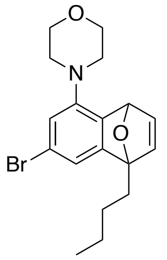

Yield: 29% (5.3 mg, 15 μmol); Yellow oil; TLC R_f 0.33 (*n*-hexane/EtOAc = 6/1); ^1H NMR ($CDCl_3$, 400 MHz): δ 7.04 (dd, 1H, J = 5.4, 1.6 Hz), 6.97 (d, 1H, J = 1.4 Hz), 6.80 (d, 1H, J = 5.4 Hz), 6.71 (d, 1H, J = 1.4 Hz), 5.76 (d, 1H, J = 1.6 Hz), 3.93–3.77 (AA'BB', 4H), 3.30–2.92 (AA'BB', 4H), 2.33–2.13 (m, 2H), 1.56–1.40 (m, 4H), 0.96

(t, 3H, J = 7.2 Hz); $^{13}C\{^1H\}$ NMR ($CDCl_3$, 101 MHz): δ 154.4, 146.2, 145.0, 143.4, 140.4, 119.5, 117.7, 117.3, 92.6, 80.5, 66.9, 51.5, 28.8, 26.7, 23.1, 14.0; IR (NaCl, cm^{-1}) 966, 1120, 1218, 1428, 1445, 1585, 1593, 2959; HRMS (APCI) *m/z*: $[M+H]^+$ Calcd for$C_{18}H_{23}BrNO_2{}^+$ 364.0912; Found 364.0913.

1-Butyl-8-methoxy-1,4-dihydro-1,4-epoxynaphthalene (**9b**)
1-Butyl-5-methoxy-1,4-dihydro-1,4-epoxynaphthalene (**10b**)

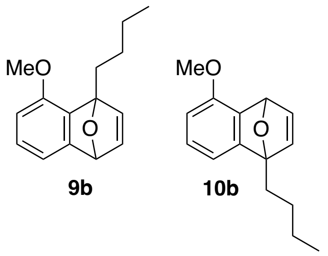

An inseparable mixture of **9b** and **10b** (71:29) was obtained.; Yield: 98% (45.6 mg, 0.198 mmol);; Colorless oil; TLC R_f 0.57 (n-hexane/CH_2Cl_2 = 6/1); ^1H NMR ($CDCl_3$, 400 MHz) for **9b**: δ 7.00 (dd, 1H, J = 5.4, 1.8 Hz), 6.96 (dd, 1H, J = 8.3, 8.3 Hz), 6.88 (d, 1H, J = 8.3 Hz), 6.86 (d, 1H, J = 5.4 Hz), 6.60 (d, 1H, J = 8.3 Hz), 5.62 (d, 1H, J = 1.8 Hz), 3.80 (s, 3H), 2.56–2.37 (m, 2H), 1.68–1.42 (m, 4H), 0.98 (t, 3H, J = 7.2 Hz); for **10b**: δ 7.07 (dd, 1H, J = 5.4, 1.8 Hz), 6.99–6.95 (m, 1H), 6.84 (d, 1H, J = 8.3 Hz), 6.80 (d, 1H, J = 5.4 Hz), 6.59 (d, 1H, J = 8.3 Hz), 5.91 (d, 1H, J = 1.8 Hz), 3.83 (s, 3H), 2.37–2.20 (m, 2H), 1.68–1.42 (m, 4H), 0.98 (t, 3H, J = 7.3 Hz); $^{13}C\{^1H\}$ NMR ($CDCl_3$, 101 MHz) for **9b**: δ 153.8, 153.4, 145.4, 143.9, 135.7, 126.9, 113.3, 110.1(signal overlapped with distal's one), 94.0, 81.7, 55.3, 30.3, 27.2, 23.1, 14.0; for **10b**: δ 153.2, 152.6, 144.6, 144.2, 136.9, 126.7, 112.7, 110.1(signal overlapped with proximal's one), 93.1, 79.3, 55.7, 29.0, 26.8, 23.1, 14.0; IR (NaCl, cm^{-1}) 1050, 1262, 1471, 1480, 1600, 1607, 2935, 2956; HRMS (ESI) *m/z*: $[M+H]^{\cdot+}$ Calcd for $C_{15}H_{19}O_2{}^{\cdot+}$ 231.1380; Found 231.1379.

The regiochemistries of **9b** and **10b** were determined by the HMBC and HSQC experiments.

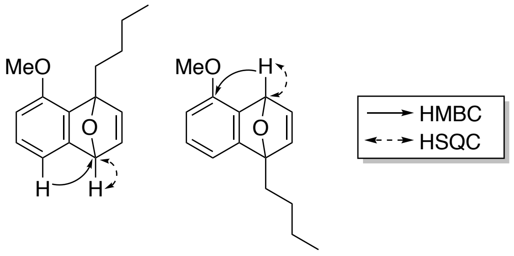

4-(5,6,7,8-Tetraphenylnaphthalen-1-yl)morpholine (**7a**)

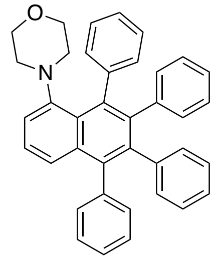

Yield: 51% (53.2 mg, 0.103 mmol); Colorless solid; Mp 183–185 °C; TLC R_f 0.67 (n-hexane/EtOAc = 6/1); ^1H NMR ($CDCl_3$, 400 MHz): 7.48–7.30 (m, 3H), 7.25–7.14 (m, 5H), 7.08–7.03 (m, 5H), 6.88–6.74 (m, 8H), 6.71–6.65 (m, 2H), 3.51–3.41 (m, 2H), 2.81–2.56 (m, 6H); $^{13}C\{^1H\}$ NMR ($CDCl_3$, 101 MHz): δ 151.0, 143.1, 140.7, 140.6, 140.5, 140.1, 139.0, 138.8, 136.2, 134.7, 131.5, 131.2, 131.0, 130.4, 127.42, 127.37, 126.4, 126.3, 126.3, 126.2, 125.8, 125.1, 124.9, 124.8, 124.2, 119.9, 66.0, 52.5; IR (NaCl, cm^{-1}) 1115, 1442, 1654, 2815, 3058, 3401, 3421, 3463; HRMS (ESI) *m/z*: $[M+Na]^+$ Calcd for $C_{38}H_{31}NNaO^+$ 540.2303; Found 540.2299.

4-Methoxy-5-morpholinonaphthalen-1-ol (**7b**)

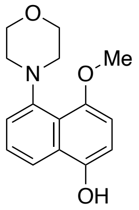

Yield: 77% (39.8 mg, 0.154 mmol); Colorless solid; Mp 154–158 °C; TLC R_f 0.16 (*n*-hexane/EtOAc = 6/1); ^{1}H NMR ($CDCl_3$, 400 MHz): δ 7.93 (dd, 1H, *J* = 8.0, 1.1 Hz), 7.41 (dd, 1H, *J* = 8.0, 8.0 Hz), 7.07 (dd, 1H, *J* = 8.0, 1.1 Hz), 6.79–6.72 (m, 2H), 4.03–3.98 (m, 4H), 3.89 (s, 3H), 3.36–3.24 (br, 2H), 3.00–2.86 (m, 2H); ^{13}C{^{1}H} NMR ($CDCl_3$, 101 MHz): δ 150.1, 149.1, 146.5, 128.0, 125.6, 121.3, 117.0, 114.8, 109.0, 108.4, 67.4, 58.0, 54.2; IR (NaCl, cm^{-1}) 959, 1369, 1385, 1588, 2810, 2991, 3185, 3212; HRMS (ESI) *m/z*: [M+H]$^+$ Calcd for $C_{15}H_{18}NO_3^+$ 260.1287; Found 260.1287.

Methyl 8-morpholino-1-naphthoate (**7c**)

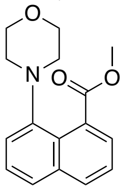

Yield: 61% (17.0 mg, 63.0 μmol); Colorless solid; Mp 137–139 °C; TLC R_f 0.25 (*n*-hexane/EtOAc = 6/1); ^{1}H NMR ($CDCl_3$, 400 MHz): δ 7.89 (dd, 1H, *J* = 7.5, 1.9 Hz), 7.70 (dd, 1H, *J* = 8.1, 1.0 Hz), 7.55–7.42 (m, 4H), 4.00 (s, 3H), 3.94–3.75 (AA'BB', 4H), 3.05–2.83 (AA'BB', 4H); ^{13}C{^{1}H} NMR ($CDCl_3$, 101 MHz): δ 172.0, 149.3, 135.1, 130.4, 130.0, 127.1, 126.7, 125.9, 125.7, 125.1, 119.9, 66.8, 53.7, 52.4; IR (NaCl, cm^{-1}) 828, 1004, 1115, 1198, 1235, 1276, 1724, 2925; HRMS (ESI) *m/z*: [M+Na]$^+$ Calcd for $C_{16}H_{17}NNaO_3^+$ 294.1106; Found 294.1108.

Methyl 1-hydroxy-8-morpholino-2-naphthoate (**7d**)

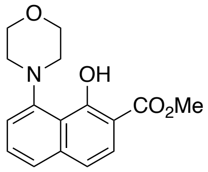

Yield: 19% (5.4 mg, 19 μmol); Colorless solid; Mp 162–164 °C; TLC R_f 0.16 (*n*-hexane/EtOAc = 6/1); ^{1}H NMR ($CDCl_3$, 400 MHz): δ 15.84 (br s, 1H), 7.86 (d, 1H, *J* = 8.8 Hz), 7.67 (br d, 1H, *J* = 7.8 Hz), 7.51 (dd, 1H, *J* = 7.8, 7.8 Hz), 7.39 (br d, 1H, *J* = 7.8 Hz), 7.23 (d, 1H, *J* = 8.8 Hz), 4.08–3.92 (m, 4H + 3H), 3.27–3.06 (m, 4H); ^{13}C{^{1}H} NMR ($CDCl_3$, 101 MHz): δ 167.1, 160.1, 149.2, 138.2, 128.2, 128.0, 127.2, 119.1, 118.9, 117.6, 110.8, 66.8, 54.1, 51.9; IR (NaCl, cm^{-1}) 1012, 1118, 1259, 1621, 1723, 2855, 2926, 2956, 3055; HRMS (ESI) *m/z*: [M+Na]$^+$ Calcd for $C_{16}H_{17}NNaO_4^+$ 310.1055; Found 310.1055.

Methyl 8-morpholino-3-(*p*-tolyl)-1-naphthoate (**7e**)

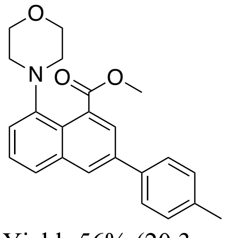

Yield: 56% (20.3 mg, 56.2 μmol); Colorless solid; Mp 147–150 °C; TLC R_f 0.37 (*n*-hexane/EtOAc = 3/1); ^{1}H NMR ($CDCl_3$, 400 MHz): δ 8.06 (d, 1H, *J* = 1.8 Hz), 7.76–7.72 (m, 1H), 7.70 (d, 1H, *J* = 1.8 Hz), 7.64–7.58 (AA'BB', 2H), 7.53 (dd, 1H, *J* = 7.8, 7.8 Hz), 7.42 (dd, 1H, *J* = 7.8, 0.9 Hz), 7.32–7.27 (AA'BB', 2H), 4.03 (s, 3H), 3.96–3.76 (AA'BB', 4H), 3.07–2.85 (AA'BB', 4H), 2.42 (s, 3H); ^{13}C{^{1}H} NMR ($CDCl_3$, 101 MHz): δ 171.9, 149.3, 137.9, 137.7, 136.9, 135.5, 131.0, 129.7, 127.2, 127.09, 127.06, 126.1, 125.9, 125.6, 119.6, 66.8, 53.7 (br), 52.4, 21.1; IR (NaCl, cm^{-1}) 1115, 1192, 1235, 1249, 1727, 2862, 2925, 2950; HRMS (ESI) *m/z*: [M+H]$^+$ Calcd for $C_{23}H_{24}NO_3{}^+$ 362.1751; Found 362.1751.

4-(5,8-Diphenylnaphthalen-1-yl)morpholine (**7f**)

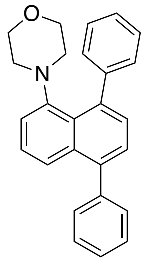

Yield: 36% (13.2 mg, 36.1 μmol); Yellow solid; Mp 139–143 °C; TLC R_f 0.53 (*n*-hexane/EtOAc = 1/1); ^{1}H NMR ($CDCl_3$, 400 MHz): δ 7.70 (dd, 1H, *J* = 8.4, 1.1 Hz), 7.53–7.33 (m, 13H), 7.18 (dd, 1H, *J* = 8.4, 1.1 Hz), 3.59–3.38 (br, 2H), 2.82–2.56 (m, 6H); ^{13}C{^{1}H} NMR ($CDCl_3$, 101 MHz): δ 150.2, 144.6, 141.2, 140.3, 138.5, 134.6, 130.2, 129.8, 129.3, 128.2, 127.2, 126.9, 126.3, 126.2, 126.1, 125.8, 122.6, 116.1, 65.9, 52.4; IR (NaCl, cm^{-1}) 947, 1115, 1232, 1408, 3415, 3433; HRMS (ESI) *m/z*: [M+H]$^+$ Calcd for$C_{26}H_{24}NO^+$ 366.18579; Found 366.18571.

4-(9,10-Diphenylanthracen-1-yl)morpholine (**7h**)

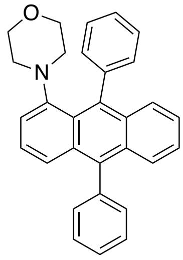

Yield: 65% (62.1 mg, 0.149 mmol); Yellow solid; Mp 250–252 °C; TLC R_f 0.67 (*n*-hexane/EtOAc = 6/1); ^{1}H NMR ($CDCl_3$, 400 MHz): δ 7.63–7.38 (m, 13H), 7.32–7.27 (m, 4H), 3.56–3.47 (AA'BB', 2H), 2.80–2.70 (m, 4H), 2.62–2.56 (AA'BB', 2H); ^{13}C{^{1}H} NMR ($CDCl_3$, 101 MHz): δ 151.1, 143.4, 139.6, 137.8, 135.2, 132.1, 131.2, 131.2, 130.0, 129.9, 129.8, 128.4, 127.5, 127.4, 127.1, 126.8, 126.3, 125.9, 124.9, 124.9, 124.7, 119.4, 66.2, 52.6; IR (NaCl, cm^{-1}) 1112, 2362, 3649, 3733, 3803, 3852, 3866; HRMS (ESI) *m/z*: [M+H]$^+$ Calcd for $C_{30}H_{26}NO^+$ 416.2014; Found 416.2013.

4-(8-Butylnaphthalen-1-yl)morpholine (**7i**)

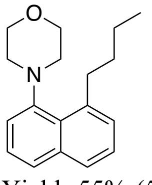

Yield: 55% (5.5 mg, 20 μmol); Yellow solid; Mp 93–96 °C; TLC R_f 0.67 (*n*-hexane/EtOAc = 6/1); ^{1}H NMR ($CDCl_3$, 400 MHz): δ 7.68 (dd, 1H, *J* = 8.0, 1.4 Hz), 7.62 (dd, 1H, *J* = 8.0, 1.4 Hz), 7.40 (dd, 1H, *J* = 8.0, 8.0 Hz), 7.36–7.26 (m, 3H), 3.99–3.89 (AA'BB', 4H), 3.49–3.41 (m, 2H), 3.06–2.95 (AA'BB', 4H), 1.57–1.49 (m, 2H), 1.44–1.33 (m, 2H), 0.90 (t, 3H, *J* = 7.3 Hz); ^{13}C{^{1}H} NMR ($CDCl_3$, 101 MHz): δ 150.9, 139.7, 136.6, 129.0, 127.7, 127.5, 126.1, 125.4, 125.1, 117.6, 67.0, 54.3, 36.7, 35.1, 23.0, 14.3; IR (NaCl, cm^{-1}) 1030, 1118, 1259, 1450, 2822, 2855, 2929, 2956; HRMS (ESI) *m/z*: [M+H]$^+$ Calcd for $C_{18}H_{24}NO^+$ 270.1858; Found 270.1853.

4-(5-Butylnaphthalen-1-yl)morpholine (**7j**)

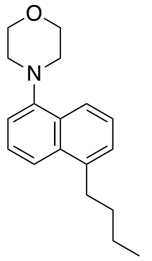

Yield: 46% (13.1 mg, 48.6 μmol); Yellow solid; Mp 56–59 °C; TLC R_f 0.67 (*n*-hexane/EtOAc = 6/1); ^{1}H NMR ($CDCl_3$, 400 MHz): δ 8.13 (d, 1H, *J* = 8.4 Hz), 7.79 (d, 1H, *J* = 8.4 Hz), 7.47–7.37 (m, 2H), 7.31 (d, 1H, *J* = 6.4 Hz), 7.13–7.08 (m, 1H), 4.02–3.95 (AA'BB', 4H), 3.17–3.02 (m, 4H + 2H), 1.78−1.67 (m, 2H), 1.50–1.40 (m, 2H), 0.97 (t, 3H, *J* = 7.3 Hz); ^{13}C{^{1}H} NMR ($CDCl_3$, 101 MHz): δ 150.0, 139.5, 133.2, 129.3, 126.0, 125.5, 125.1, 121.7, 119.8, 114.4, 67.5, 53.6, 33.2, 33.1, 22.9, 14.0; IR (NaCl, cm^{-1}) 1118, 1261, 1409, 1451, 2822, 2855, 2893,

2929, 29561375, 1437, 1465, 1508, 1541, 1647, 1684, 1700, 1717; HRMS (ESI) *m/z*: $[M+H]^+$ Calcd for $C_{18}H_{24}NO^+$ 270.1852; Found 270.1852.

Methyl 6-methyl-8-morpholino-1-naphthoate (**7l**)

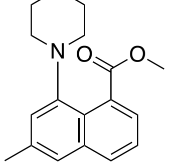

Yield: 53% (10.2 mg, 35.7 μmol); Colorless solid; Mp 140–142 °C; TLC R_f 0.37 (*n*-hexane/EtOAc = 6/1); ^{1}H NMR ($CDCl_3$, 400 MHz): δ 7.79 (dd, 1H, *J* = 7.5, 1.2 Hz), 7.48 (s, 1H), 7.43 (dd, 1H, *J* = 7.5, 7.5 Hz), 7.37 (dd, 1H, *J* = 7.5, 1.2 Hz), 7.26–7.24 (m, 1H), 3.99 (s, 3H), 3.93–3.86 (AA'BB', 2H), 3.83–3.75 (AA'BB', 2H), 3.08–2.90 (AA'BB', 2H), 2.90–2.82 (AA'BB', 2H), 2.51 (s, 3H); $^{13}C\{^1H\}$ NMR ($CDCl_3$, 101 MHz): δ 172.1, 149.1, 136.5, 135.2, 130.9, 130.2, 129.3, 125.2, 125.0, 124.8, 122.0, 66.8, 53.7, 52.3, 21.7; IR (NaCl, cm^{-1}) 873, 1115, 1139, 1199, 1251, 1276, 1372, 1624, 2952; HRMS (ESI) *m/z*: $[M+Na]^+$ Calcd for $C_{17}H_{19}NNaO_3^+$ 308.1262; Found 308.1263

Methyl 7-methyl-5-morpholino-1-naphthoate (**7m**)

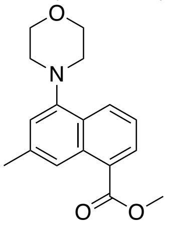

Yield: 50% (1.9 mg, 6.7 μmol); Colorless solid; Mp 99–102 °C; TLC R_f 0.47 (*n*-hexane/EtOAc = 6/1); ^{1}H NMR ($CDCl_3$, 400 MHz): δ 8.42 (d, 1H, *J* = 7.8 Hz), 8.38 (s, 1H), 8.10 (dd, 1H, *J* = 7.8, 1.1 Hz), 7.42 (dd, 1H, *J* = 7.8, 7.8 Hz), 7.00 (s, 1H), 4.01–3.94 (m, 3H + 4H), 3.10–3.04 (br, 4H), 2.53 (s, 3H); $^{13}C\{^1H\}$ NMR ($CDCl_3$, 101 MHz): δ 168.4, 149.6, 137.7, 132.8, 130.1, 128.3, 127.6, 127.0, 123.1, 120.4, 117.5, 67.4, 53.7, 52.1, 22.5; IR (NaCl, cm^{-1}) 1246, 1641, 1714, 2917, 3355, 3414, 3448, 3478; HRMS (ESI) *m/z*: $[M+H]^+$ Calcd for $C_{17}H_{20}NO_3^+$ 286.1443; Found 286.1443.

Methyl 1-hydroxy-5-morpholino-2-naphthoate (**7n**)

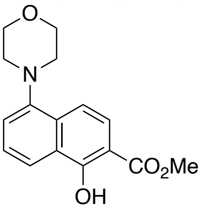

Yield: 36% (5.6 mg, 20 μmol); Colorless solid; Mp 141–142 °C; TLC R_f 0.47 (*n*-hexane/EtOAc = 6/1); ^{1}H NMR ($CDCl_3$, 400 MHz): δ 11.95 (br s, 1H), 8.15 (br d, 1H, *J* = 8.0 Hz), 7.77 (d, 1H, *J* = 9.2 Hz), 7.64 (dd, 1H, *J* = 9.2, 0.8 Hz), 7.47 (dd, 1H, *J* = 8.0, 8.0 Hz), 7.24 (dd, 1H, *J* = 8.0, 0.8 Hz), 4.02–3.94 (m, 3H + 4H), 3.13–3.04 (AA'BB', 4H); $^{13}C\{^1H\}$ NMR ($CDCl_3$, 101 MHz): δ 171.4, 161.2, 149.1, 132.6, 126.0, 125.9, 123.7, 119.2, 118.3, 114.1, 105.6, 67.4, 53.3, 52.3; IR (NaCl, cm^{-1}) 963, 1116, 1259, 1424, 1667, 2822, 2851, 2892, 2852, 2892, 2955, 3066; HRMS (ESI) *m/z*: $[M+Na]^+$ Calcd for $C_{16}H_{17}NNaO_4^+$ 310.1055; Found 310.1060.

Methyl 4-hydroxy-5-morpholino-1-naphthoate (**7o**)

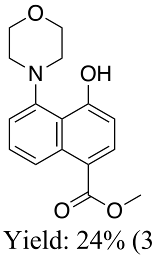

Yield: 24% (3.8 mg, 13 μmol); Colorless solid; Mp 143–147 °C; TLC R_f 0.49 (*n*-hexane/EtOAc = 6/1); ^{1}H NMR ($CDCl_3$, 400 MHz): δ 15.4 (s, 1H), 9.06 (d, 1H, *J* = 8.1 Hz), 8.21 (d, 1H, *J* = 8.3 Hz), 7.56 (dd, 1H, *J* = 8.1, 8.1

Hz), 7.42 (d, 1H, J = 8.1 Hz), 6.83 (d, 1H, J = 8.3 Hz), 4.10–4.03 (AA'BB', 2H), 3.94 (s, 3H), 3.92–3.85 (AA'BB', 2H), 3.28–3.18 (AA'BB', 2H), 3.12–3.04 (AA'BB', 2H); $^{13}C\{^{1}H\}$ NMR ($CDCl_3$, 101 MHz): δ 167.7, 161.8, 147.8, 135.2, 133.9, 127.6, 125.6, 118.8, 118.3, 116.5, 109.4, 67.0, 54.2, 51.8; IR (NaCl, cm^{-1}) 1116, 1245, 1517, 1580, 1694, 1698, 1703, 2853, 2922; HRMS (ESI) *m/z*: $[M+Na]^+$ Calcd for $C_{16}H_{17}NNaO_4^+$ 310.1055; Found 310.1056.

The regiochemistry of **7o** was determined by the NOESY experiments.

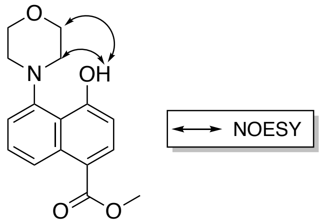

Methyl 5-morpholino-3-(*p*-tolyl)-1-naphthoate (**7p**)

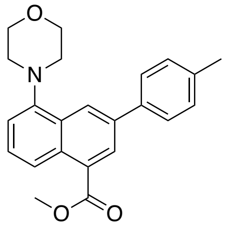

Yield: 67% (24.3 mg, 67.2 μmol); Bege solid; Mp 101–103 °C; TLC R_f 0.43 (*n*-hexane/EtOAc = 6/1); ^{1}H NMR ($CDCl_3$, 400 MHz): δ 8.71–8.68 (m, 1H), 8.58 (d, 1H, J = 8.7 Hz), 8.42 (d, 1H, J = 2.0 Hz), 7.67–7.62 (AA'BB', 2H), 7.53 (dd, 1H, J = 8.7, 8.7 Hz), 7.36–7.31 (AA'BB', 2H), 7.21–7.16 (m, 1H), 4.03 (s, 3H), 4.02–3.96 (AA'BB', 4H), 3.17–3.09 (br, 4H), 2.44 (s, 3H); $^{13}C\{^{1}H\}$ NMR ($CDCl_3$, 101 MHz): δ 168.2, 149.9, 137.6, 137.5, 136.7, 131.6, 129.8, 129.8, 129.5, 128.4, 127.5, 127.1, 125.7, 121.4, 115.7, 67.4, 53.7, 52.3, 21.1; IR (NaCl, cm^{-1}) 1118, 1201, 1236, 1296, 1716, 1721, 2850, 2953; HRMS (ESI) *m/z*: $[M+H]^+$ Calcd for $C_{23}H_{24}NO_3^+$ 362.1751; Found 362.1750.

Methyl 3-(4-((*tert*-butyldimethylsilyl)oxy)phenyl)-5-morpholino-1-naphthoate (**7q**)

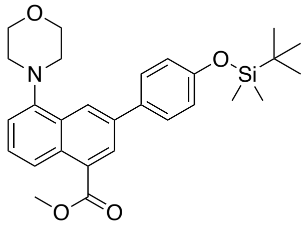

Yield: 56% (13.6 mg, 28.5 μmol); Yellow solid; Mp 128–130 °C; TLC R_f 0.47 (*n*-hexane/EtOAc = 6/1); ^{1}H NMR ($CDCl_3$, 400 MHz): δ 8.66 (d, 1H, J = 1.9 Hz), 8.56 (d, 1H, J = 8.6 Hz), 8.39 (d, 1H, J = 1.9 Hz), 7.65–7.59 (AA'BB', 2H), 7.52 (dd, 1H, J = 8.6, 8.6 Hz), 7.18 (d, 1H, J = 8.6 Hz), 7.02–6.95 (AA'BB', 2H), 4.05–3.96 (m, 3H + 4H), 3.18–3.08 (br, 4H), 1.02 (s, 9H), 0.26 (s, 6H); $^{13}C\{^{1}H\}$ NMR ($CDCl_3$, 101 MHz): δ 168.3, 155.7, 149.9, 136.4, 133.3, 131.4, 129.8, 129.4, 128.3, 128.2, 127.3, 125.2, 121.4, 120.6, 115.7, 67.4, 53.7, 52.3, 25.7, 18.3, –4.4; IR (NaCl, cm^{-1}) 913, 1118, 1199, 1236, 1261, 1514, 1721, 2955; HRMS (ESI) *m/z*: $[M+H]^+$ Calcd for $C_{28}H_{36}NO_4Si^+$ 478.2408; Found 478.2405.

Methyl 3-(4-((benzylsulfonyl)oxy)phenyl)-5-morpholino-1-naphthoate (**15**)

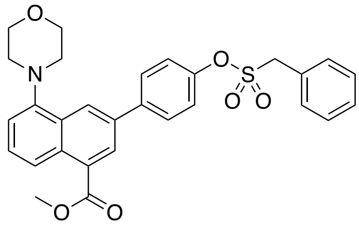

Yield: 75% (5.7 mg, 11 μmol); Yellow solid; Mp 96–98 °C; TLC R_f 0.27 (*n*-hexane/EtOAc = 3/1); ^{1}H NMR ($CDCl_3$, 400 MHz): δ 8.66 (d, 1H, J = 2.0 Hz), 8.58 (d, 1H, J = 8.7 Hz), 8.35 (d, 1H, J = 2.0 Hz), 7.73–7.68 (AA'BB', 2H), 7.59–7.43 (m, 6H), 7.29–7.24 (m, 2H), 7.22 (d, 1H, J = 8.7 Hz), 4.59 (s, 2H), 4.05–3.97 (m, 3H + 4H), 3.15–3.08 (br, 4H); $^{13}C\{^{1}H\}$ NMR ($CDCl_3$, 101 MHz): δ 168.0, 150.1, 148.7, 139.6, 135.3, 131.8, 130.9, 129.8, 129.4, 129.3, 129.1, 128.7, 128.6, 128.0, 127.1, 126.2, 122.6, 121.4, 116.0, 67.4, 56.9, 53.7, 52.3; IR (NaCl,

cm^{-1}) 867, 1115, 1150, 1203, 1236, 1296, 1372, 1716; HRMS (ESI) *m/z*: $[M+H]^+$ Calcd for $C_{29}H_{28}NO_6S^+$ 518.1632; Found 518.1632.

References for the Supporting Information

S1 S. Yoshida, Y. Nakamura, K. Uchida, Y. Hazama, T. Hosoya, *Org. Lett.* **2016**, *18*, 6212.

S2 X. Li, Y. Sun, X. Huang, L. Zhang, L. Kong, B. Peng, *Org. Lett*. **2017**, *19*, 838.

S3 H. Lackmann, J. Engelking, H. Menzel, *Materials Science and Engineering: C* **1999**, *8–9*, 127.

S4 C. Desmarets, B. Champagne, A. Walcarius, C. Bellouard, R. Omar-Amrani, A. Ahajji, Y. Fort, R. Schneider, *J. Org. Chem.* **2006**, *71*, 1351.

S5 L. Hie, N. F. F. Nathel, X. Hong, Y.-F. Yang, K. N. Houk, N. K. Garg, *Angew. Chem., Int. Ed.* **2016**, *55*, 2810.

S6 D. Lücke, A. S. Campbell, M. Petzold, R. Sarpong, *Org. Lett.* **2023**, *25*, 7349.

^{1}H and ^{13}C NMR Spectra of New Compounds

^{1}H NMR (400 MHz) and ^{13}C NMR (101 MHz) spectra of 3-(octahydroisoquinolin-2(1*H*)-yl)-2-(trimethylsilyl)phenyl trifluoromethanesulfonate (**2c**) ($CDCl_3$)

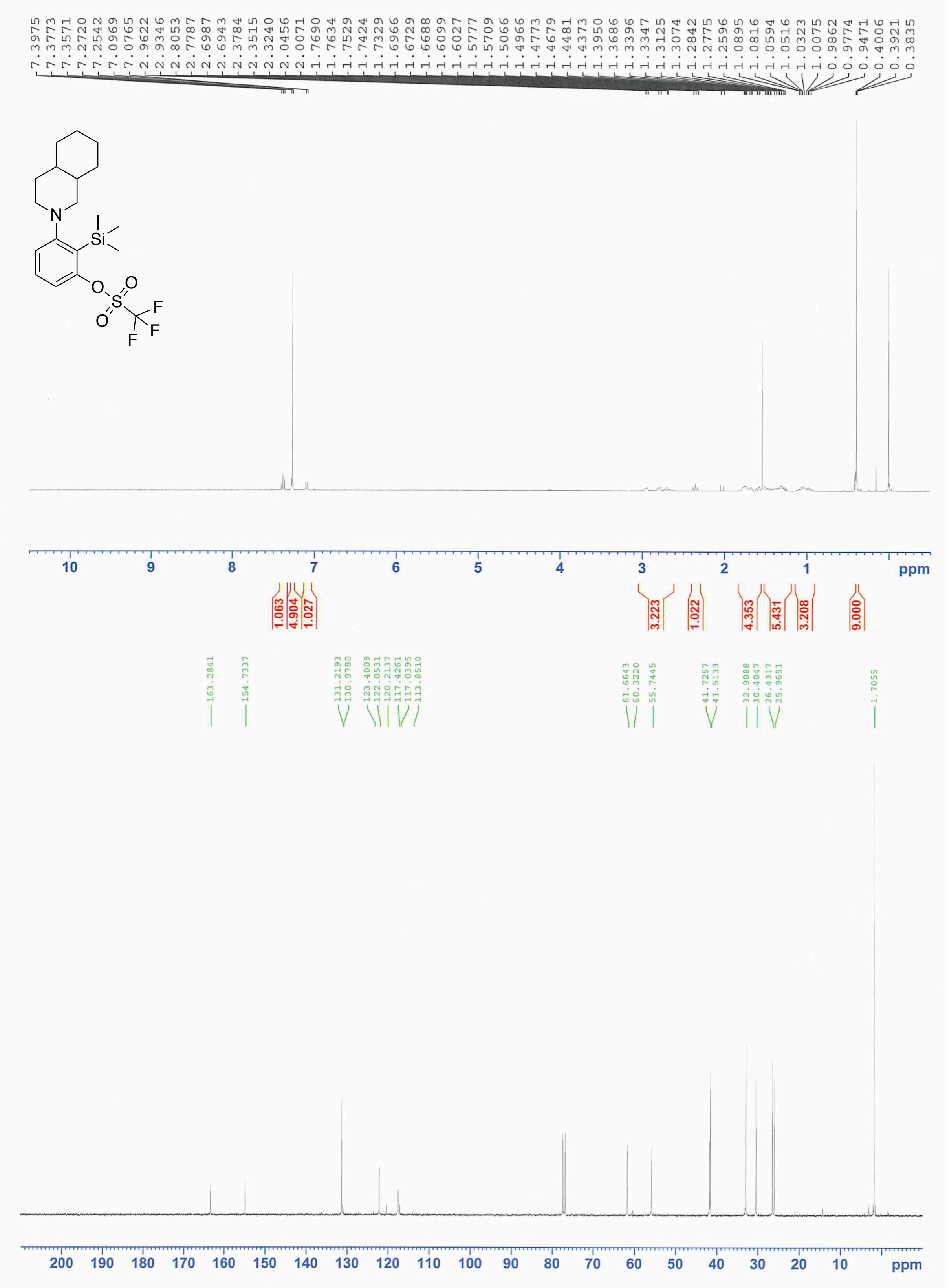

^{1}H NMR (400 MHz) and ^{13}C NMR (101 MHz) spectra of 4-(1,4-diphenyl-1,4-dihydro-1,4-epoxynaphthalen-5-yl)morpholine (**4a**) ($CDCl_3$)

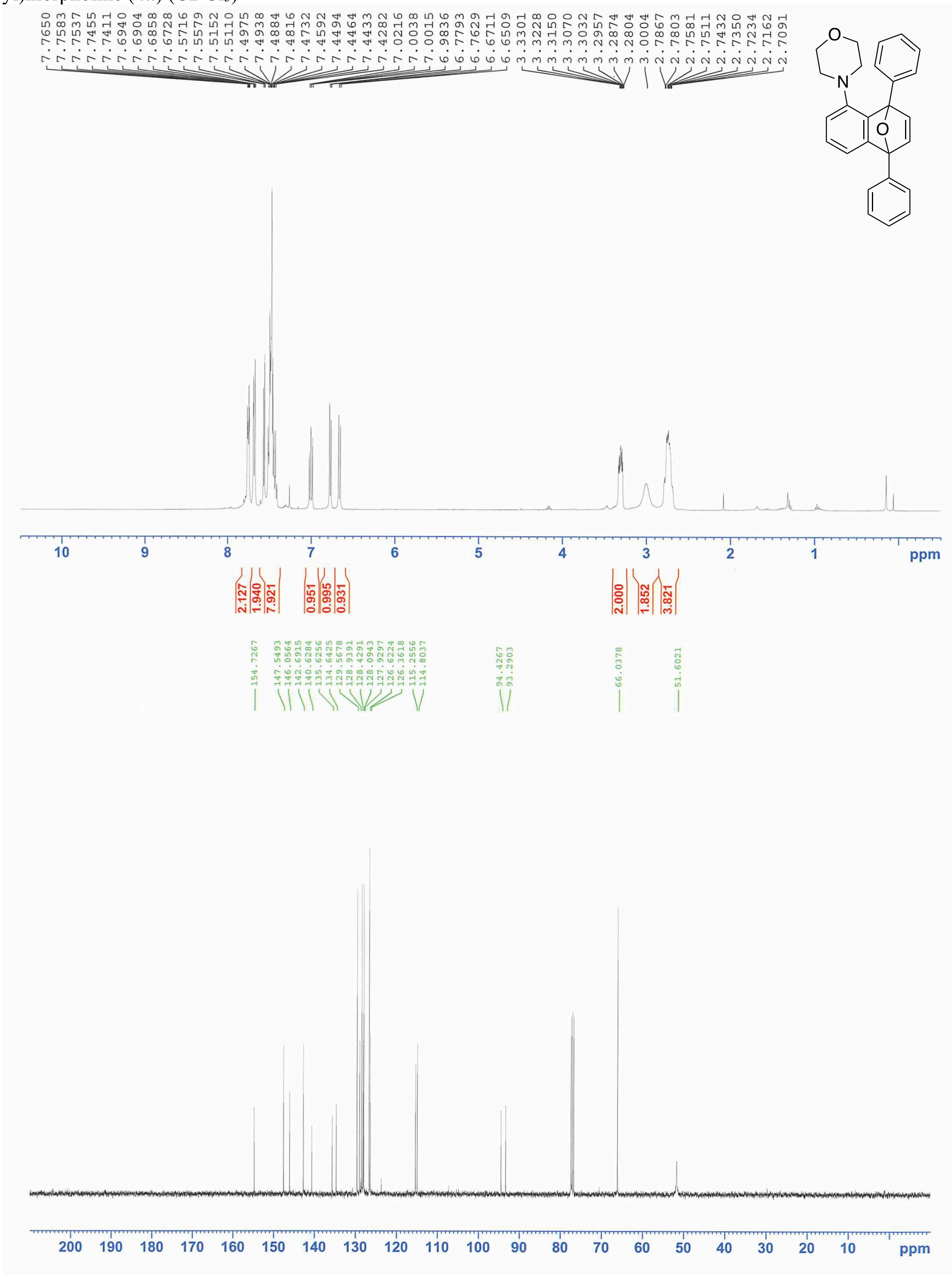

^{1}H NMR (400 MHz) and ^{13}C NMR (101 MHz) spectra of 4-(1,4-dihydro-1,4-epoxynaphthalen-5-yl)morpholine (**4b**) ($CDCl_3$)

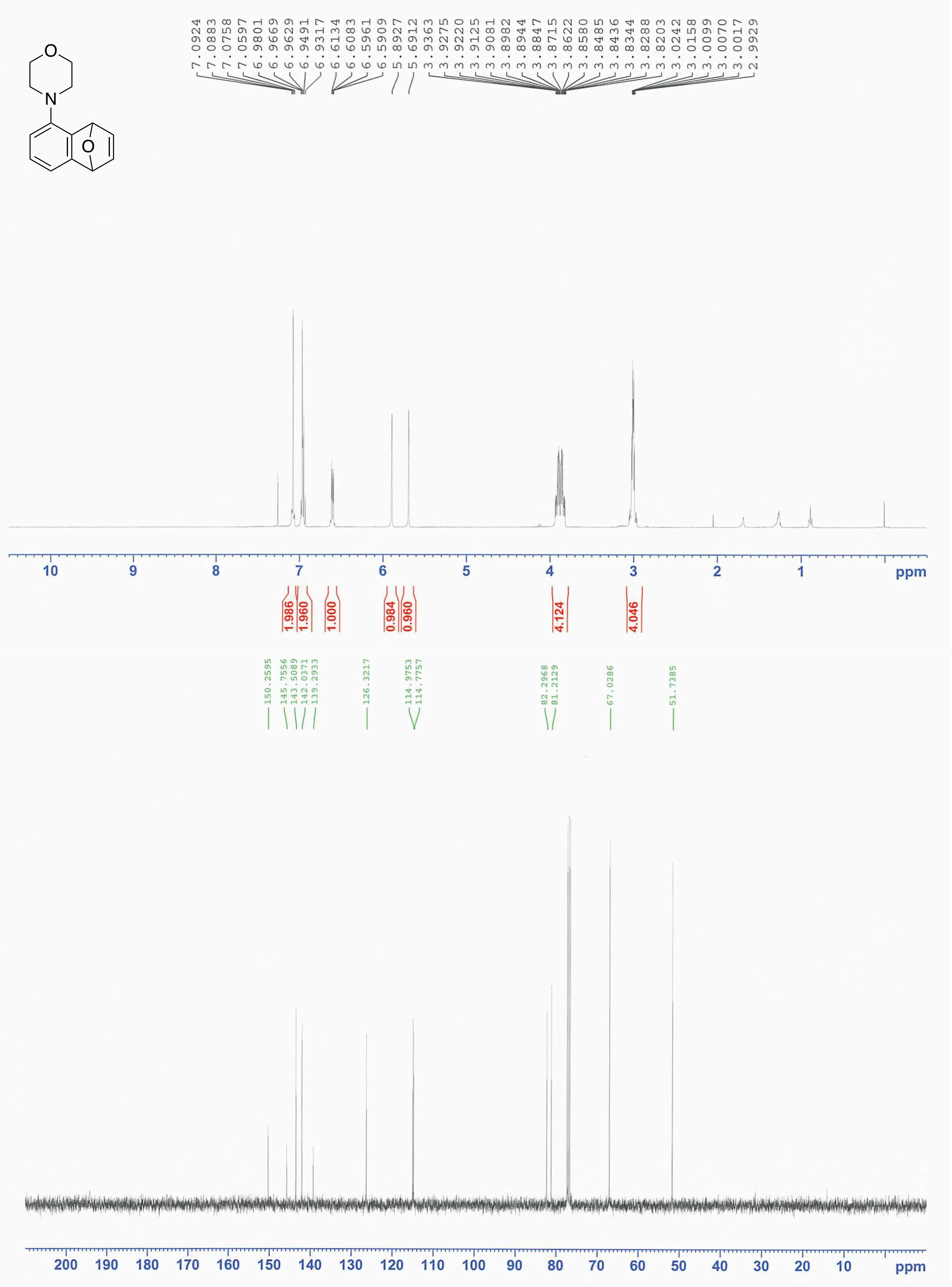

^{1}H NMR (400 MHz) and ^{13}C NMR (101 MHz) spectra of 4-(9,10-diphenyl-9,10-dihydro-9,10-epoxyanthracen-1-yl)morpholine (**4c**) ($CDCl_3$)

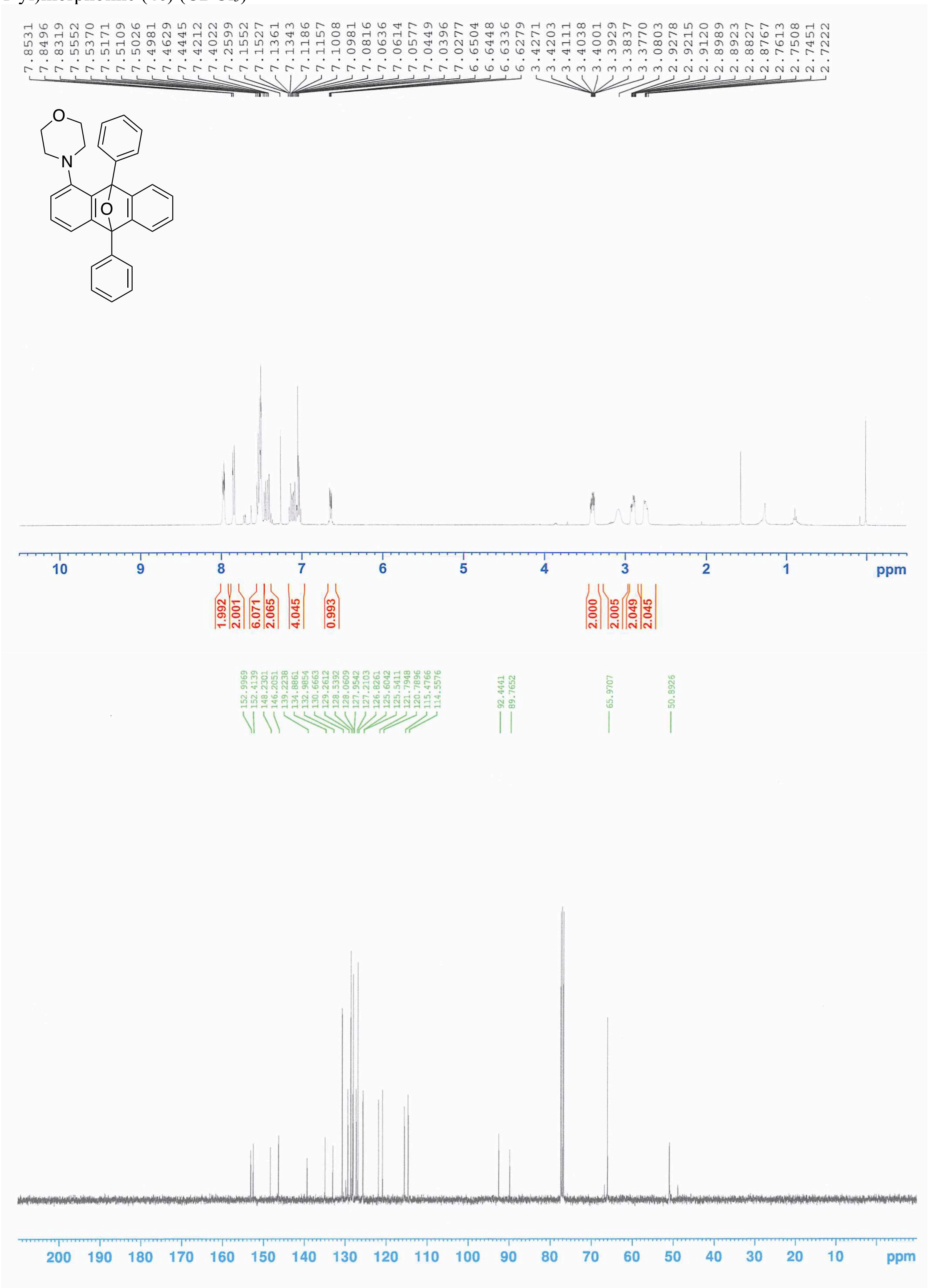

^1H NMR (400 MHz) and ^{13}C NMR (101 MHz) spectra of methyl 8-morpholino-1,4-epoxynaphthalene-1(4*H*)-carboxylate (**4d**) ($CDCl_3$)

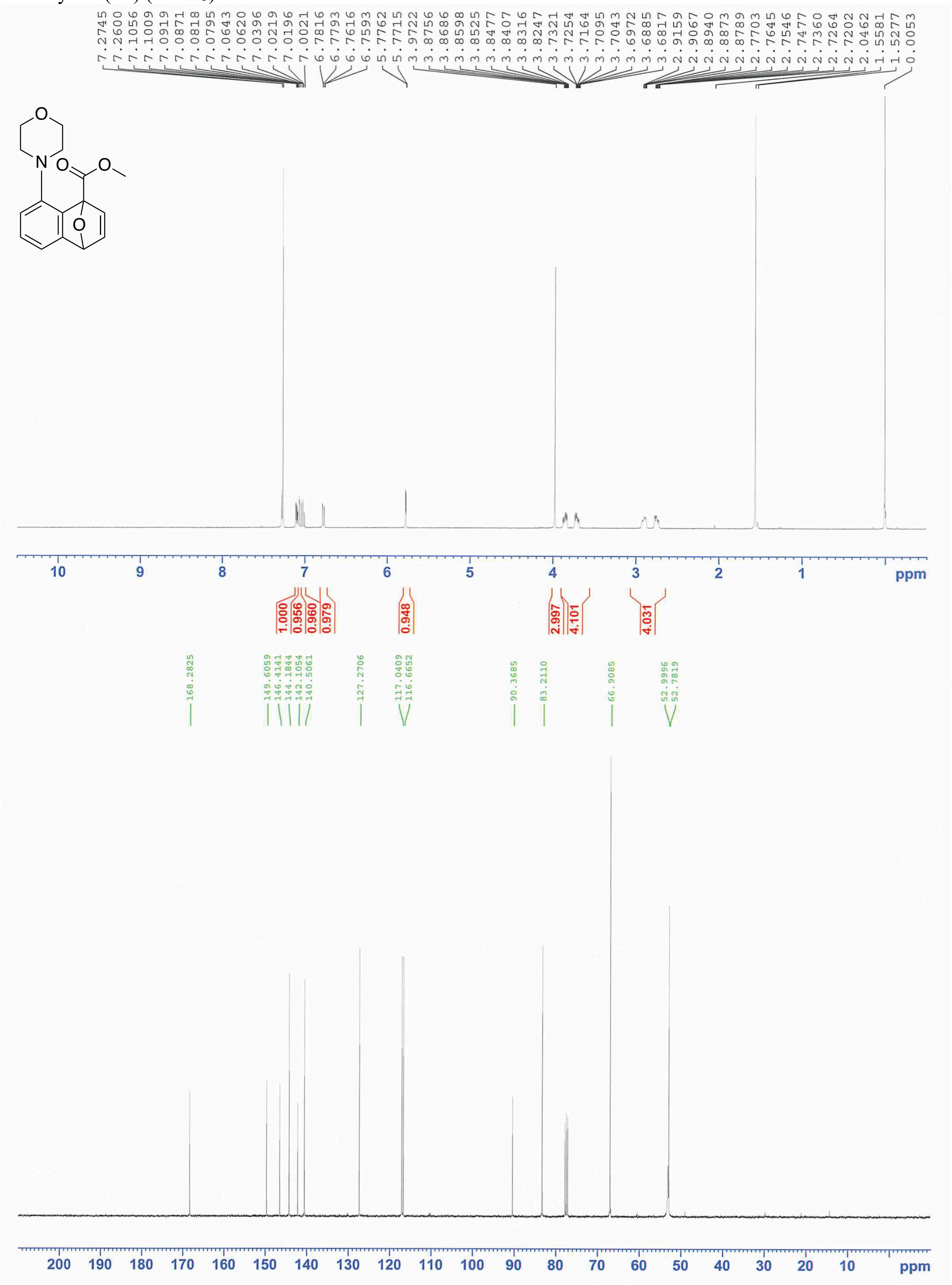

^{1}H NMR (400 MHz) and ^{13}C NMR (101 MHz) spectra of methyl 5-morpholino-1,4-epoxynaphthalene-1(4*H*)-carboxylate (**5d**) ($CDCl_3$)

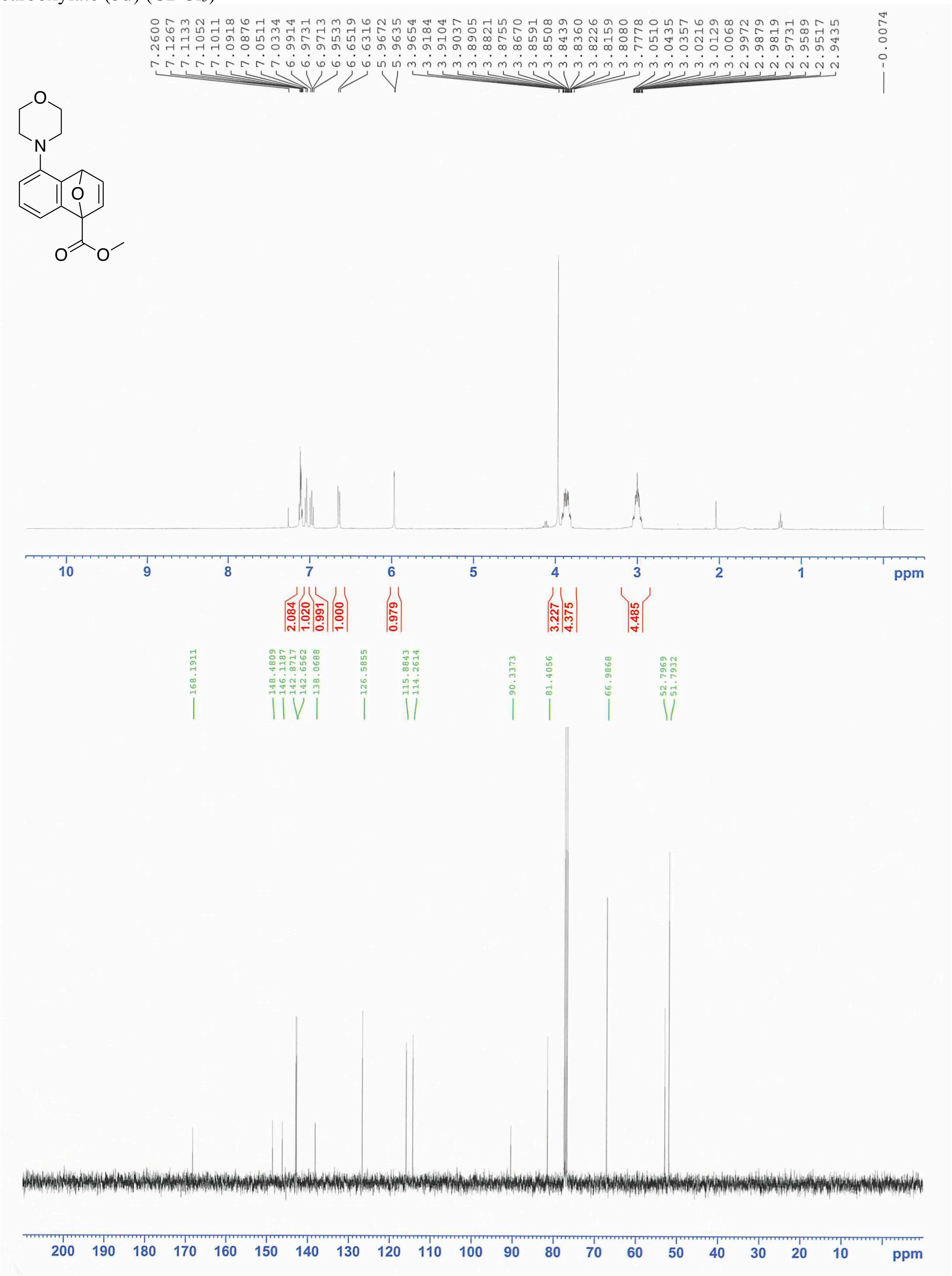

^{1}H NMR (400 MHz) and ^{13}C NMR (101 MHz) spectra of 4-(4-butyl-1,4-dihydro-1,4-epoxynaphthalen-5-yl)morpholine (**4e**) ($CDCl_3$)

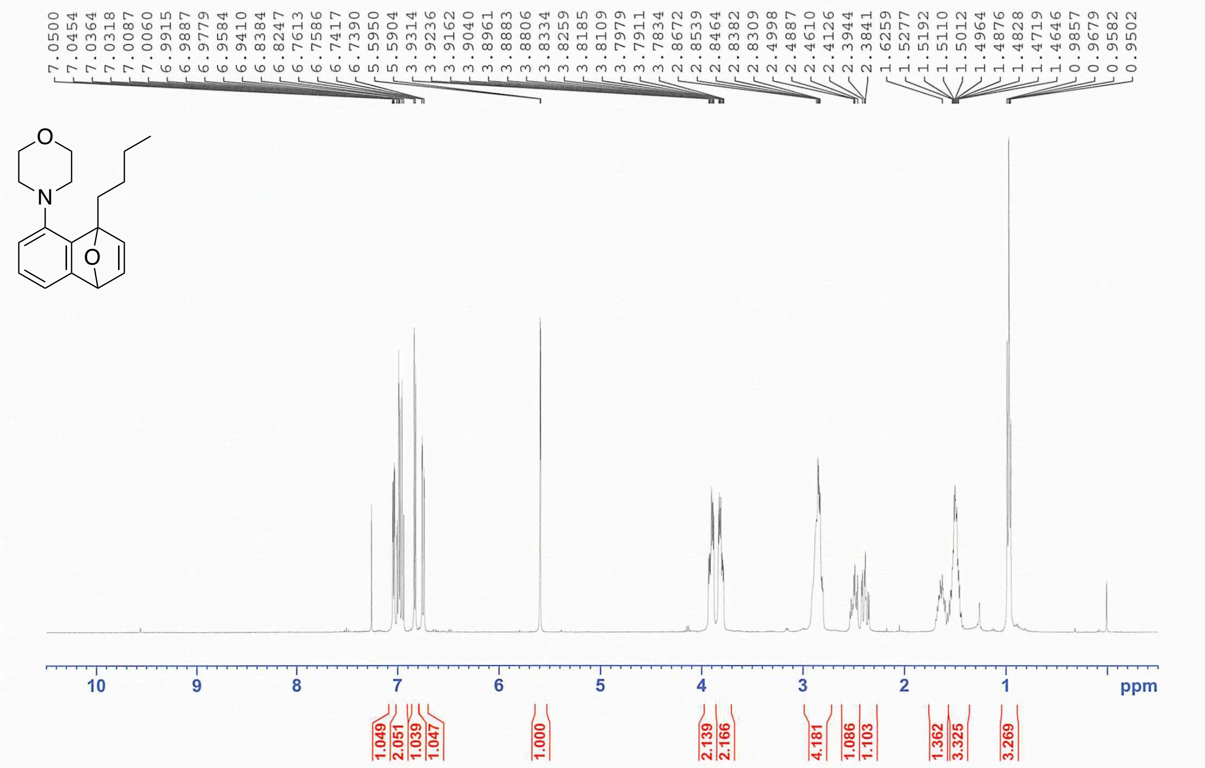

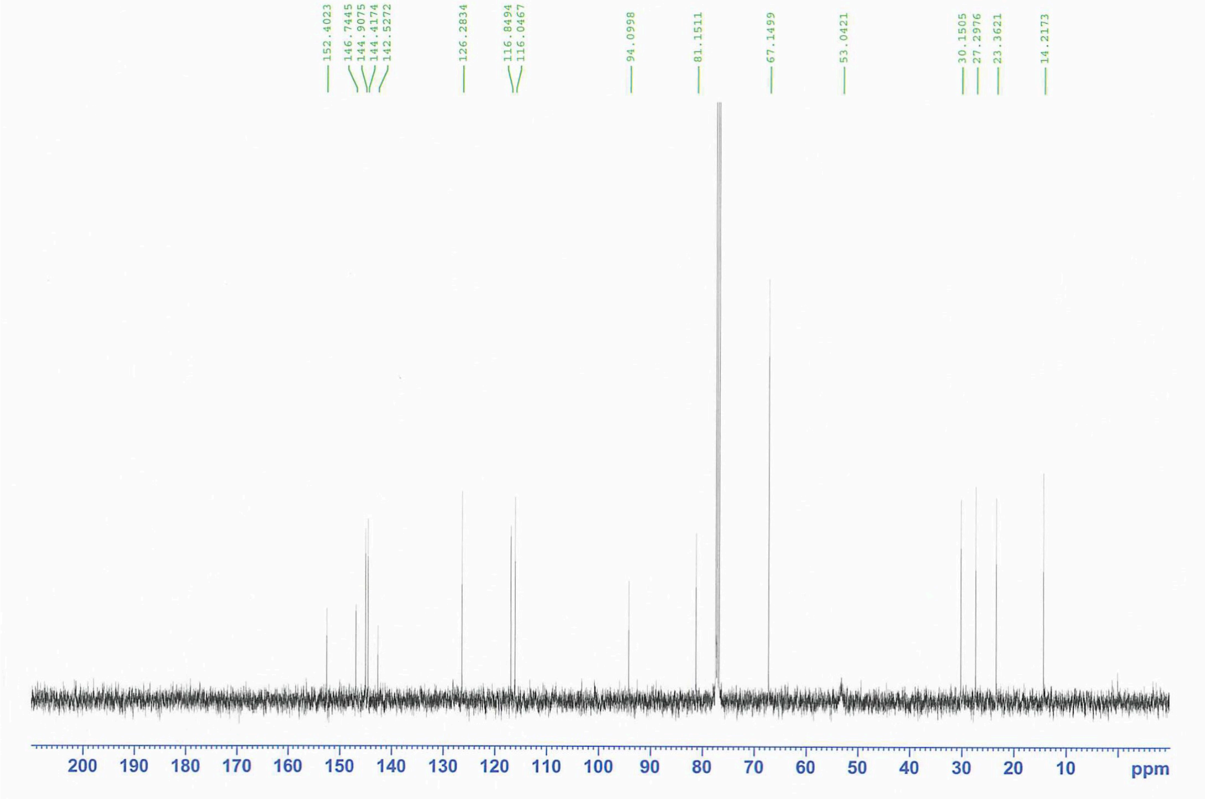

^{1}H NMR (400 MHz) and ^{13}C NMR (101 MHz) spectra of 4-(1-butyl-1,4-dihydro-1,4-epoxynaphthalen-5-yl)morpholine (**5e**) ($CDCl_3$)

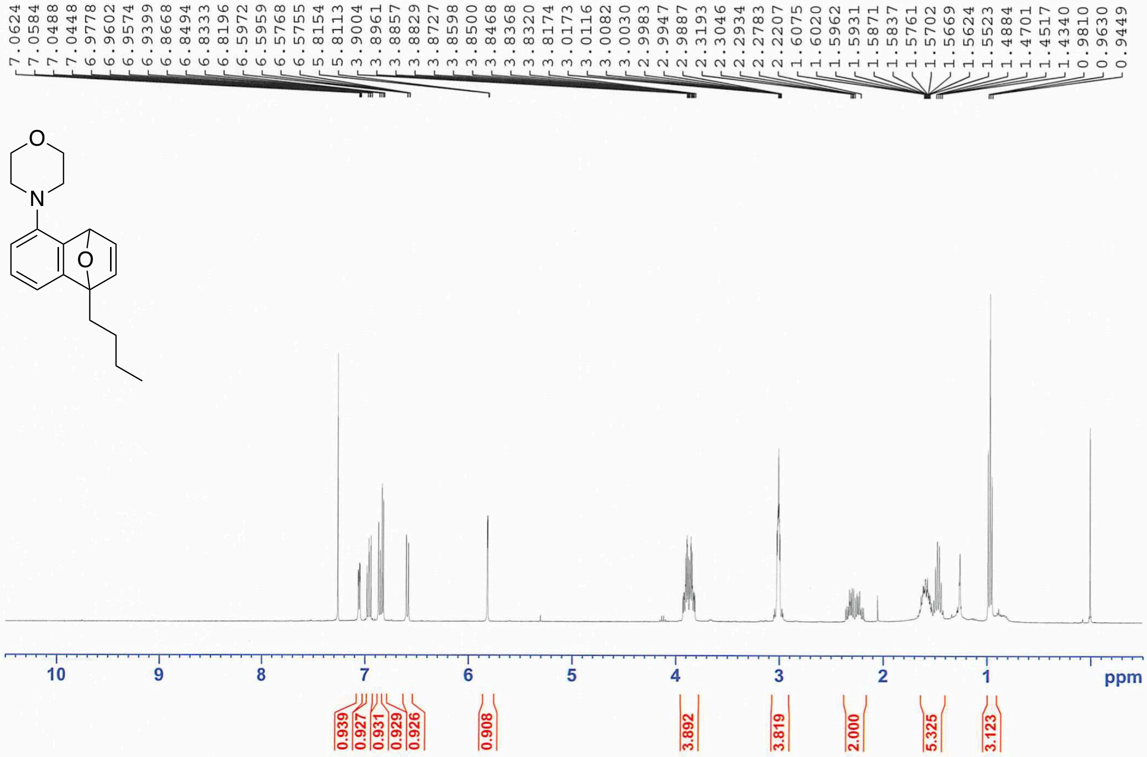

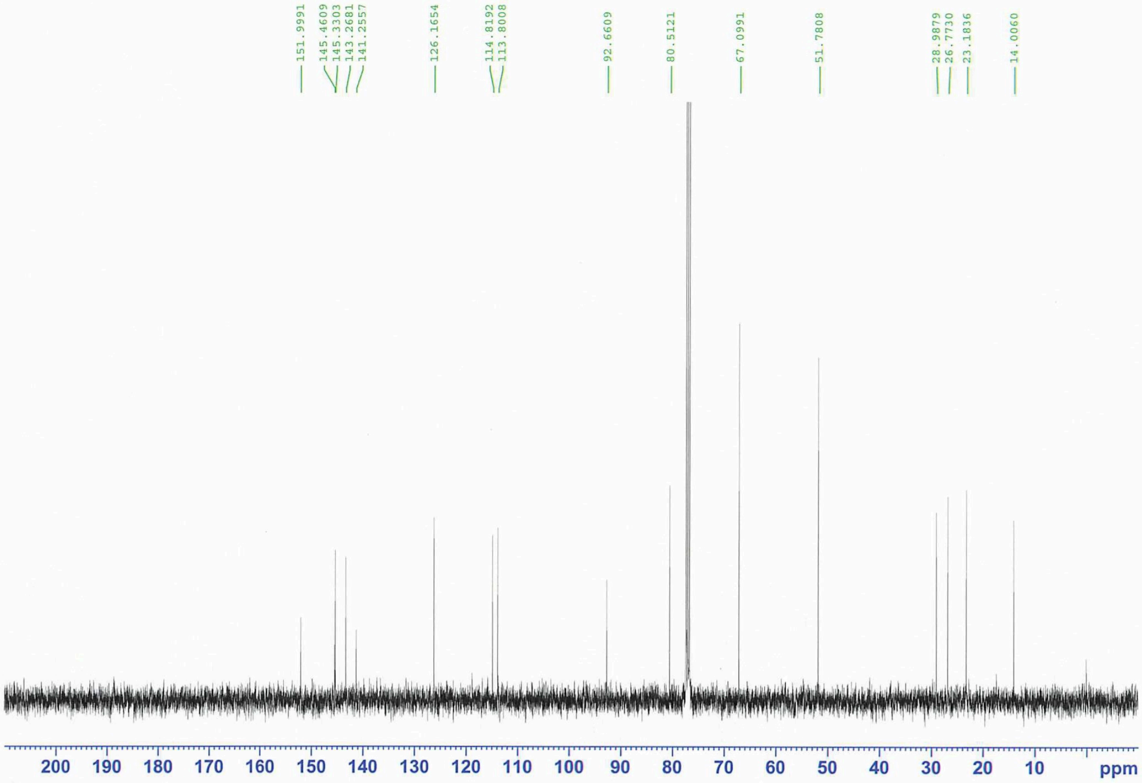

^{1}H NMR (400 MHz) and ^{13}C NMR (101 MHz) spectra of 8-morpholino-1,4-epoxynaphthalene-1(4*H*)-carbonitrile (**4f**) ($CDCl_3$)

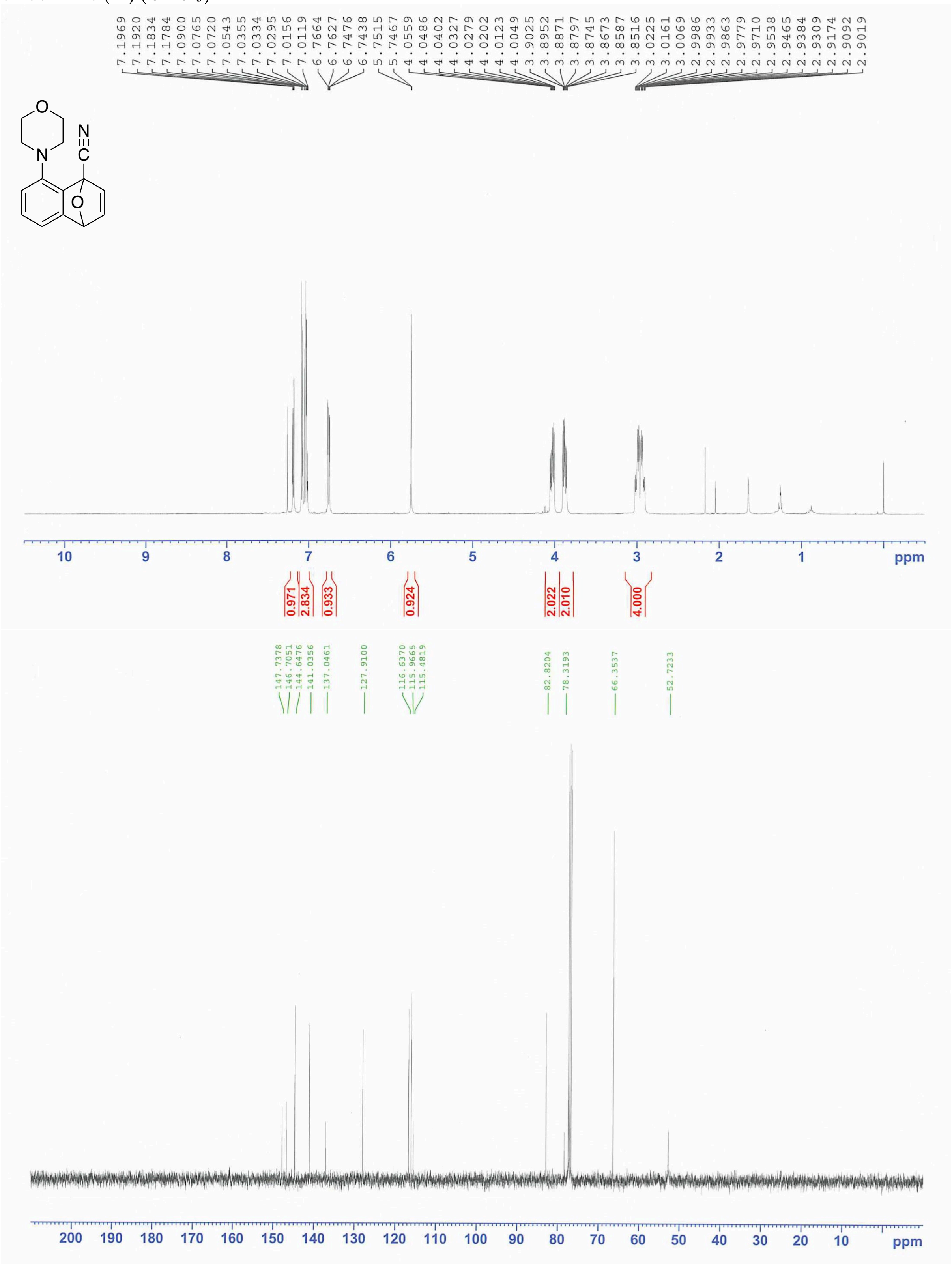

^{1}H NMR (400 MHz) and ^{13}C NMR (101 MHz) spectra of 5-morpholino-1,4-epoxynaphthalene-1(4*H*)-carbonitrile (**5f**) ($CDCl_3$)

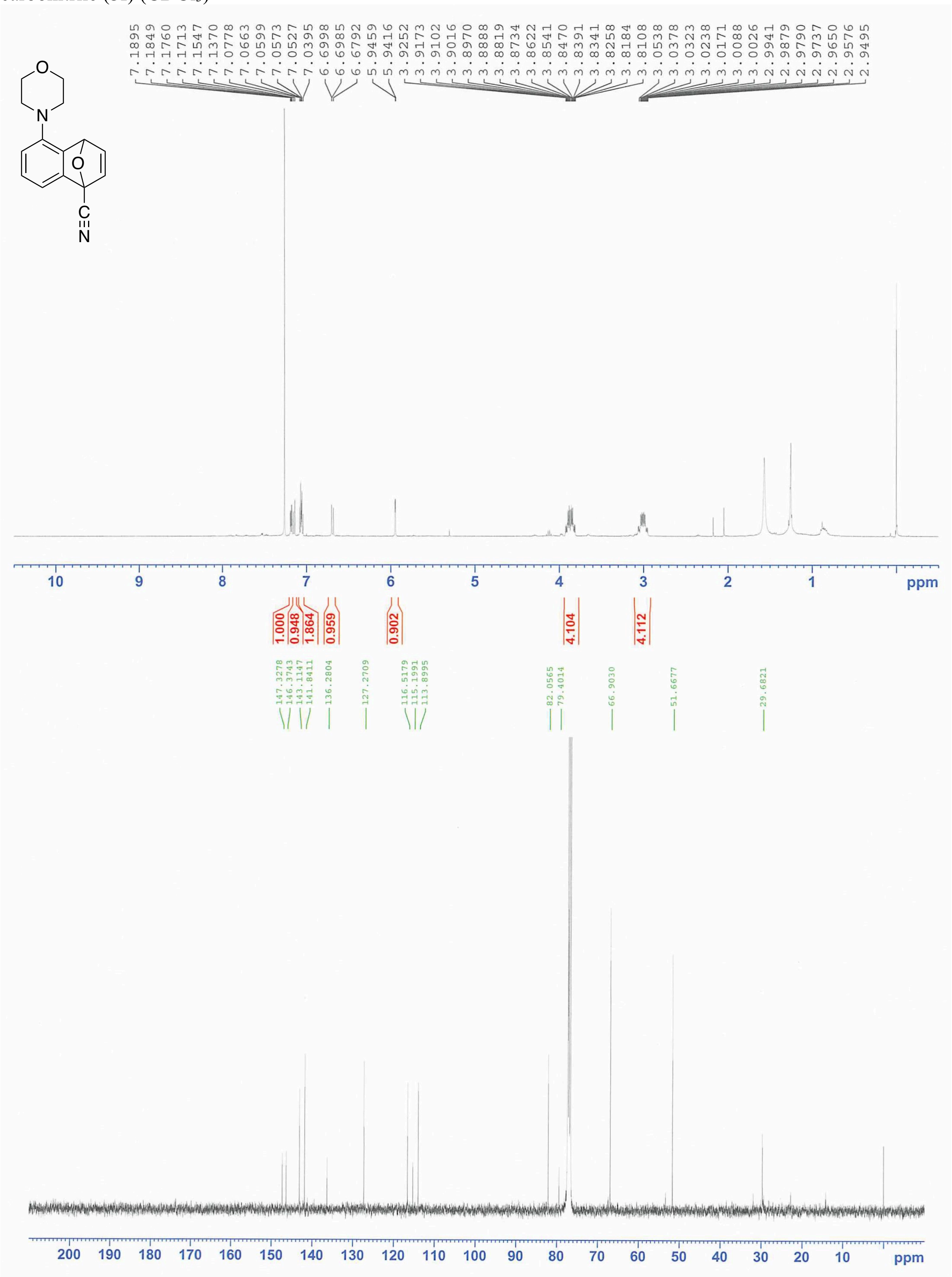

^{1}H NMR (400 MHz) and ^{13}C NMR (101 MHz) spectra of 4-(3,4-dimethyl-1,4-dihydro-1,4-epoxynaphthalen-5-yl)morpholine (**4h**) ($CDCl_3$)

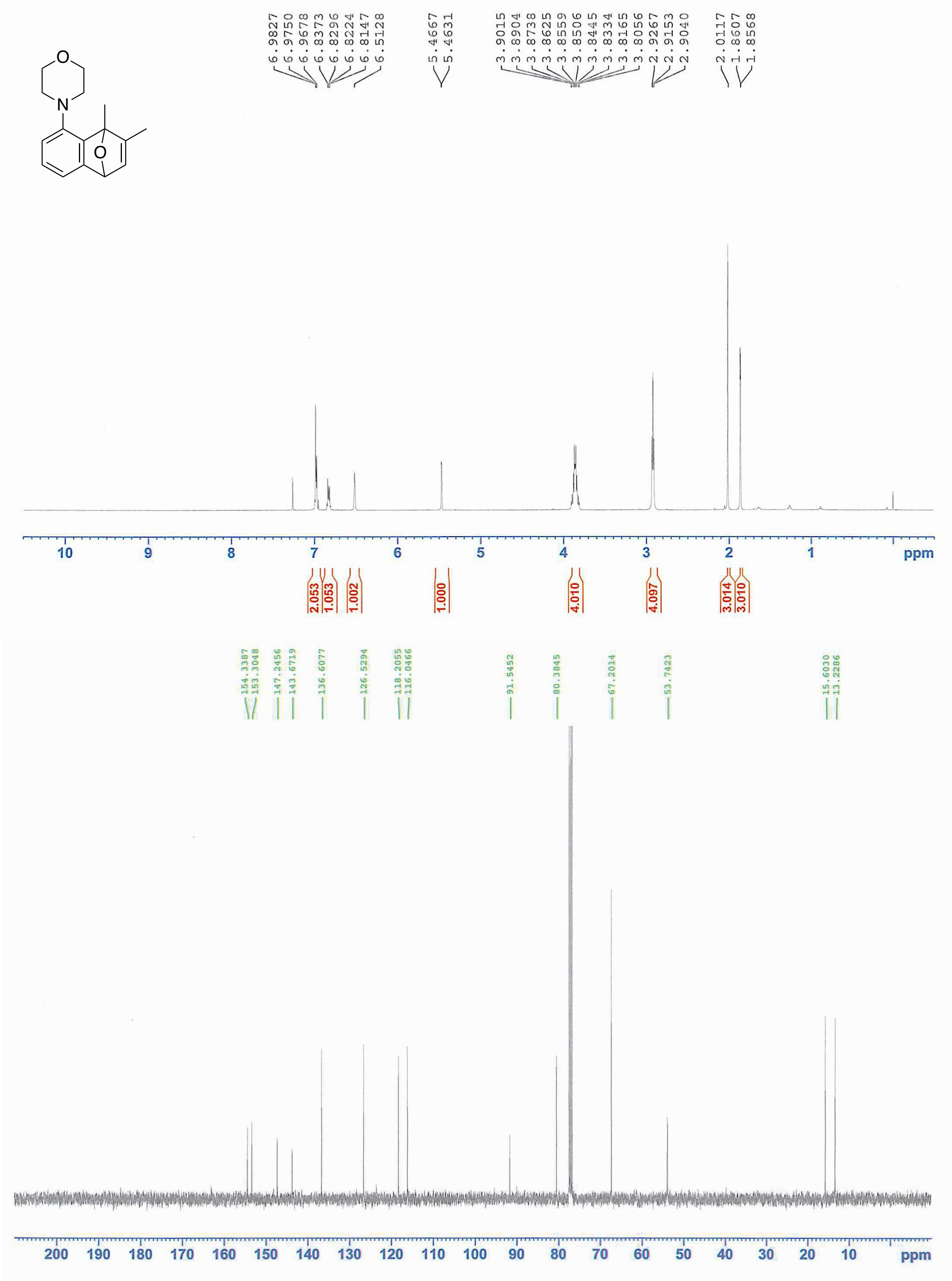

^{1}H NMR (400 MHz) and ^{13}C NMR (101 MHz) spectra of 4-(1,2-dimethyl-1,4-dihydro-1,4-epoxynaphthalen-5-yl)morpholine (**5h**) ($CDCl_3$)

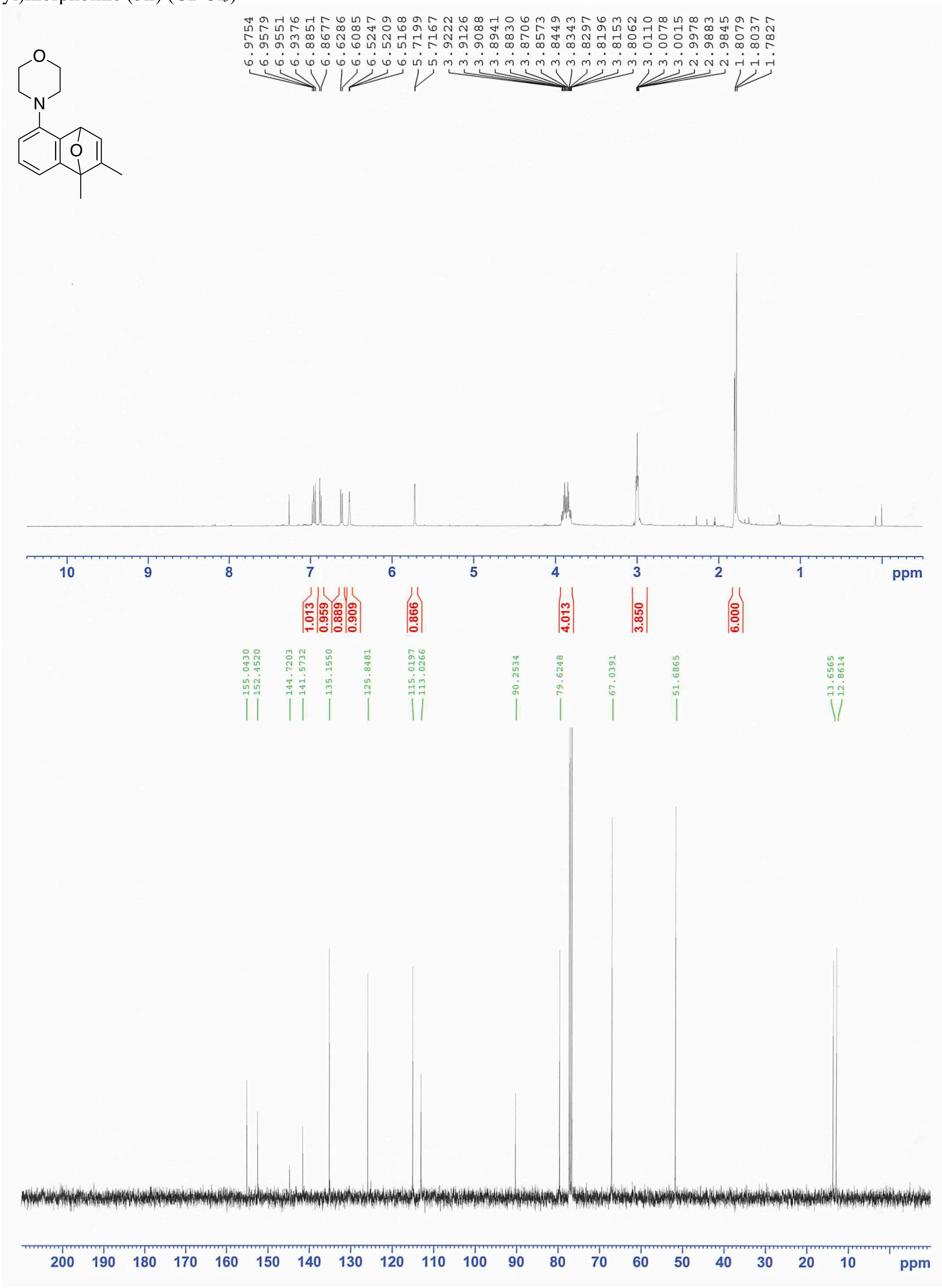

^{1}H NMR (400 MHz) and ^{13}C NMR (101 MHz) spectra of methyl 4-bromo-8-morpholino-1,4-epoxynaphthalene-1(4*H*)-carboxylate (**4i**) ($CDCl_3$)

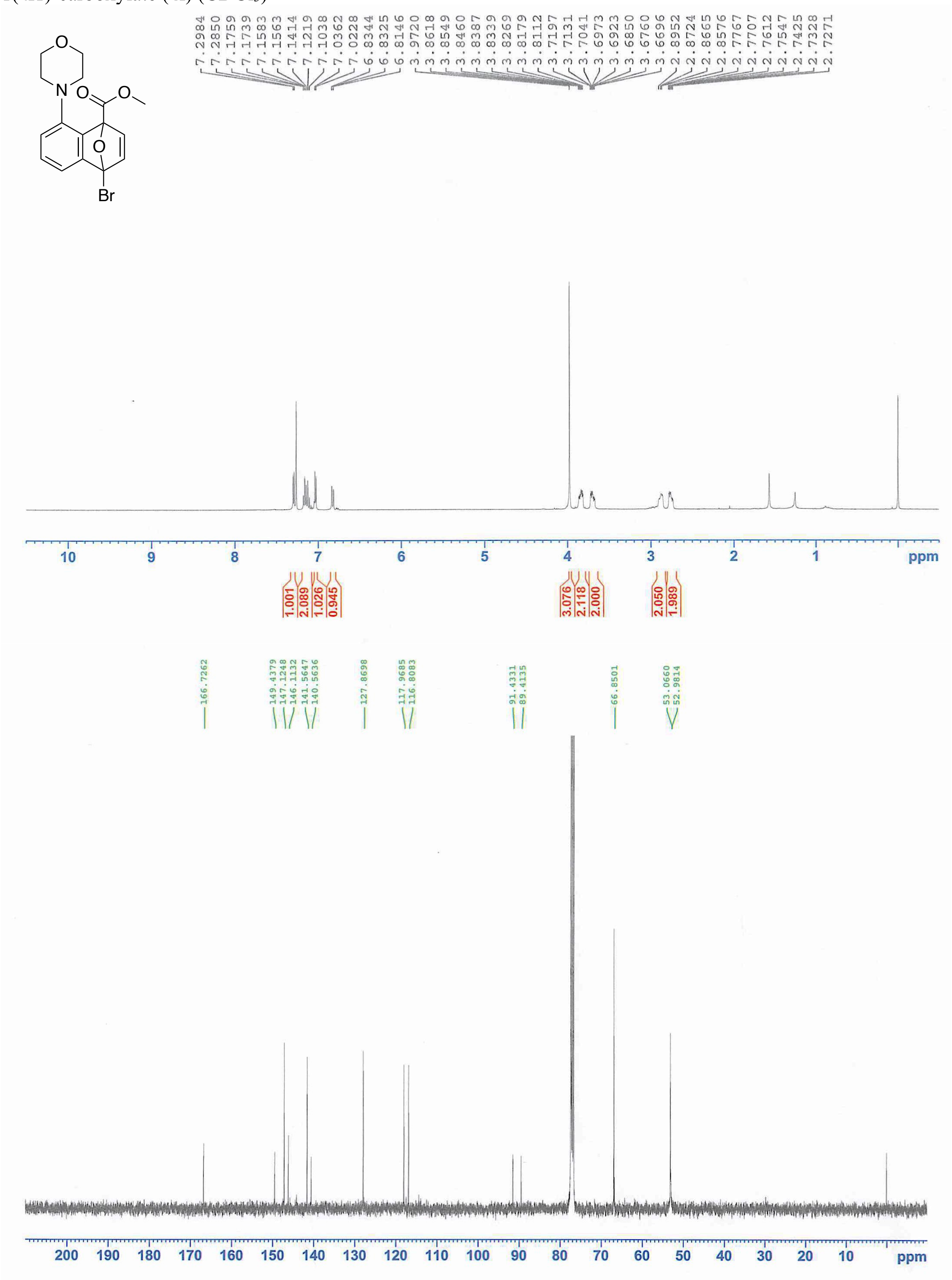

^{1}H NMR (400 MHz) and ^{13}C NMR (101 MHz) spectra of methyl 4-bromo-5-morpholino-1,4-epoxynaphthalene-1(4*H*)-carboxylate (**5i**) ($CDCl_3$)

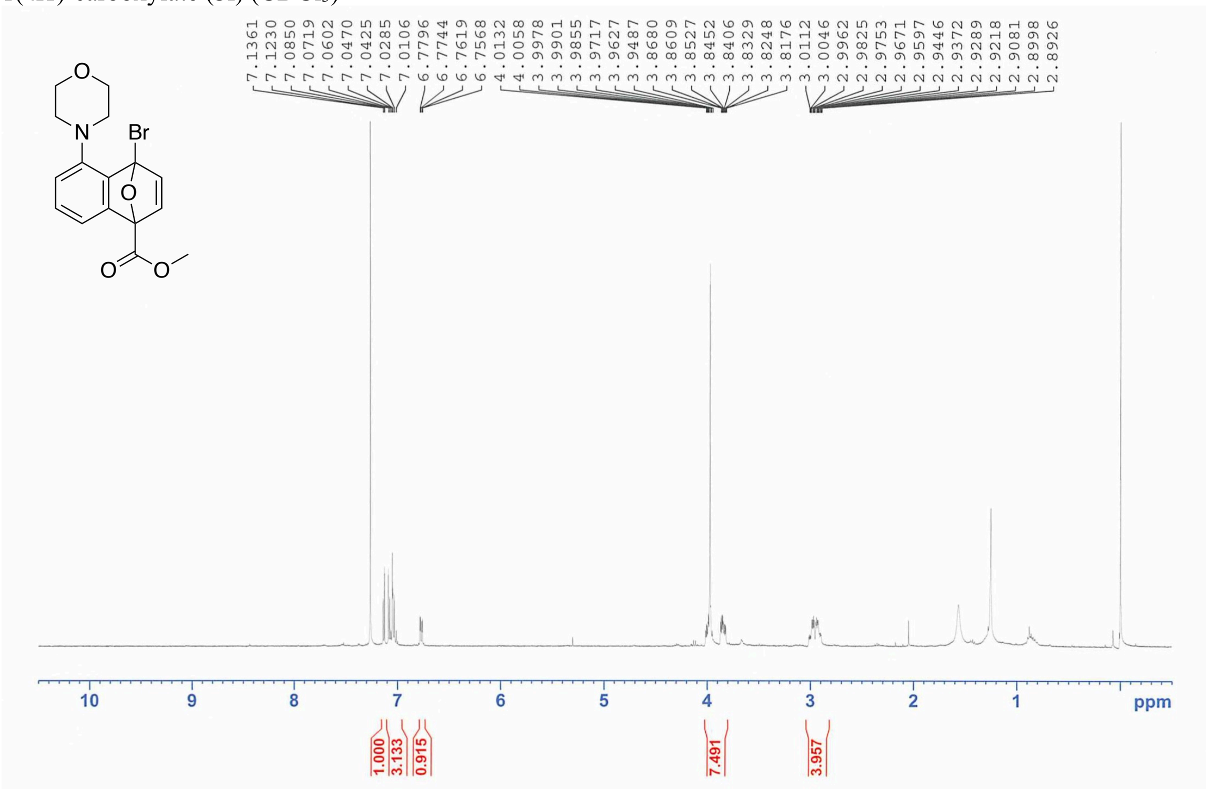

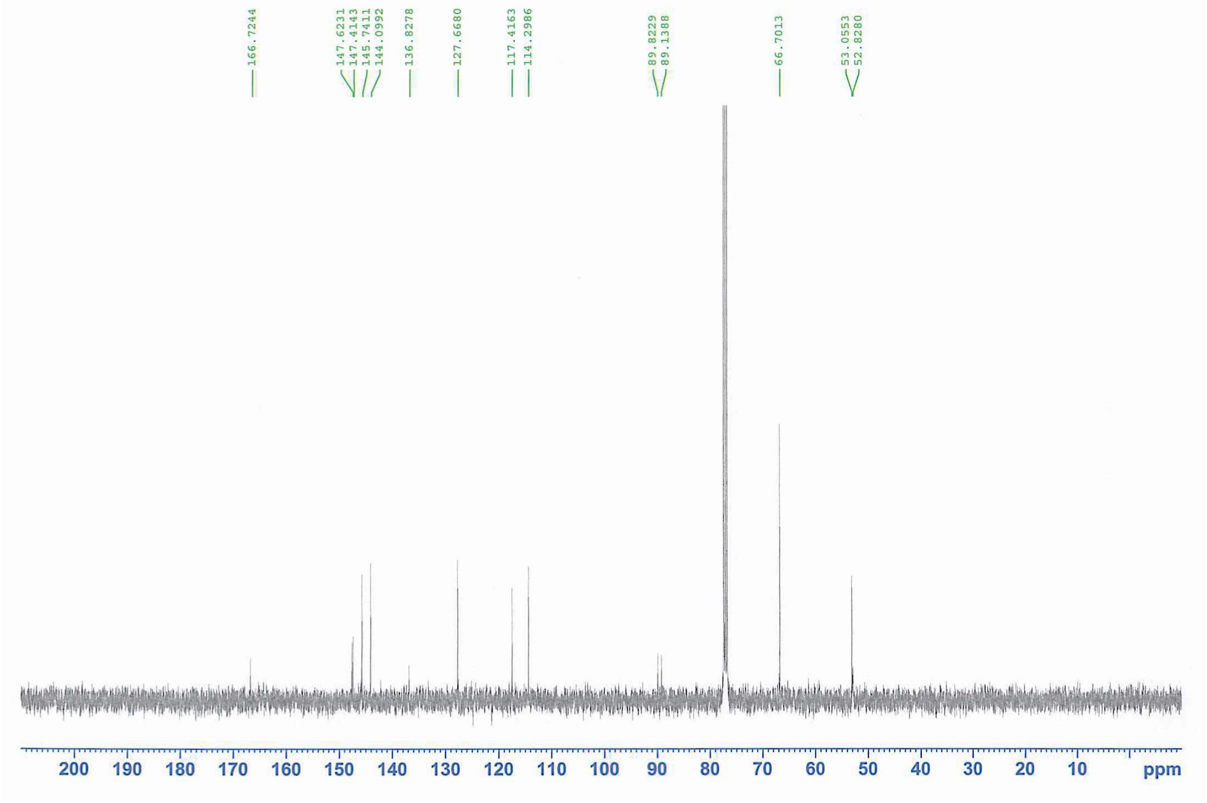

^{1}H NMR (400 MHz) and ^{13}C NMR (101 MHz) spectra of methyl 8-(dimethylamino)-1,4-epoxynaphthalene-1(4*H*)-carboxylate (**4j**) ($CDCl_3$)

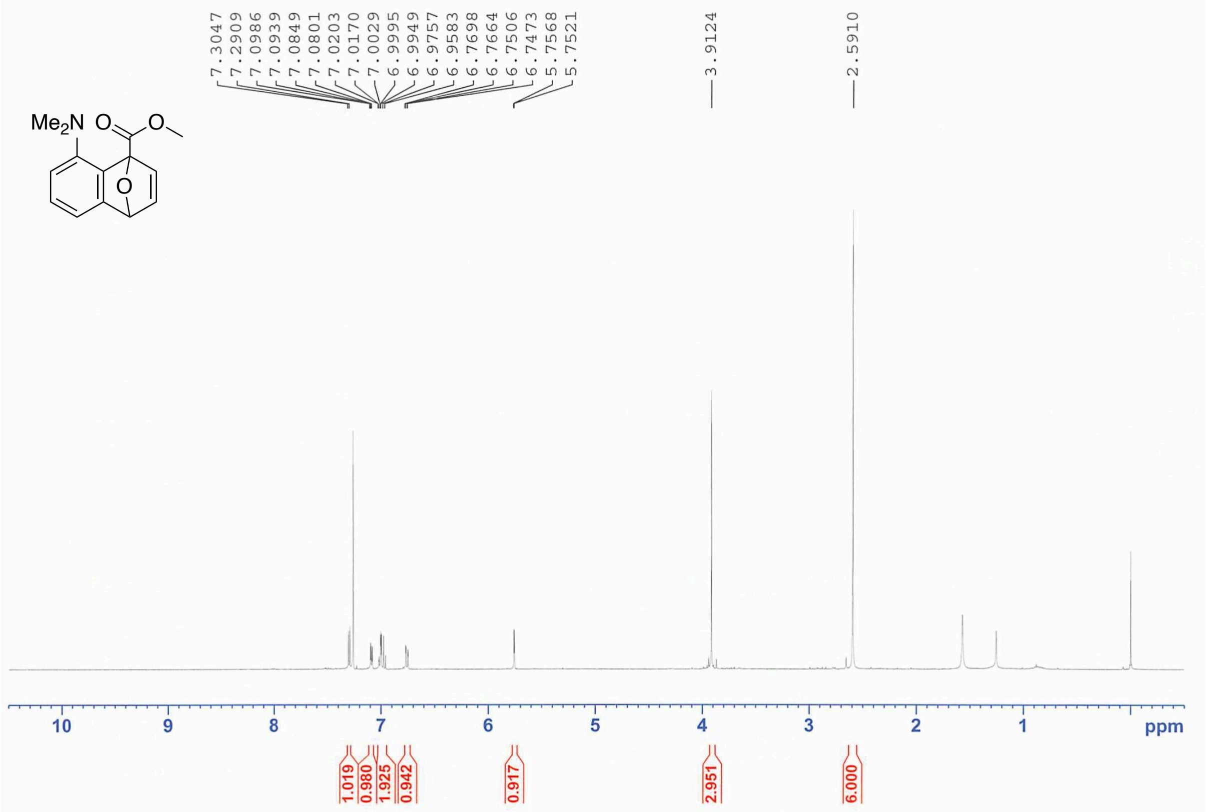

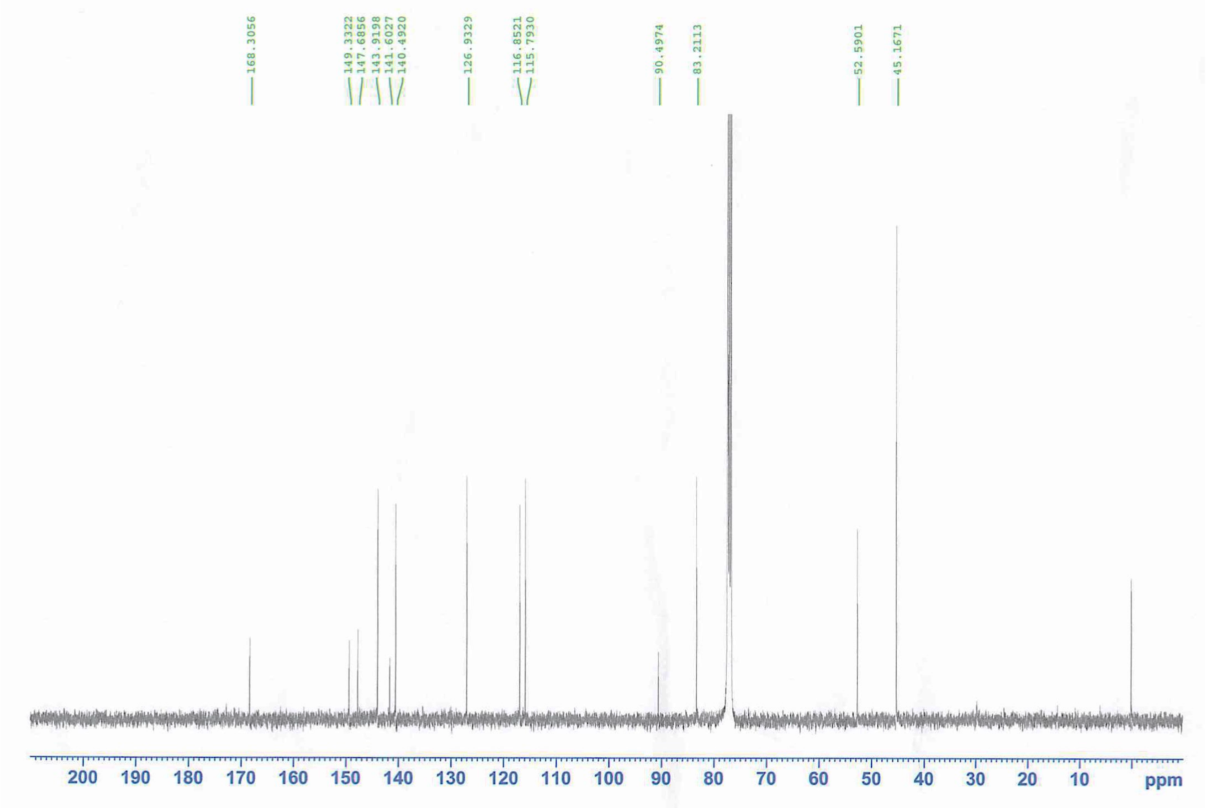

^{1}H NMR (400 MHz) and ^{13}C NMR (101 MHz) spectra of 4-butyl-*N*,*N*-dimethyl-1,4-dihydro-1,4-epoxynaphthalen-5-amine (**4k**) ($CDCl_3$)

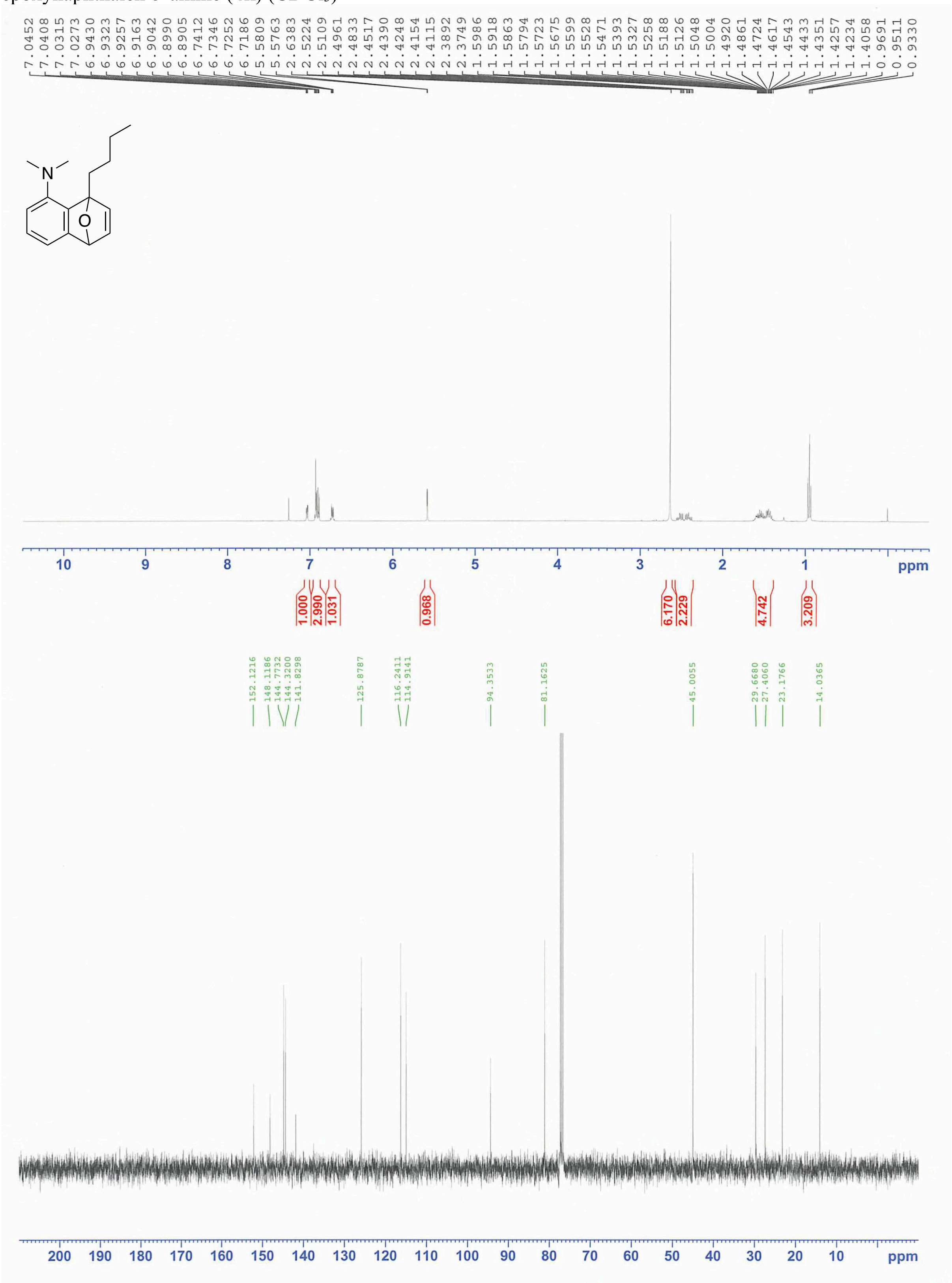

^{1}H NMR (400 MHz) and ^{13}C NMR (101 MHz) spectra of methyl 8-(octahydroisoquinolin-2(1*H*)-yl)-1,4-epoxynaphthalene-1(4*H*)-carboxylate (**4l**) ($CDCl_3$)

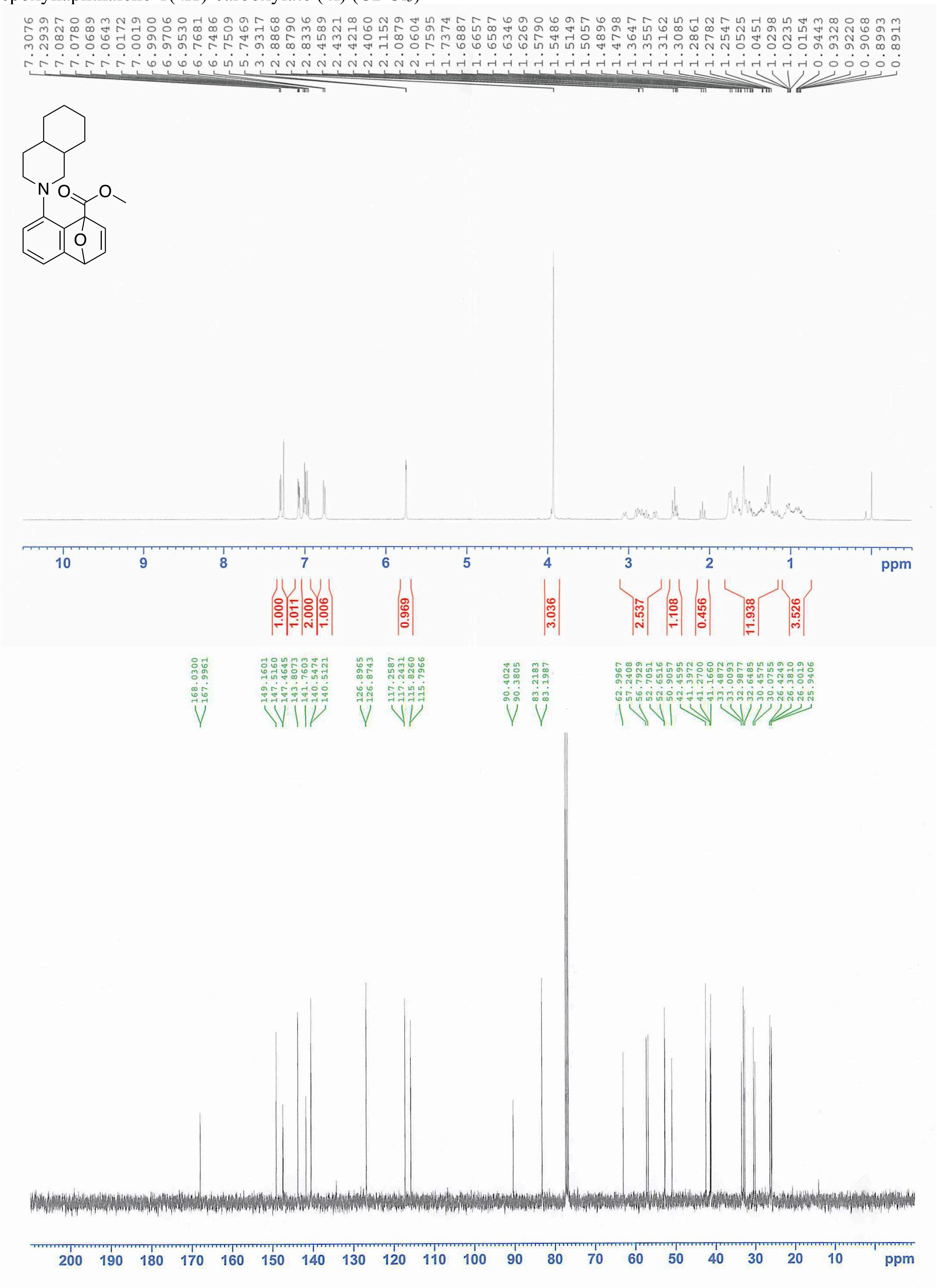

^{1}H NMR (400 MHz) and ^{13}C NMR (101 MHz) spectra of methyl 5-(octahydroisoquinolin-2(1*H*)-yl)-1,4-epoxynaphthalene-1(4*H*)-carboxylate (**5l**) ($CDCl_3$)

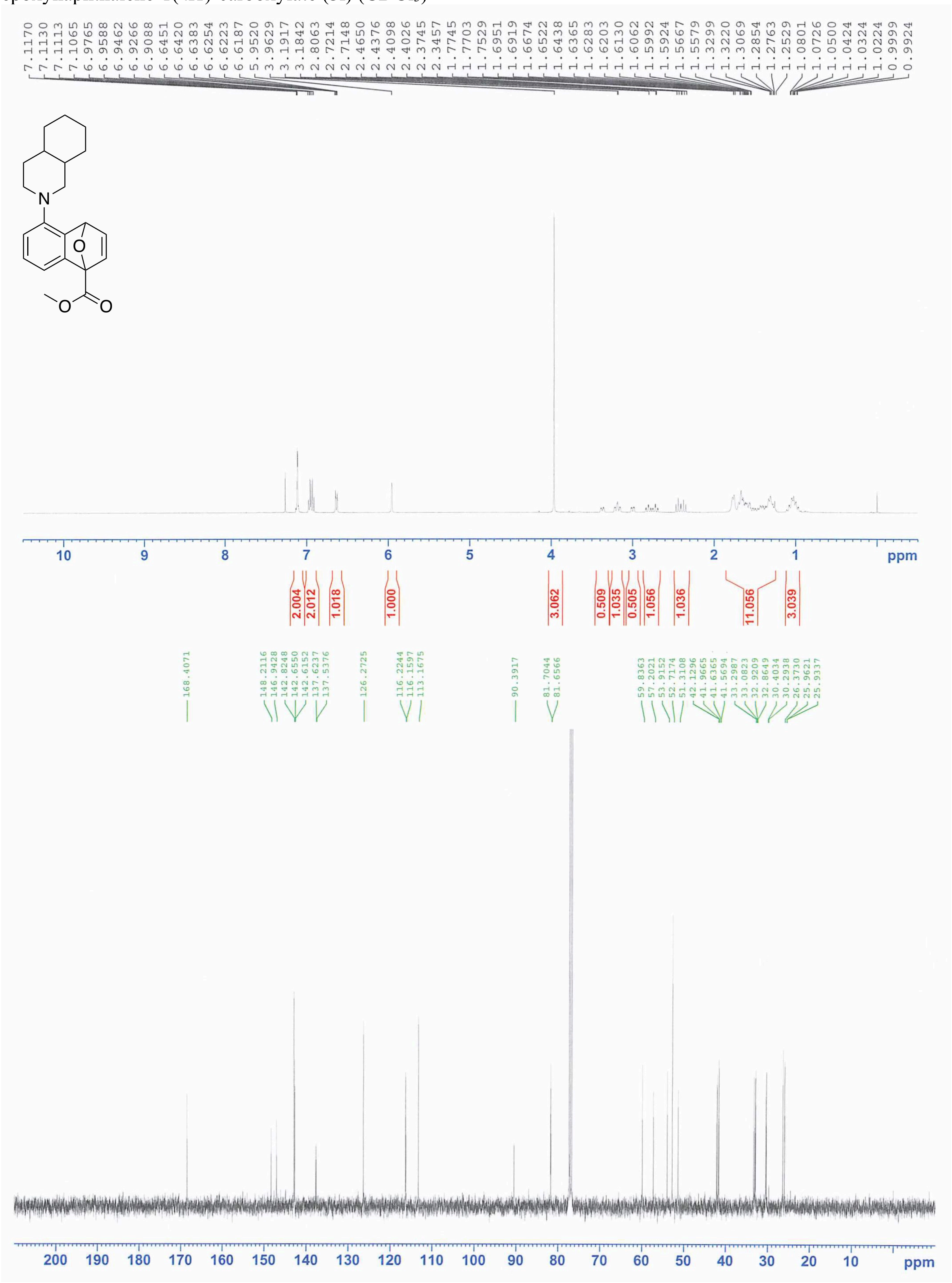

^{1}H NMR (400 MHz) and ^{13}C NMR (101 MHz) spectra of 2-(4-butyl-1,4-dihydro-1,4-epoxynaphthalen-5-yl)decahydroisoquinoline (**4m**) ($CDCl_3$)

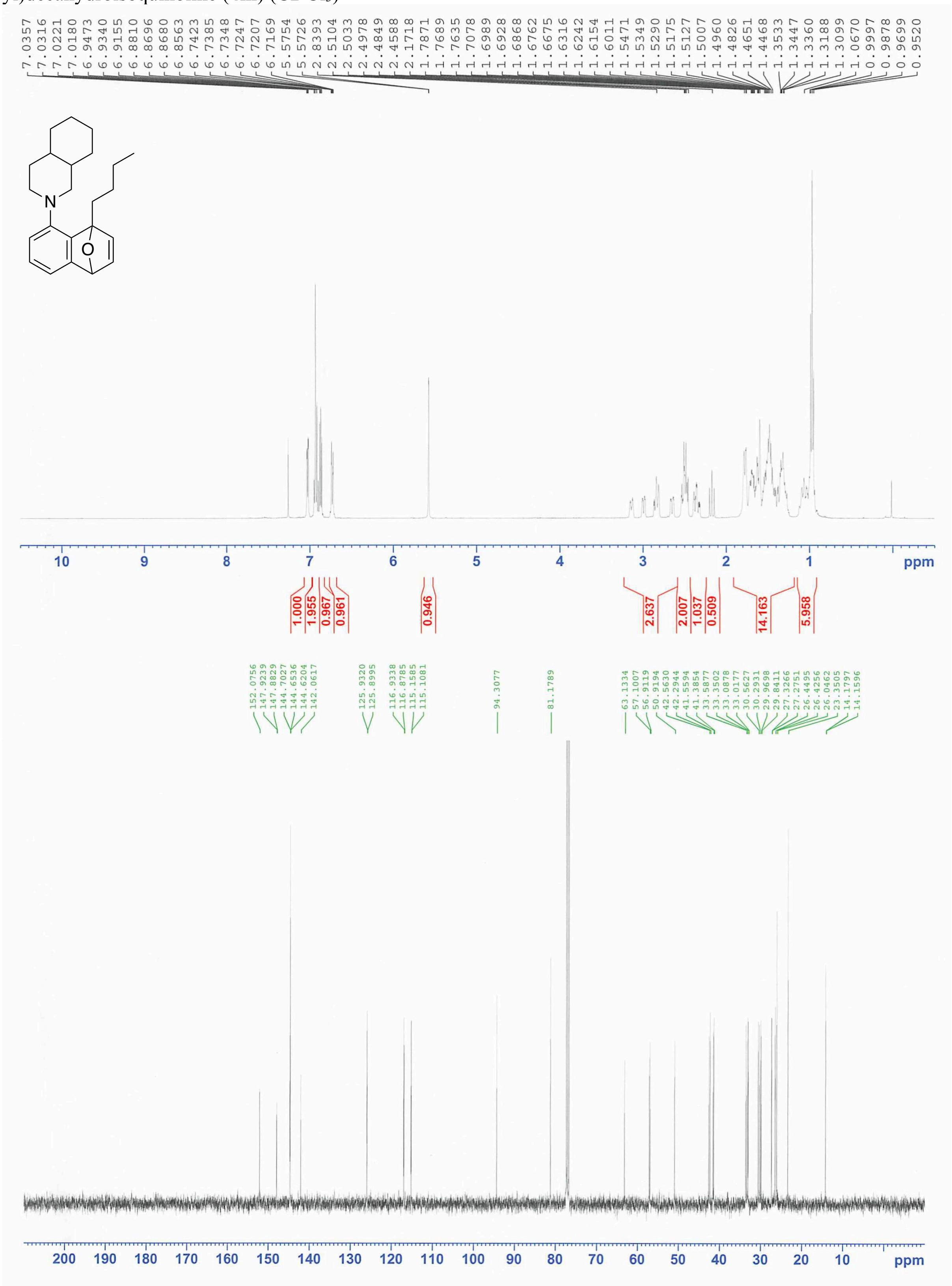

^{1}H NMR (400 MHz) and ^{13}C NMR (101 MHz) spectra of 2-(1-butyl-1,4-dihydro-1,4-epoxynaphthalen-5-yl)decahydroisoquinoline (**5m**) ($CDCl_3$)

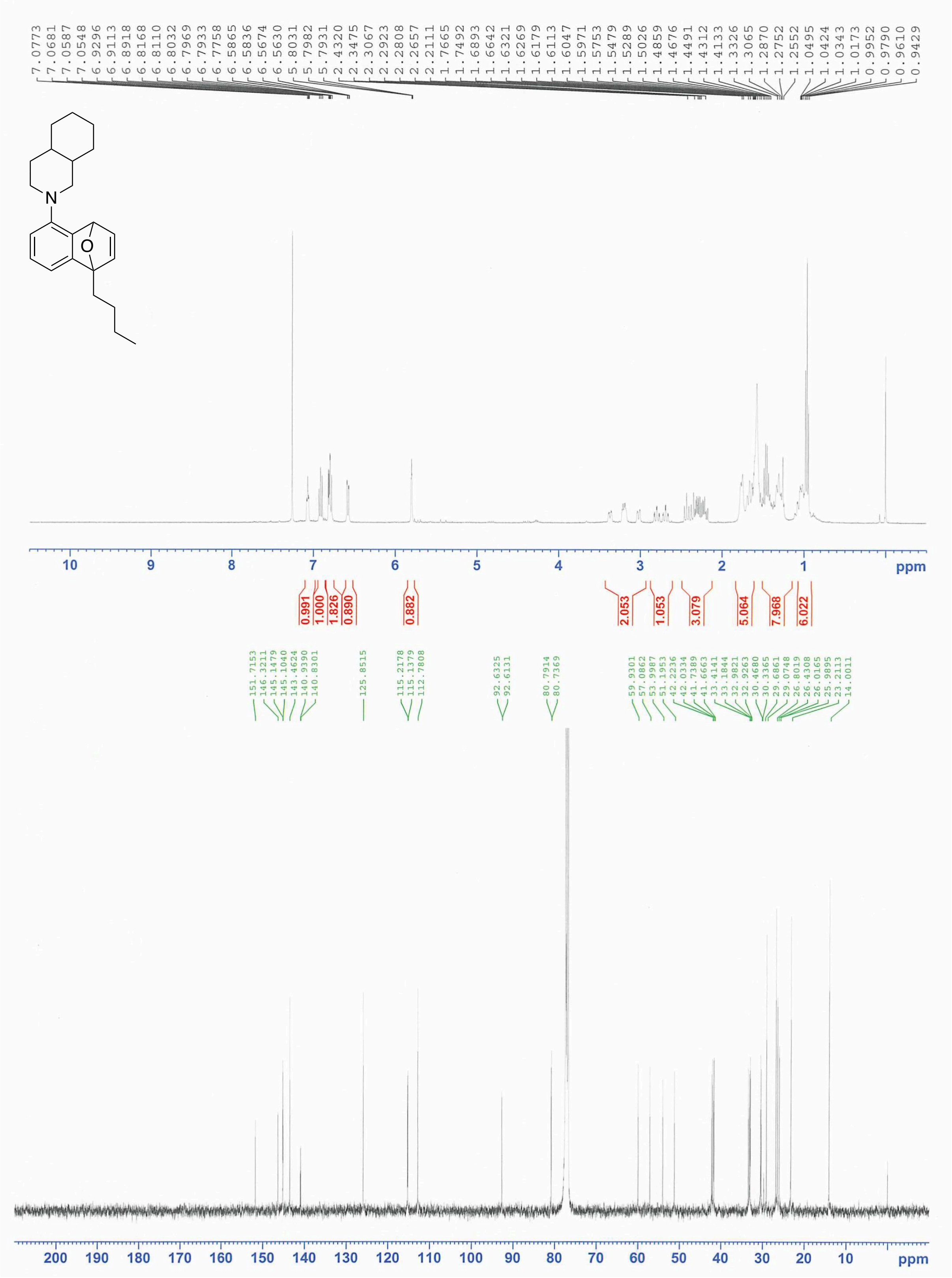

^{1}H NMR (400 MHz) and ^{13}C NMR (101 MHz) spectra of methyl 6-methyl-8-morpholino-1,4-epoxynaphthalene-1(4*H*)-carboxylate (**4n**) ($CDCl_3$)

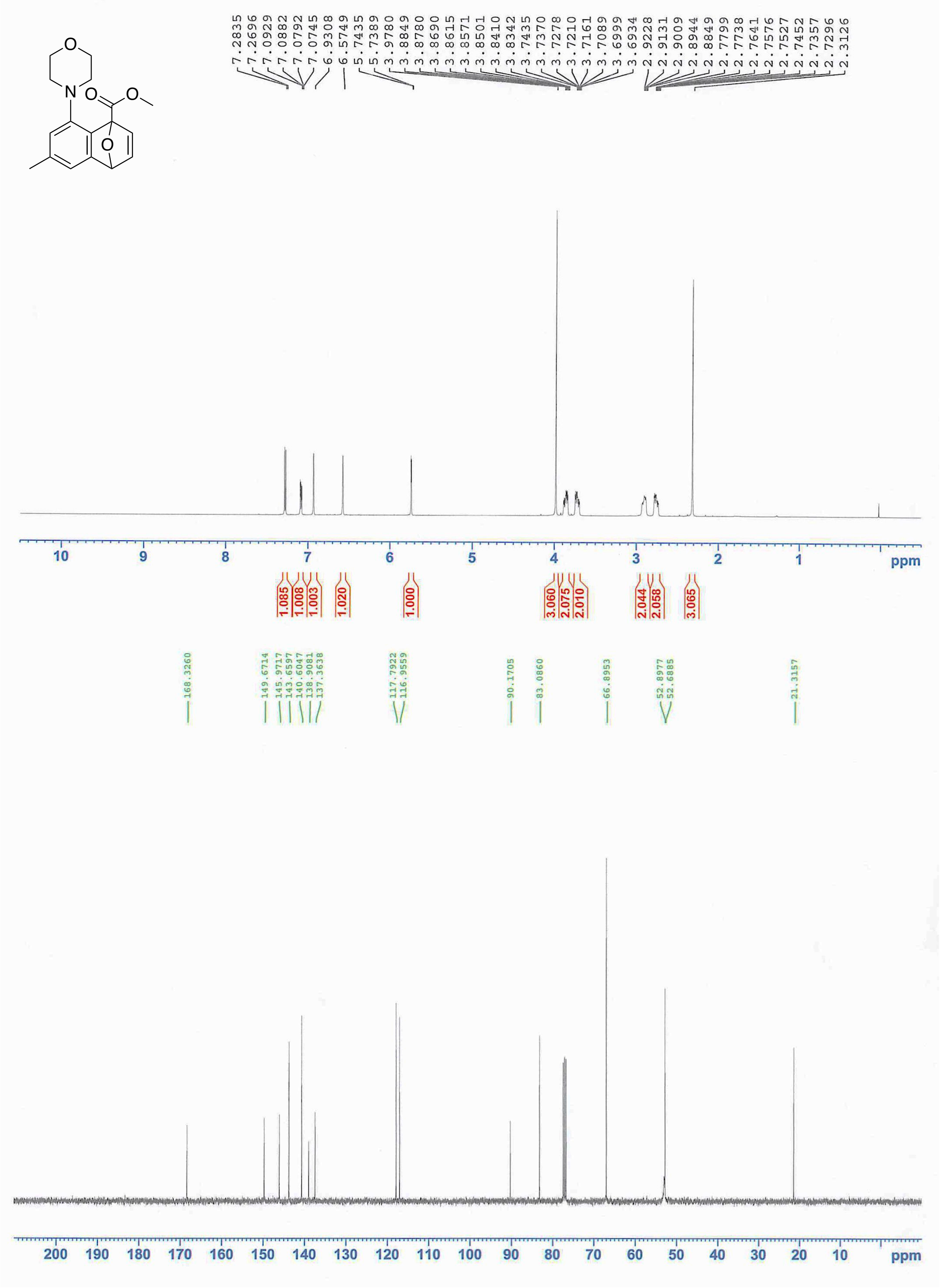

^{1}H NMR (400 MHz) and ^{13}C NMR (101 MHz) spectra of methyl 7-methyl-5-morpholino-1,4-epoxynaphthalene-1(4*H*)-carboxylate (**5n**) ($CDCl_3$)

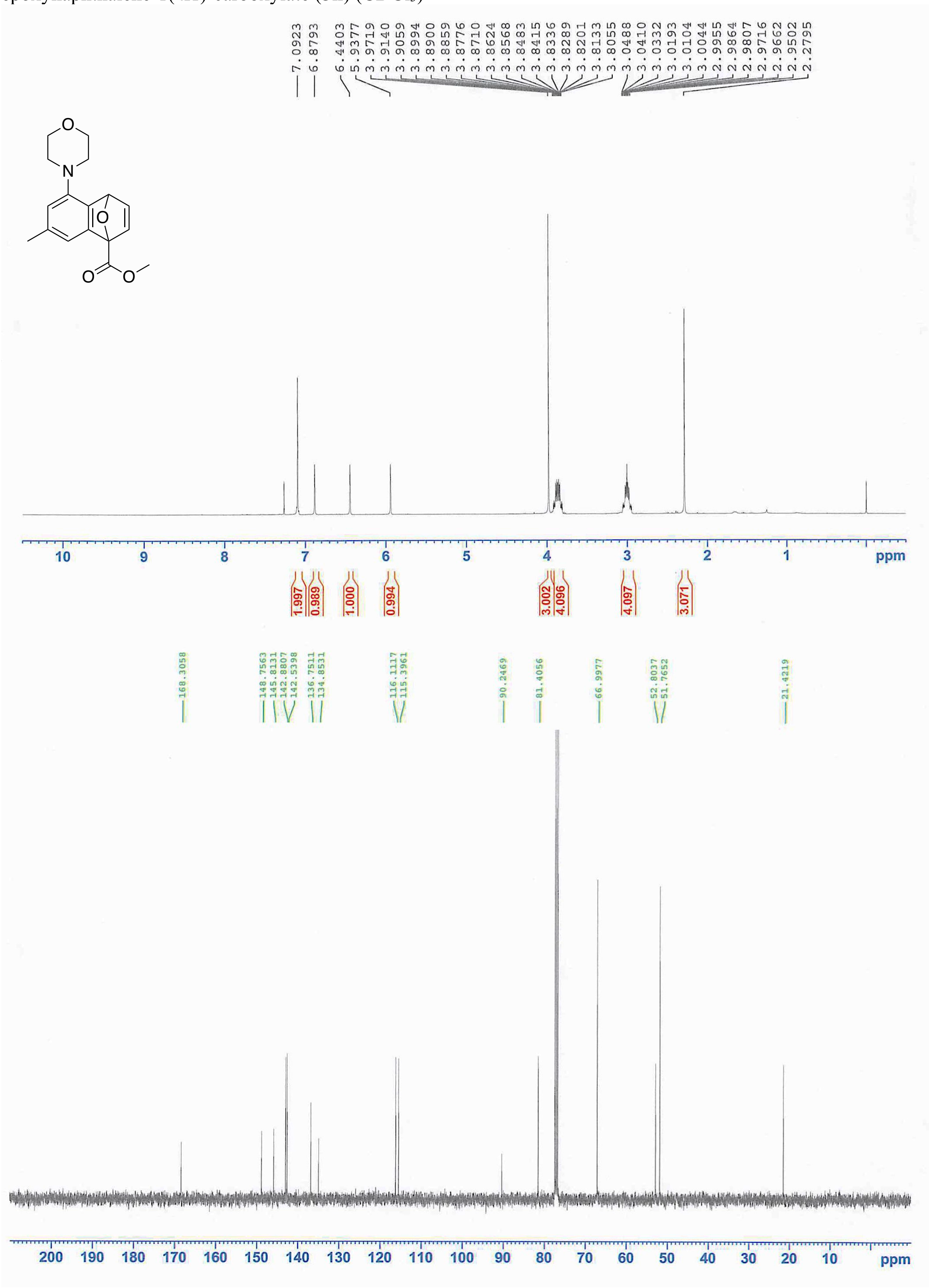

^{1}H NMR (400 MHz) and ^{13}C NMR (101 MHz) spectra of 4-(4-butyl-7-methyl-1,4-dihydro-1,4-epoxynaphthalen-5-yl)morpholine (**4o**) ($CDCl_3$)

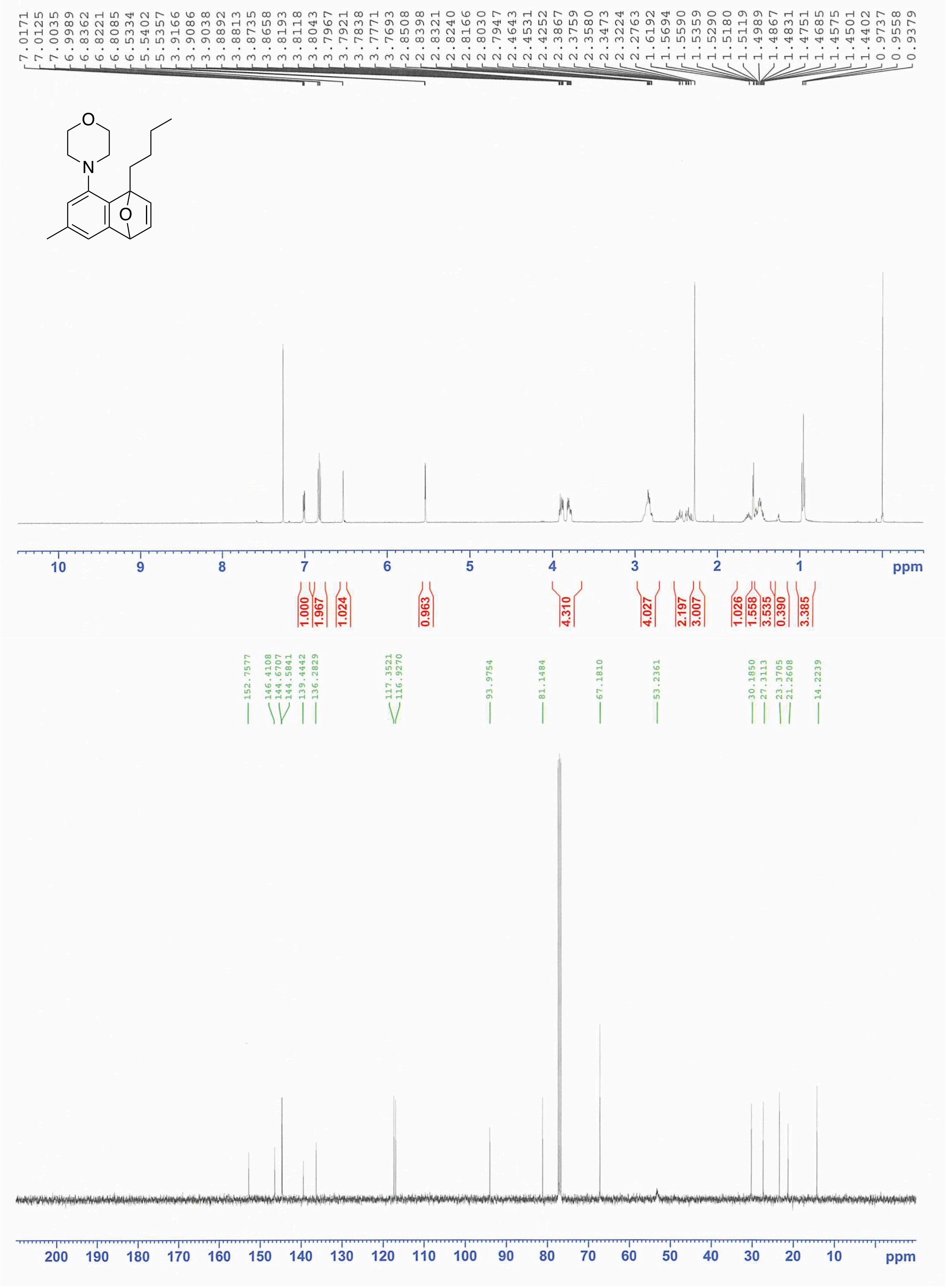

^{1}H NMR (400 MHz) and ^{13}C NMR (101 MHz) spectra of 4-(1-butyl-7-methyl-1,4-dihydro-1,4-epoxynaphthalen-5-yl)morpholine (**5o**) ($CDCl_3$)

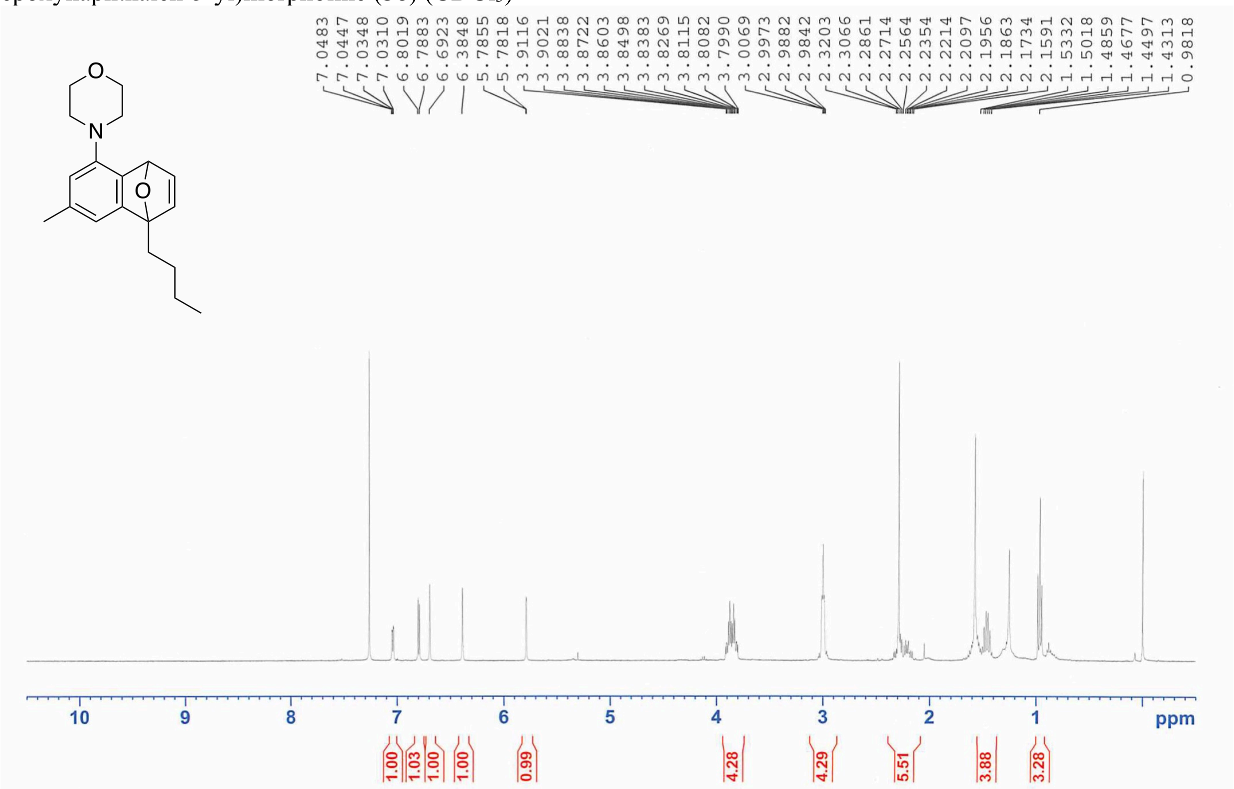

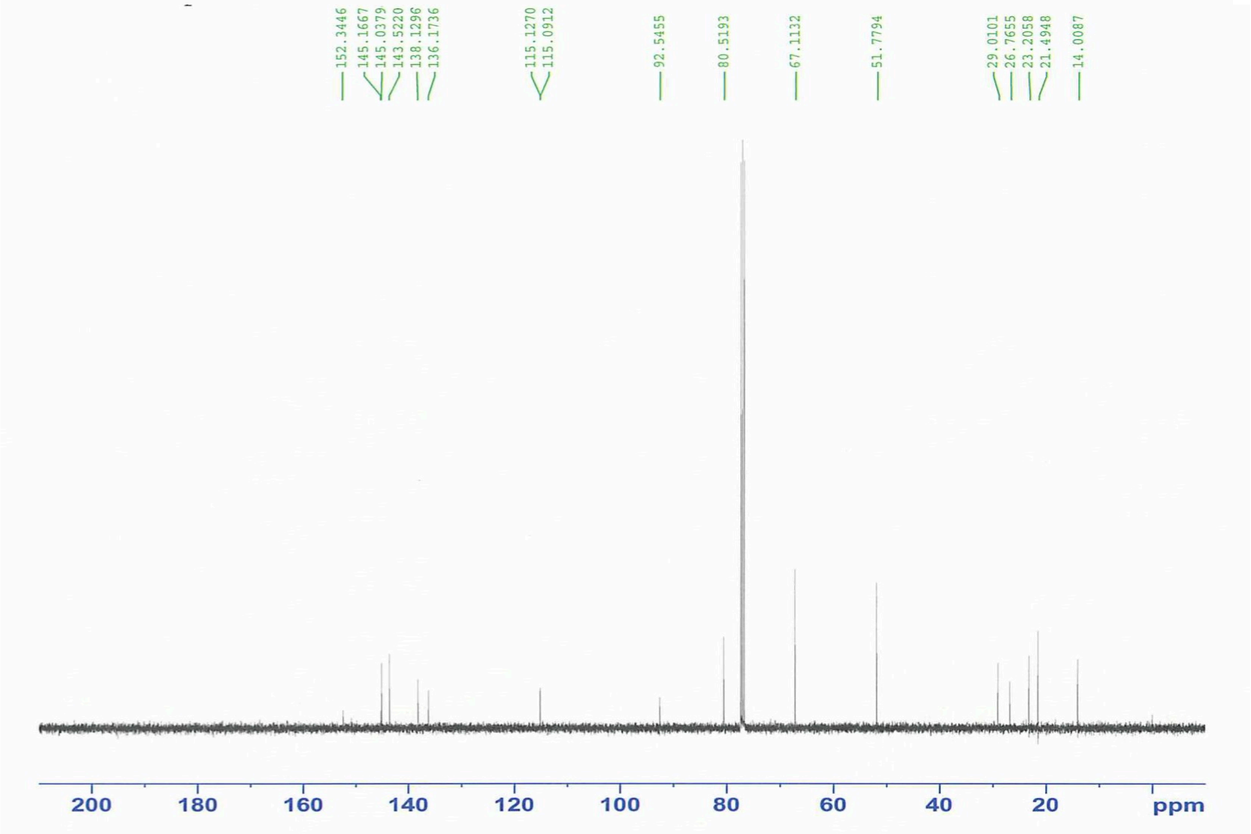

^{1}H NMR (400 MHz) and ^{13}C NMR (101 MHz) spectra of methyl 6-bromo-8-morpholino-1,4-epoxynaphthalene-1(4*H*)-carboxylate (**4p**) ($CDCl_3$)

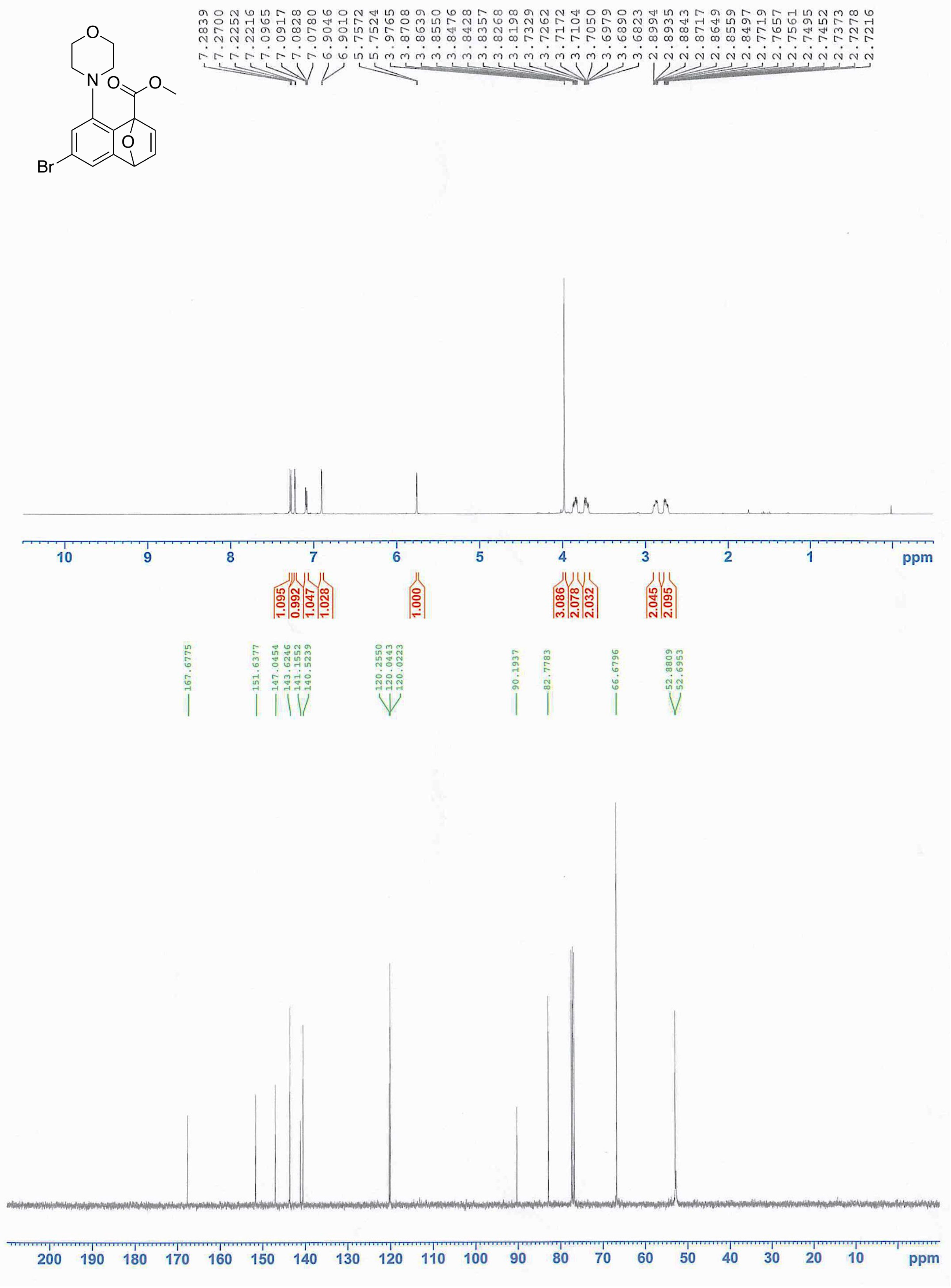

^{1}H NMR (400 MHz) and ^{13}C NMR (101 MHz) spectra of methyl 7-bromo-5-morpholino-1,4-epoxynaphthalene-1(4*H*)-carboxylate (**5p**) ($CDCl_3$)

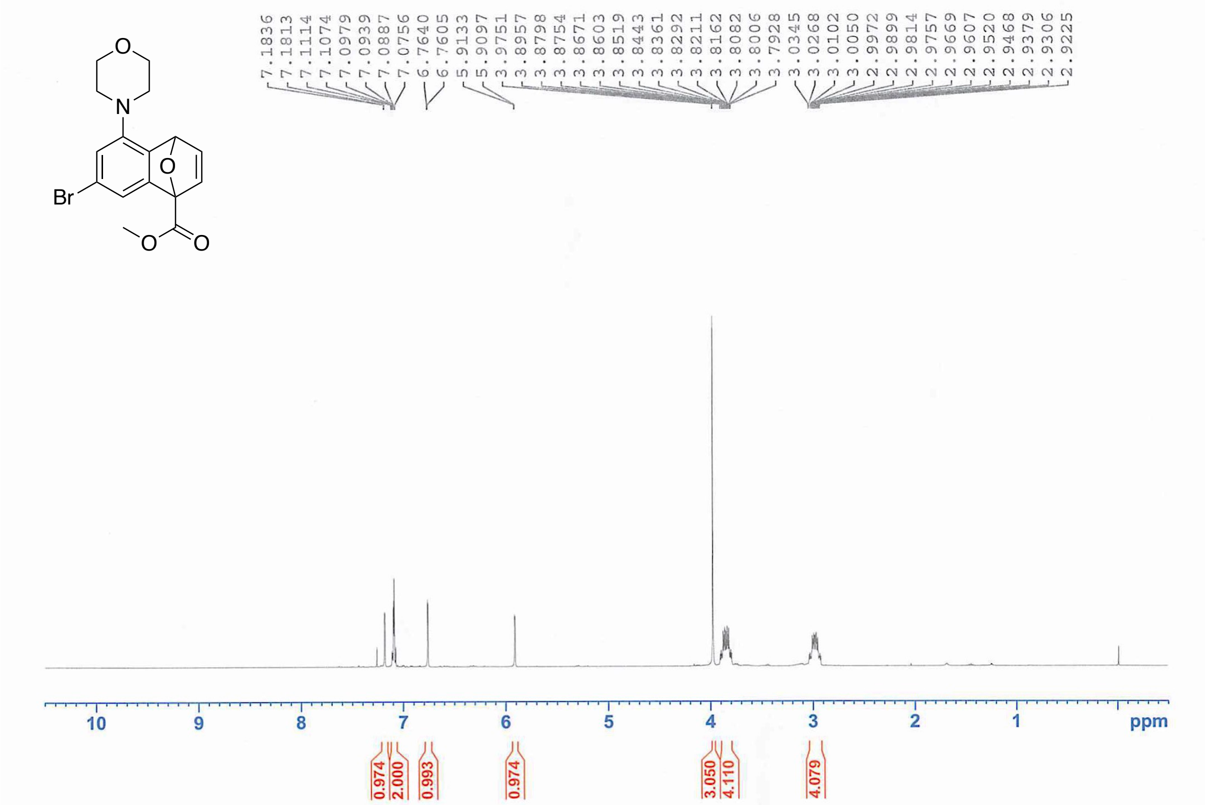

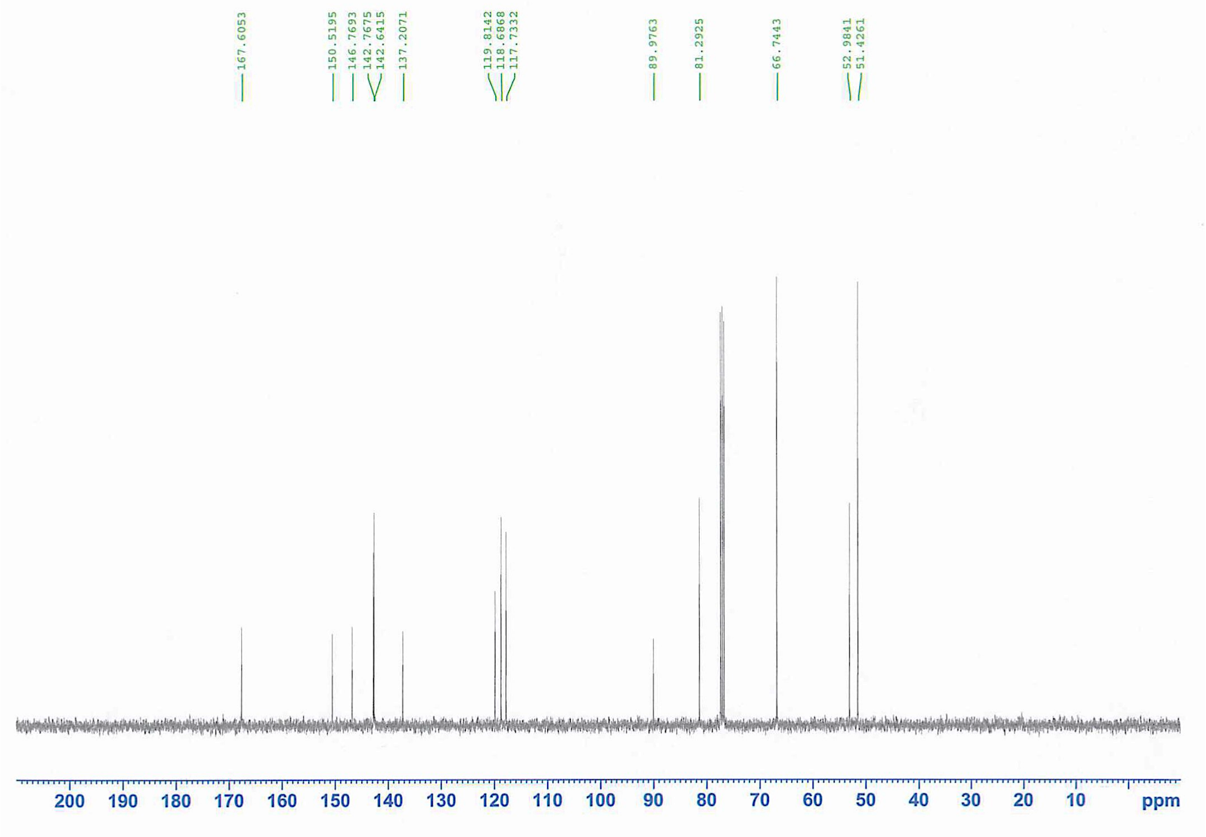

^{1}H NMR (400 MHz) and ^{13}C NMR (101 MHz) spectra of 4-(7-bromo-4-butyl-1,4-dihydro-1,4-epoxynaphthalen-5-yl)morpholine (**4q**) ($CDCl_3$)

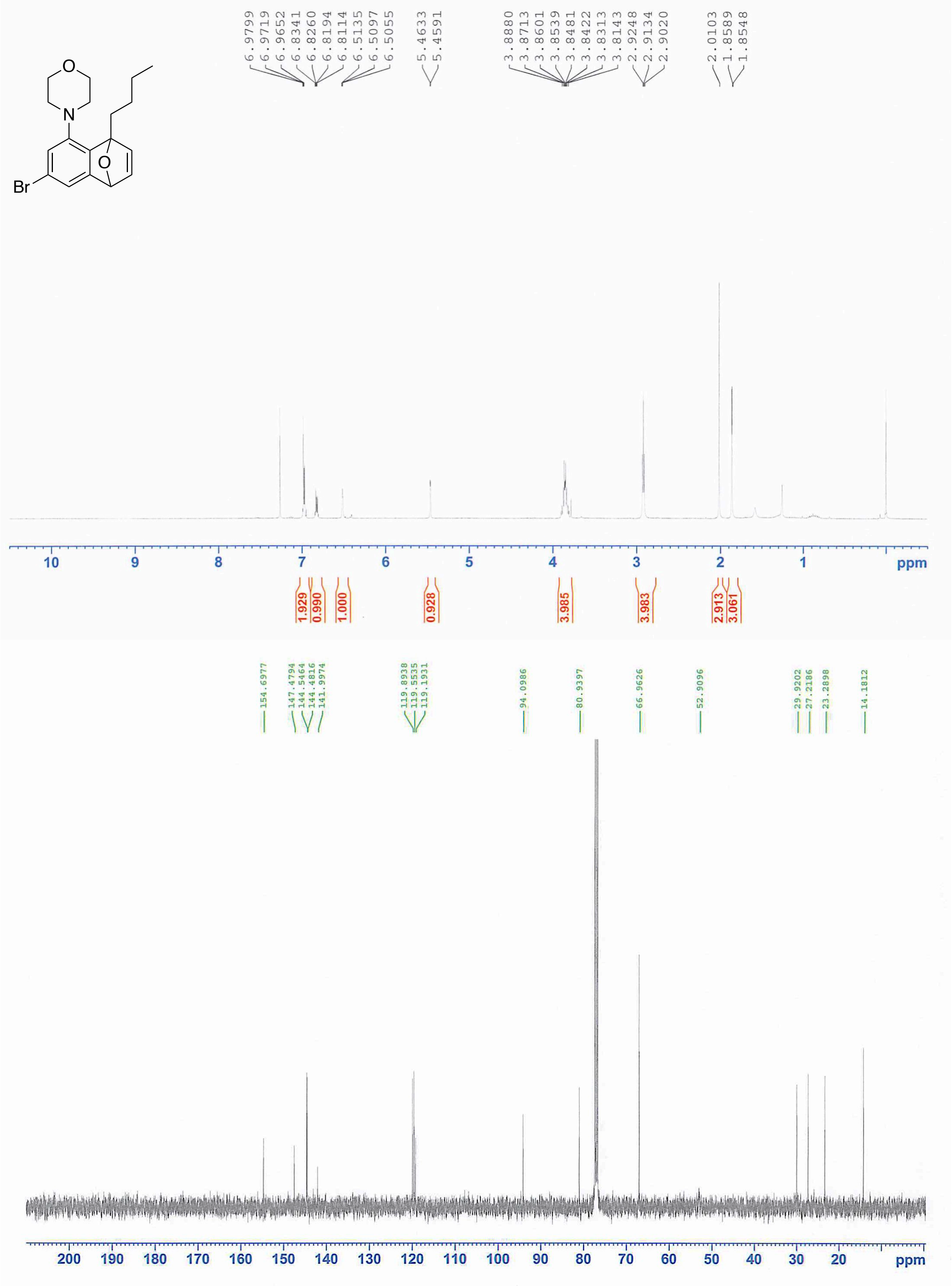

^{1}H NMR (400 MHz) and ^{13}C NMR (101 MHz) spectra of 4-(7-bromo-1-butyl-1,4-dihydro-1,4-epoxynaphthalen-5-yl)morpholine (**5q**) ($CDCl_3$)

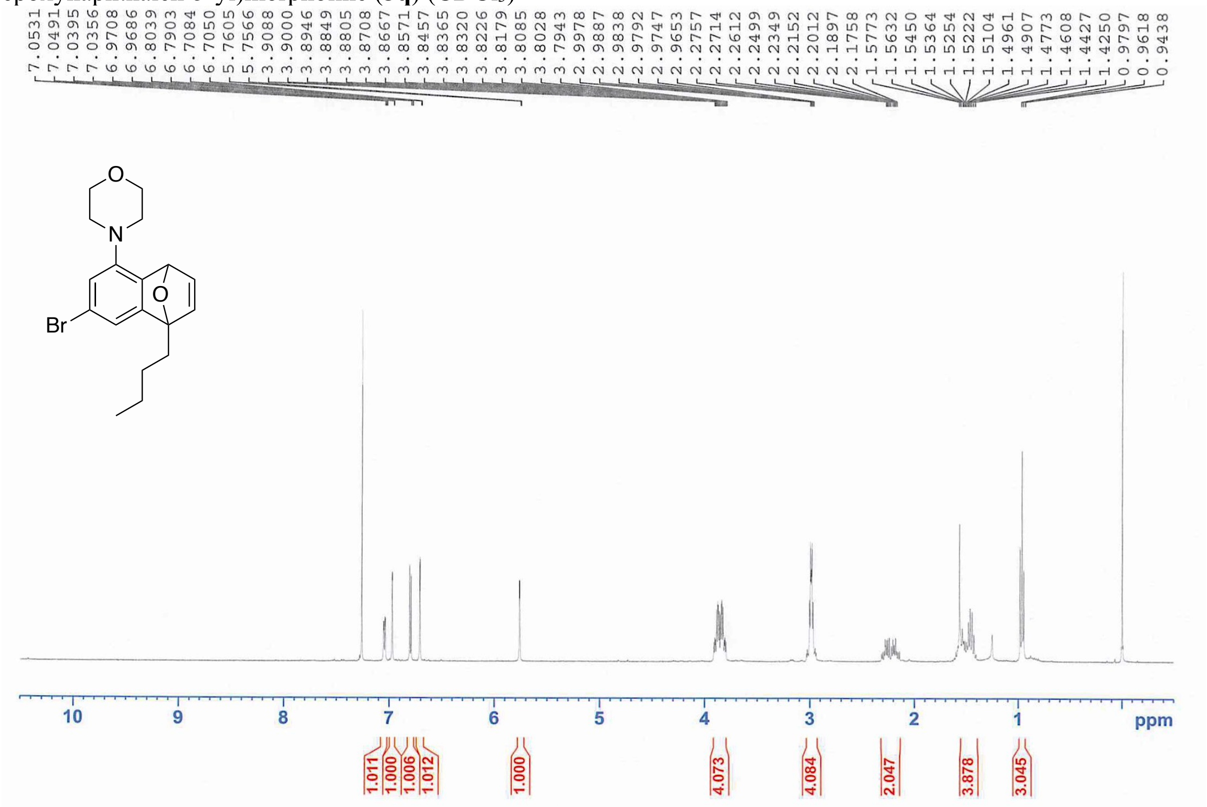

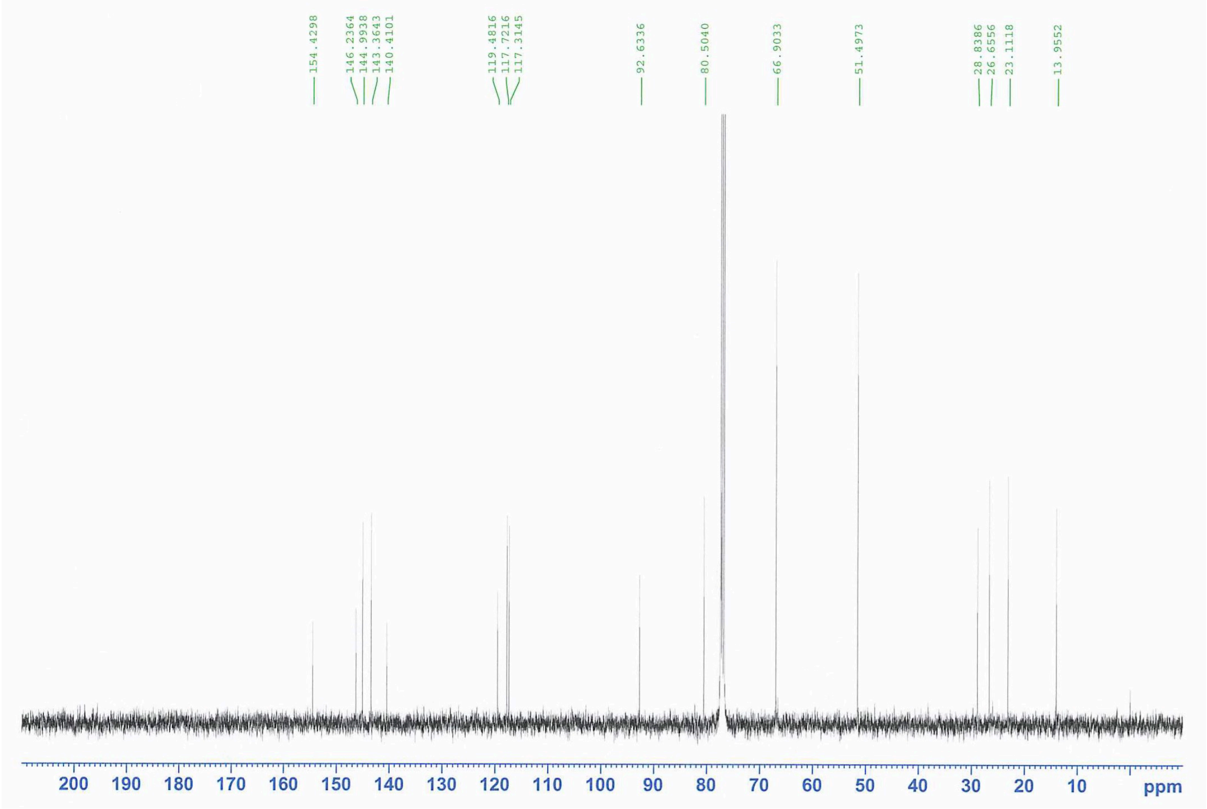

^{1}H NMR (400 MHz) and ^{13}C NMR (101 MHz) spectra of a mixture of 1-butyl-8-methoxy-1,4-dihydro-1,4-epoxynaphthalene (**9b**) and 1-butyl-5-methoxy-1,4-dihydro-1,4-epoxynaphthalene (**10b**) ($CDCl_3$)

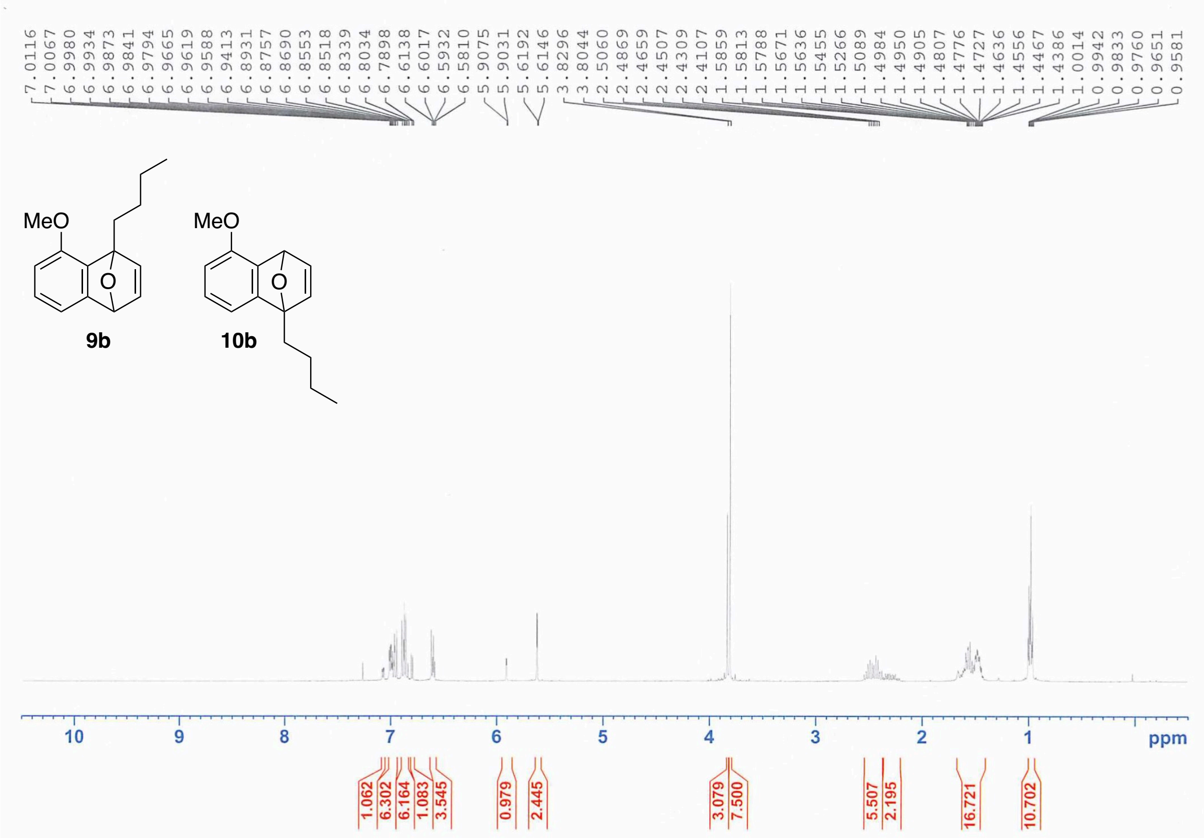

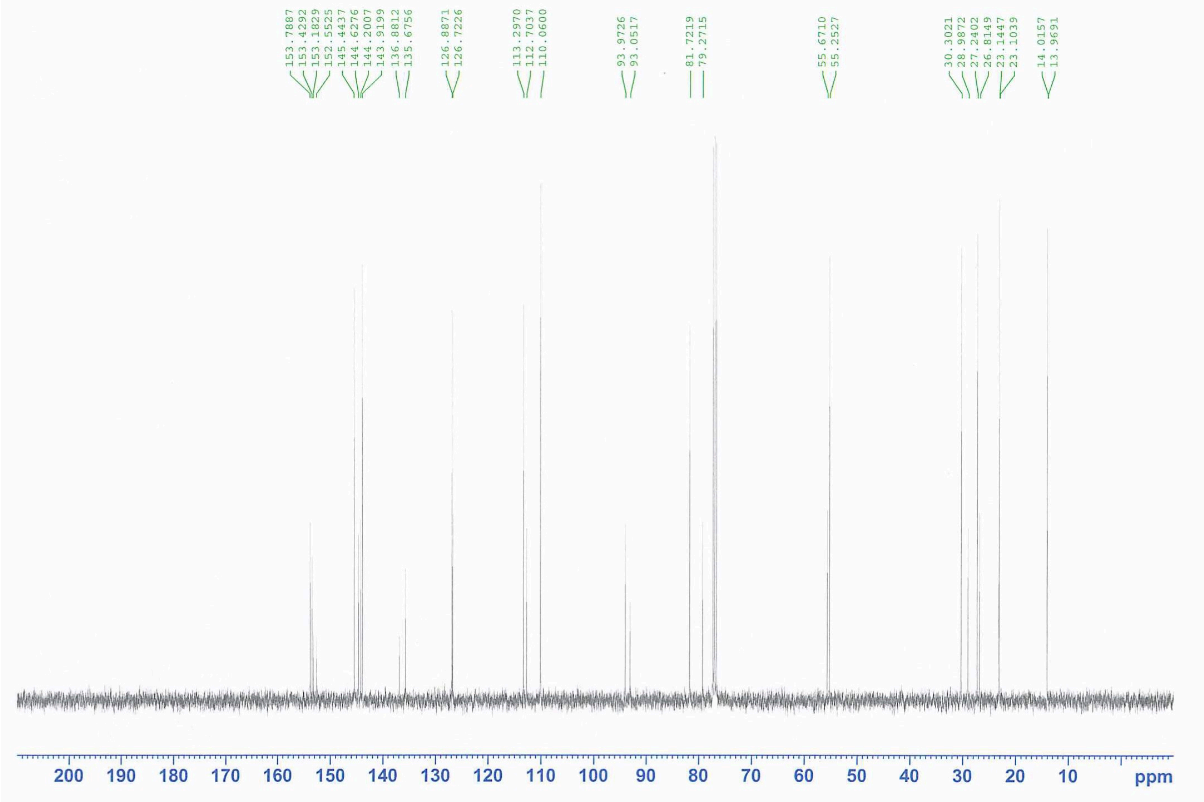

^{1}H NMR (400 MHz) and ^{13}C NMR (101 MHz) spectra of 4-(5,6,7,8-tetraphenylnaphthalen-1-yl)morpholine (**7a**) ($CDCl_3$)

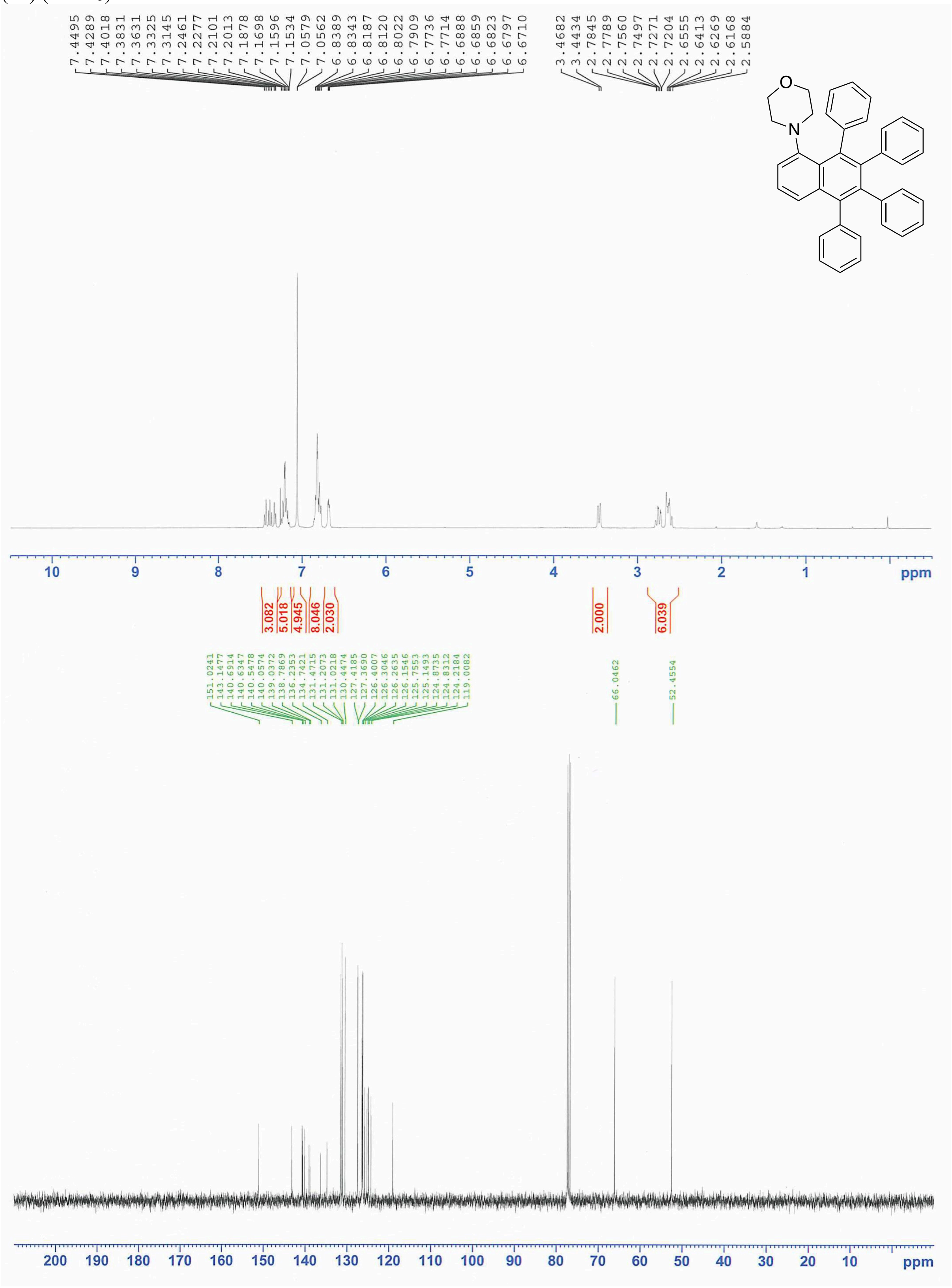

^{1}H NMR (400 MHz) and ^{13}C NMR (101 MHz) spectra of 4-methoxy-5-morpholinonaphthalen-1-ol (**7b**) ($CDCl_3$)

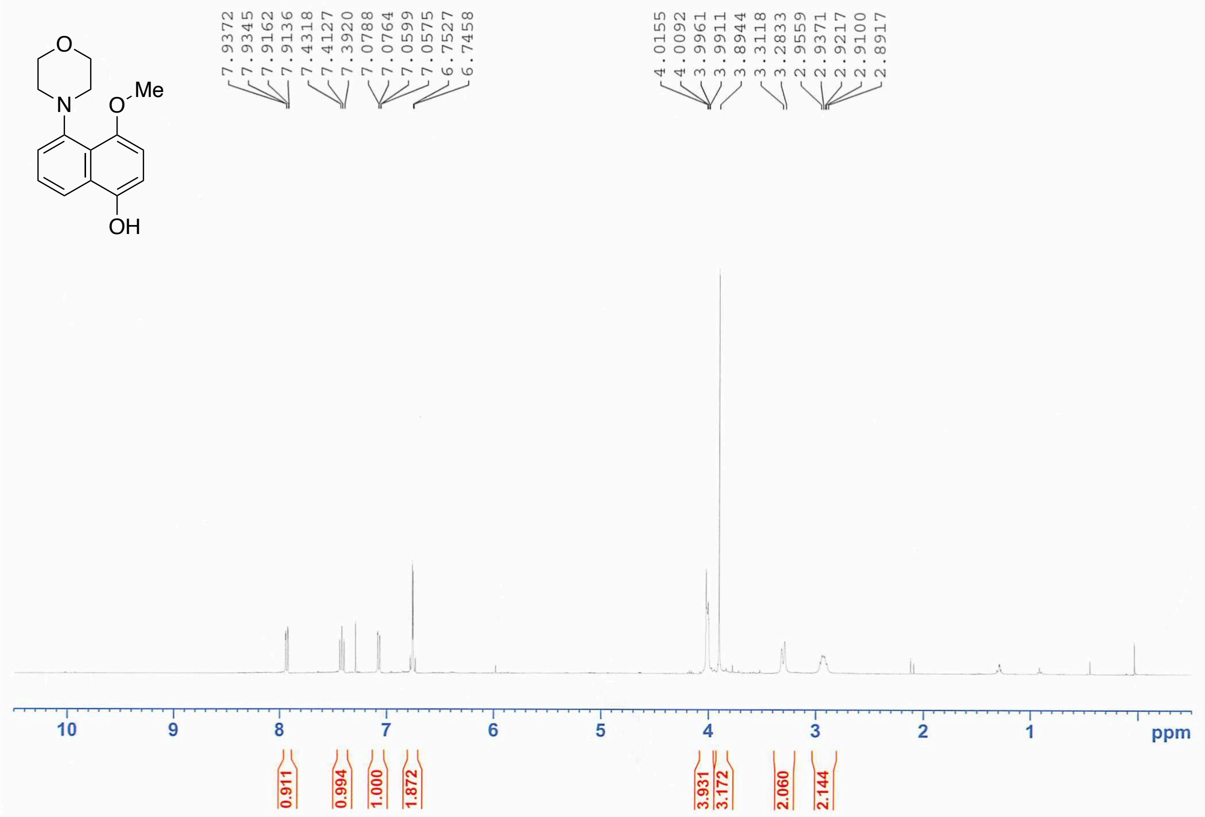

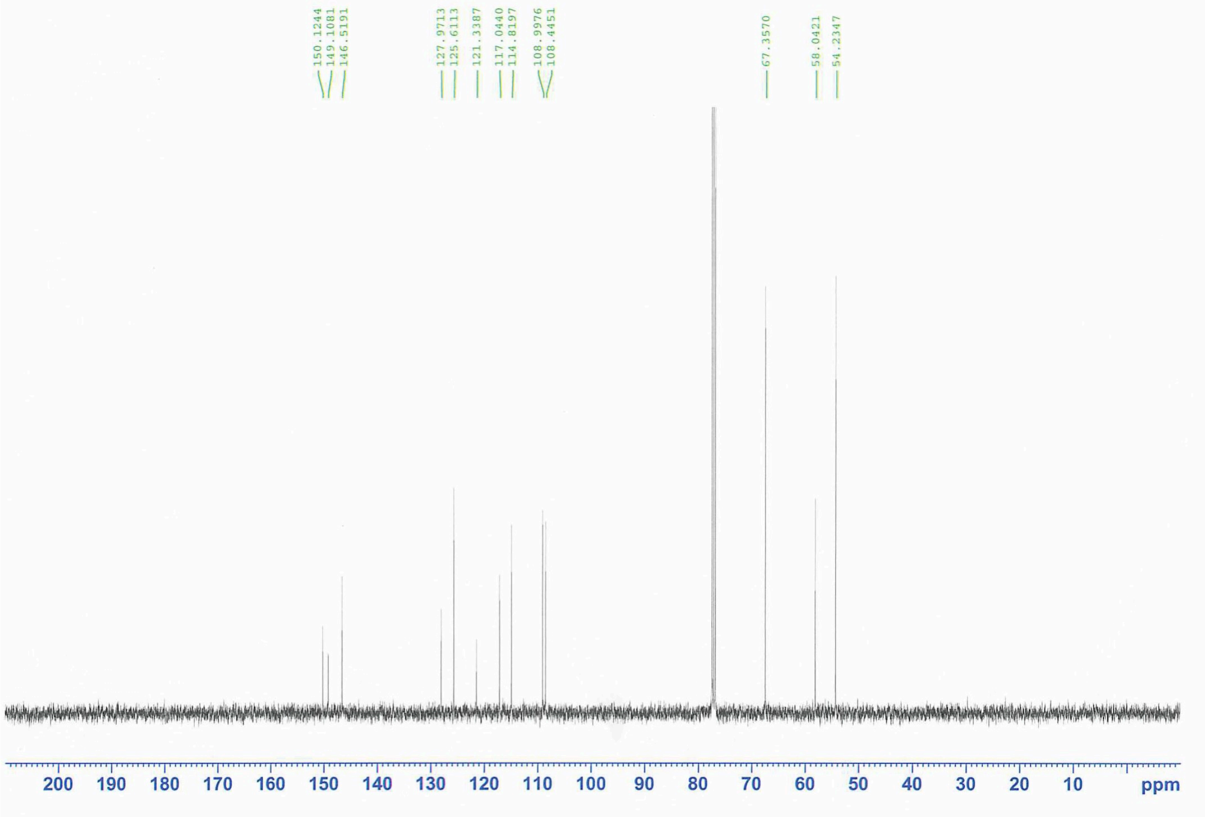

^{1}H NMR (400 MHz) and ^{13}C NMR (101 MHz) spectra of methyl 8-morpholino-1-naphthoate (**7c**) ($CDCl_3$)

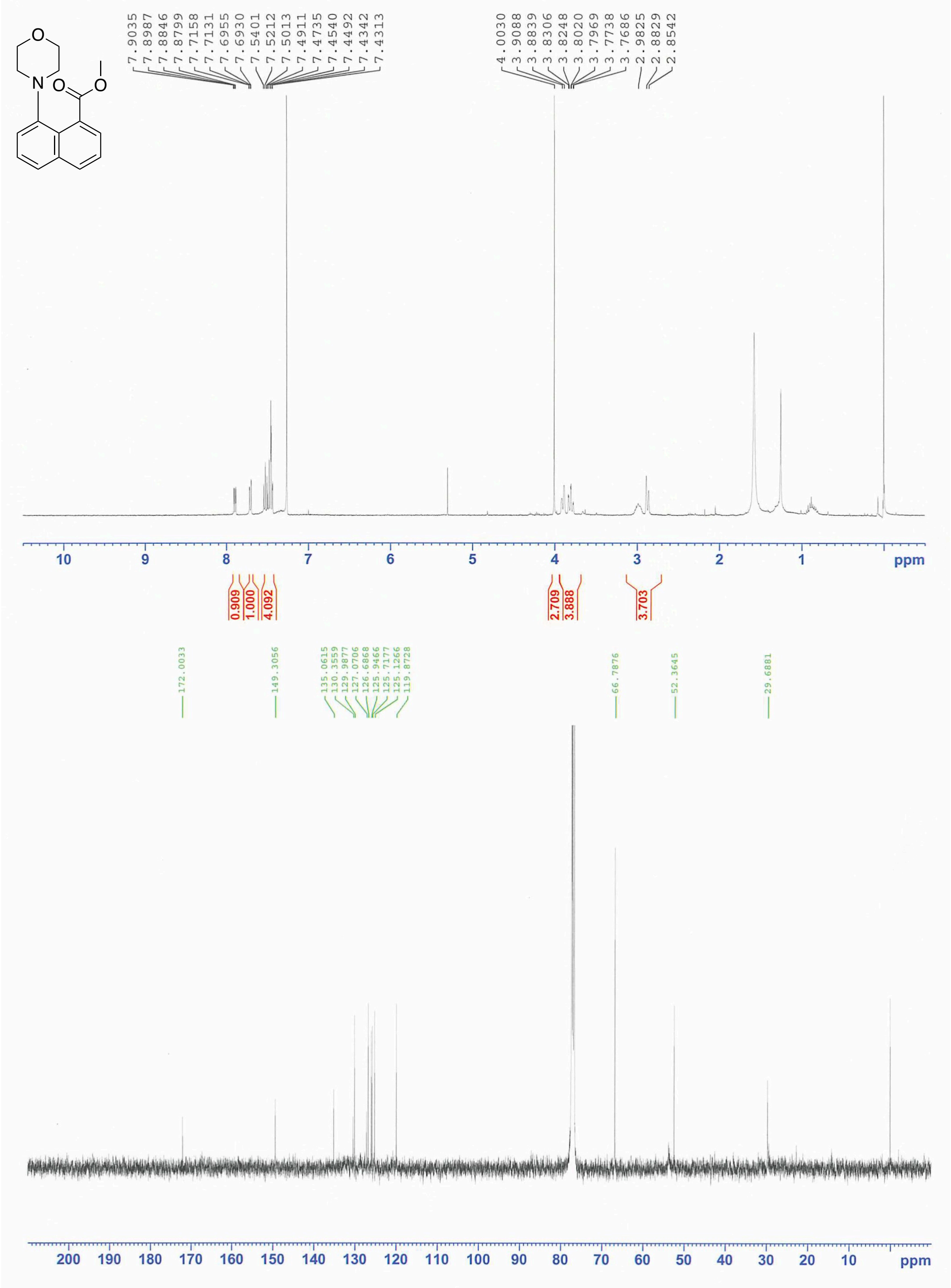

^{1}H NMR (400 MHz) and ^{13}C NMR (101 MHz) spectra of methyl 1-hydroxy-8-morpholino-2-naphthoate (**7d**) ($CDCl_3$)

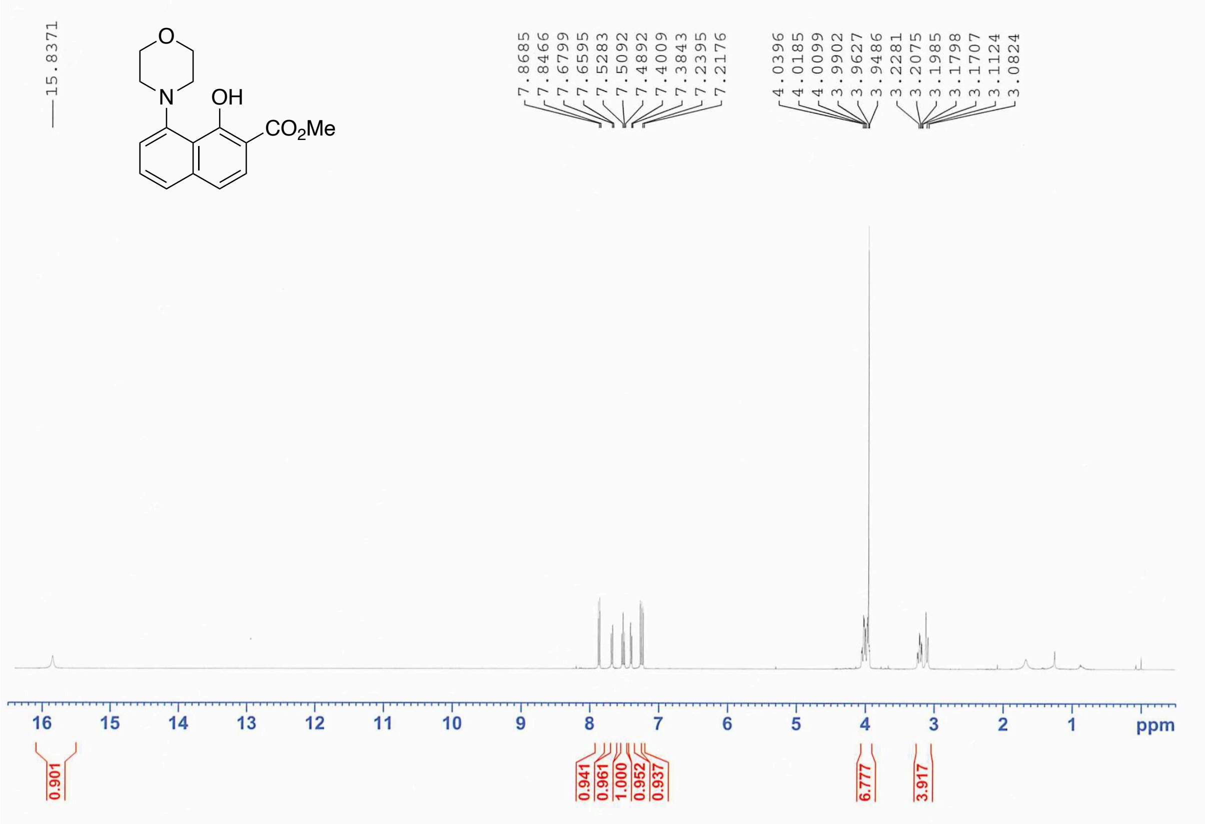

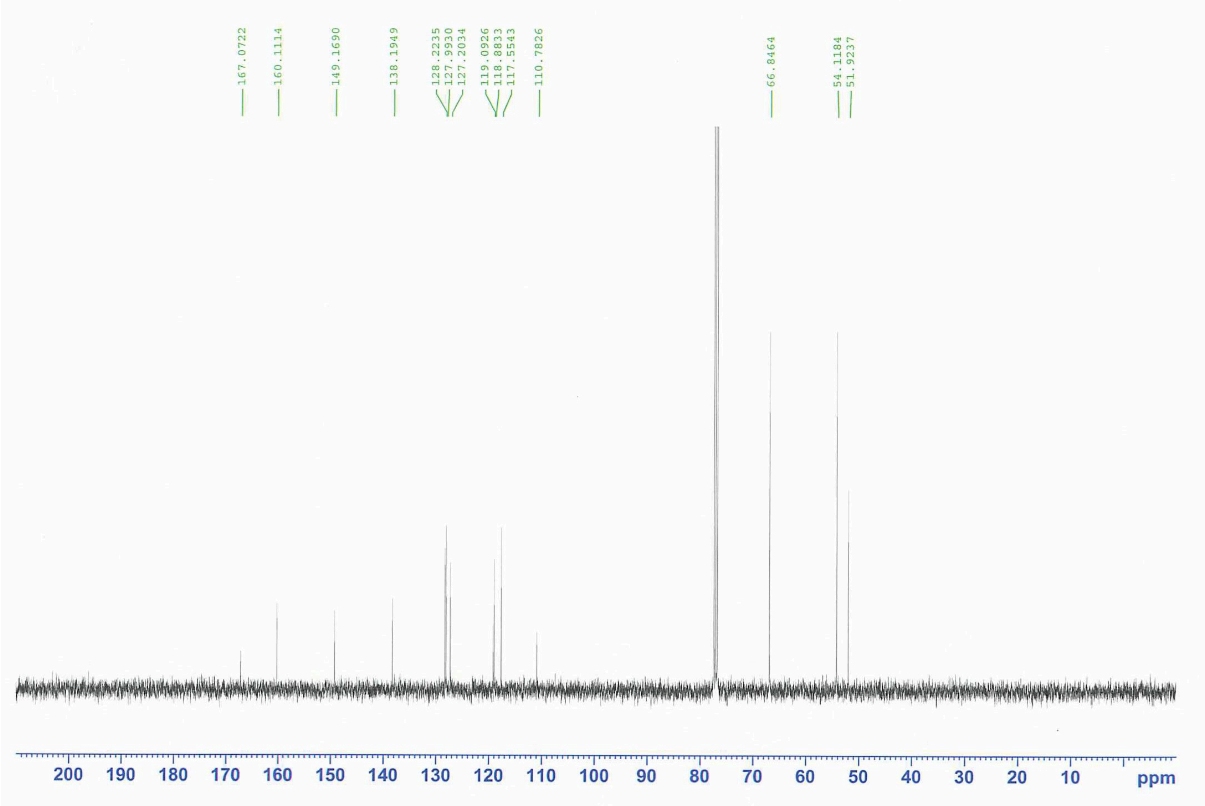

^{1}H NMR (400 MHz) and ^{13}C NMR (101 MHz) spectra of methyl 8-morpholino-3-(*p*-tolyl)-1-naphthoate (**7e**) ($CDCl_3$)

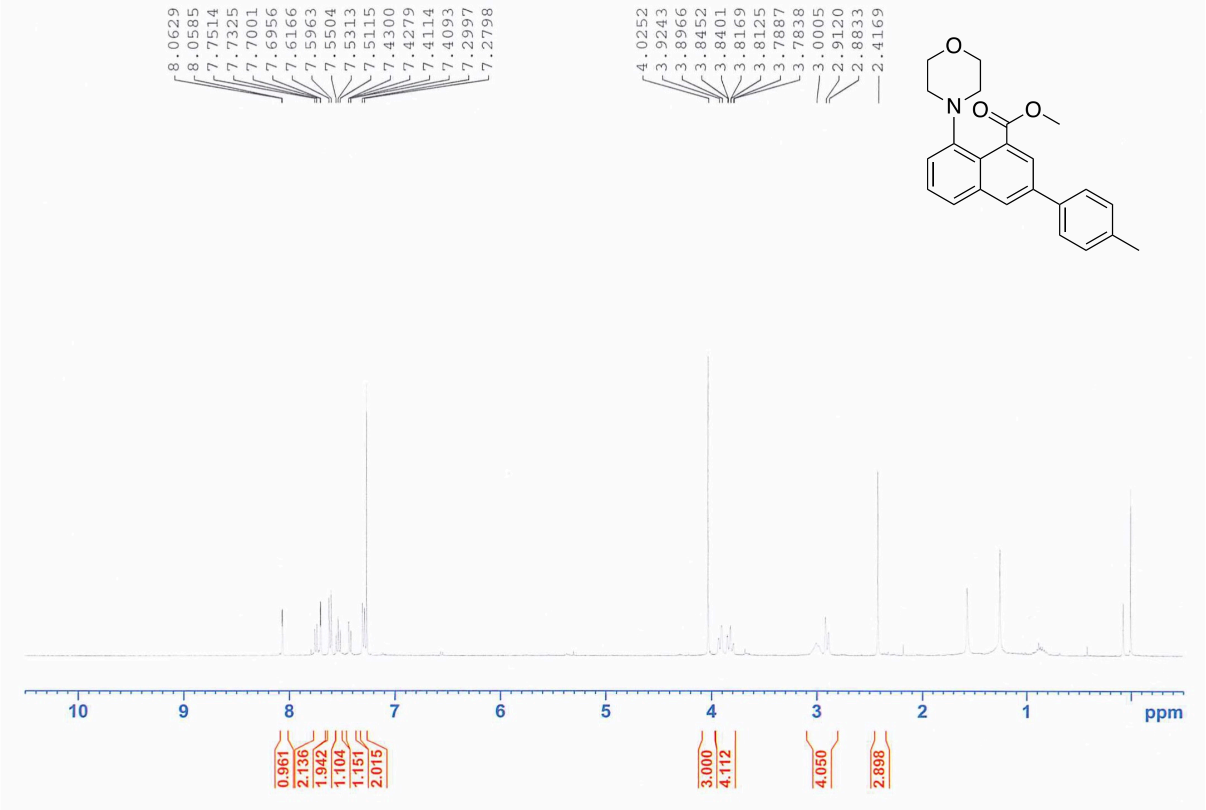

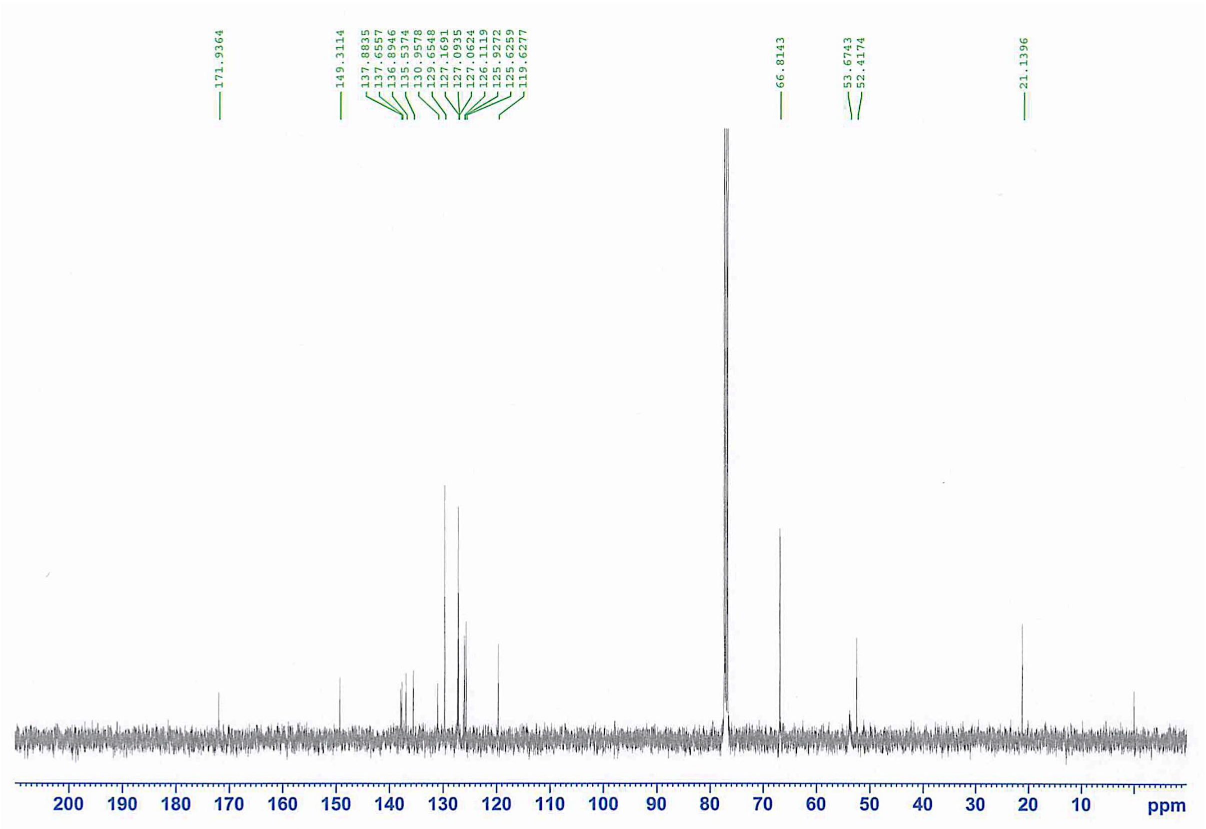

^{1}H NMR (400 MHz) and ^{13}C NMR (101 MHz) spectra of 4-(5,8-diphenylnaphthalen-1-yl)morpholine (**7f**) ($CDCl_3$)

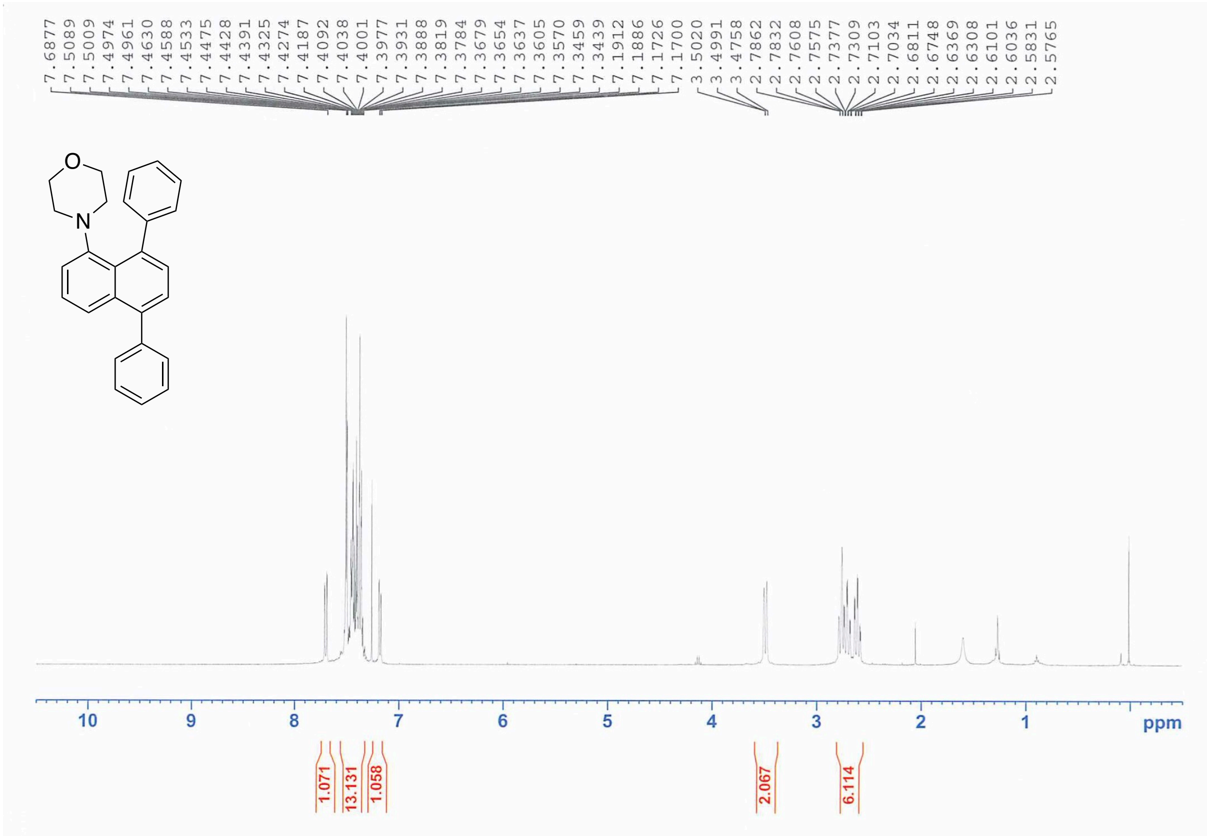

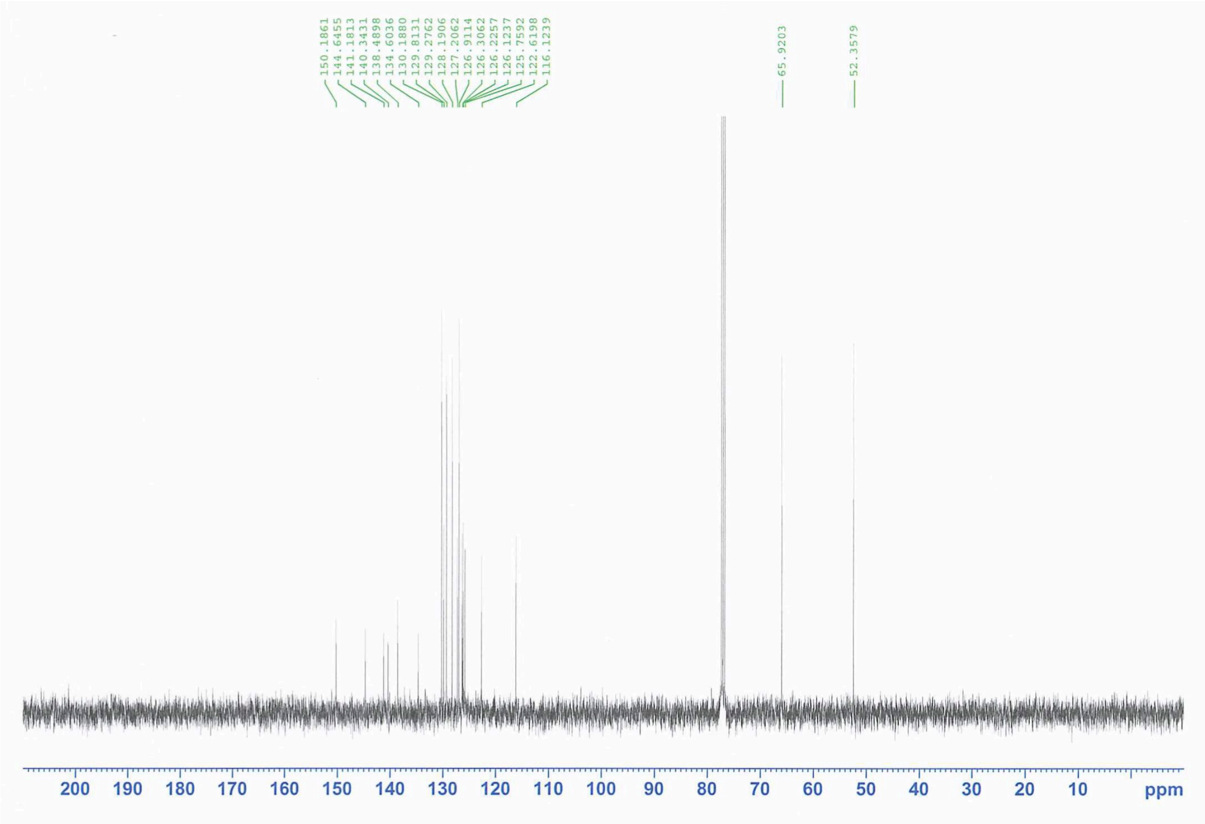

^{1}H NMR (400 MHz) and ^{13}C NMR (101 MHz) spectra of 4-(9,10-diphenylanthracen-1-yl)morpholine (**7h**) ($CDCl_3$)

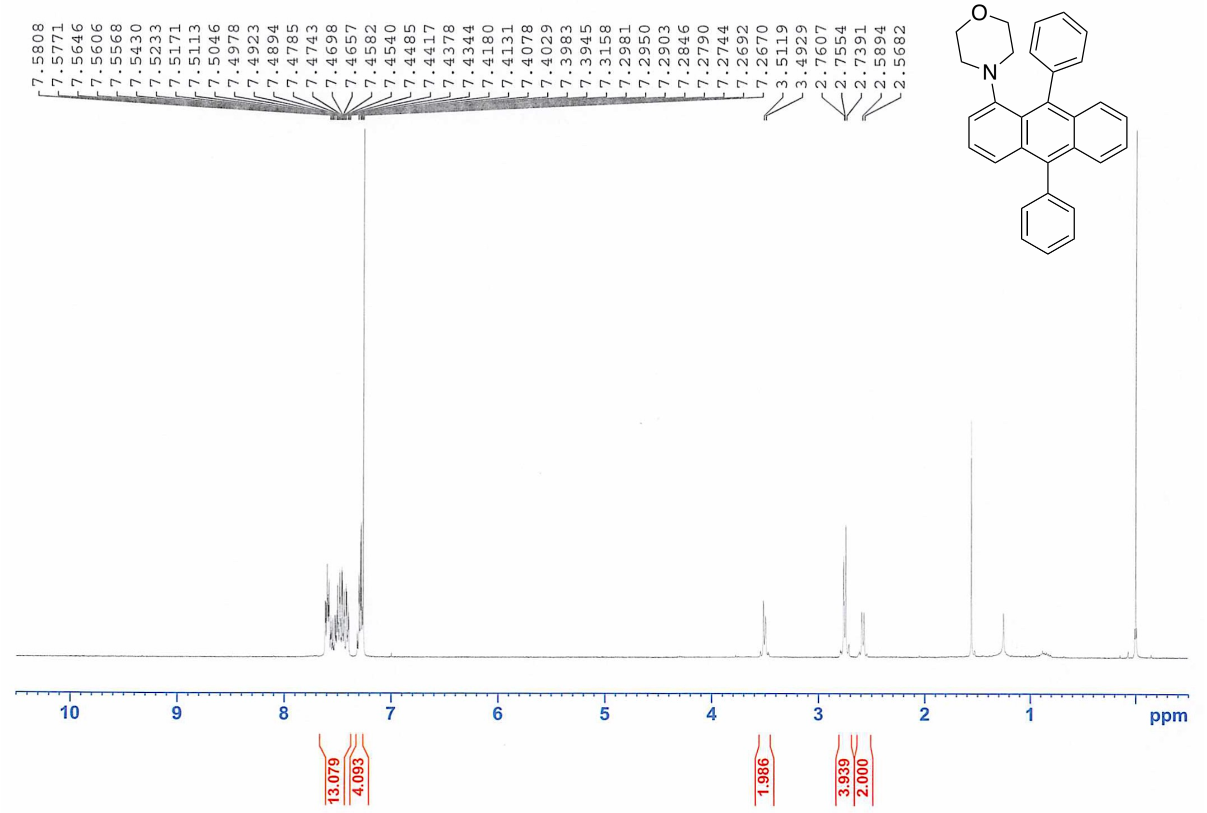

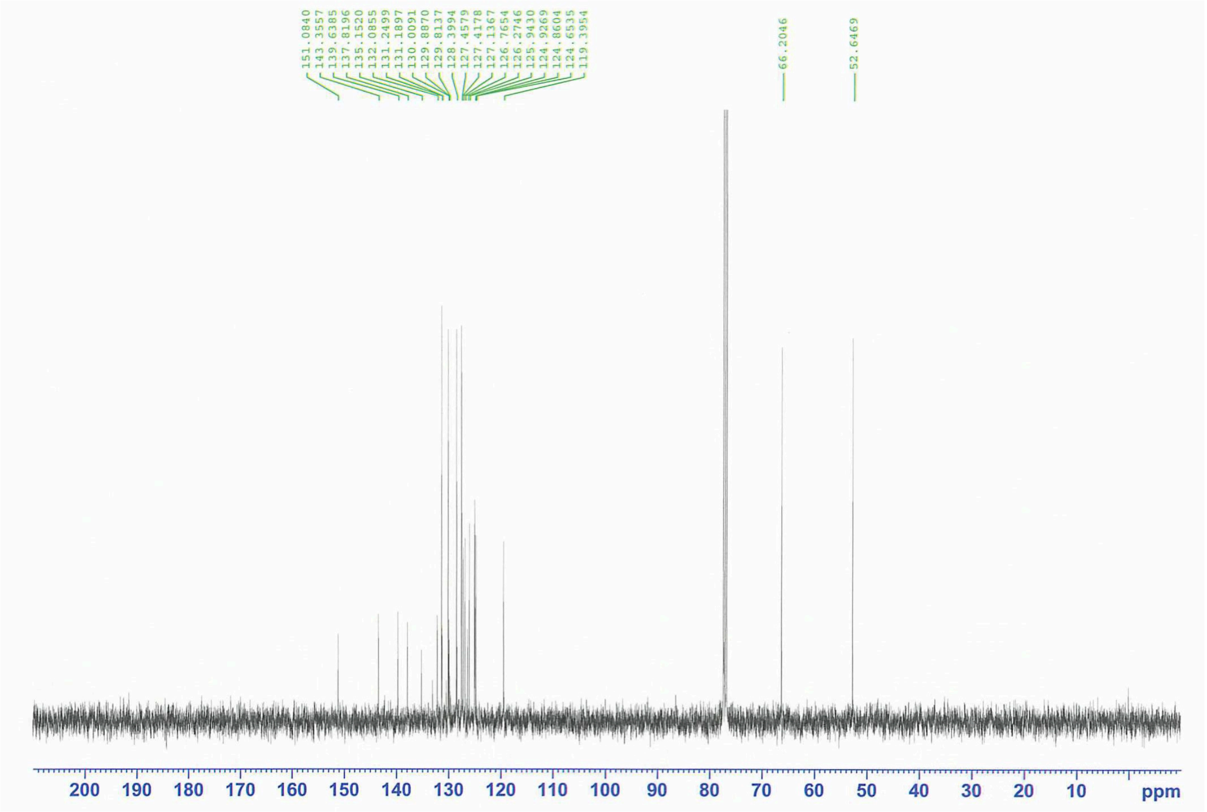

^{1}H NMR (400 MHz) and ^{13}C NMR (101 MHz) spectra of 4-(8-butylnaphthalen-1-yl)morpholine (**7i**) ($CDCl_3$)

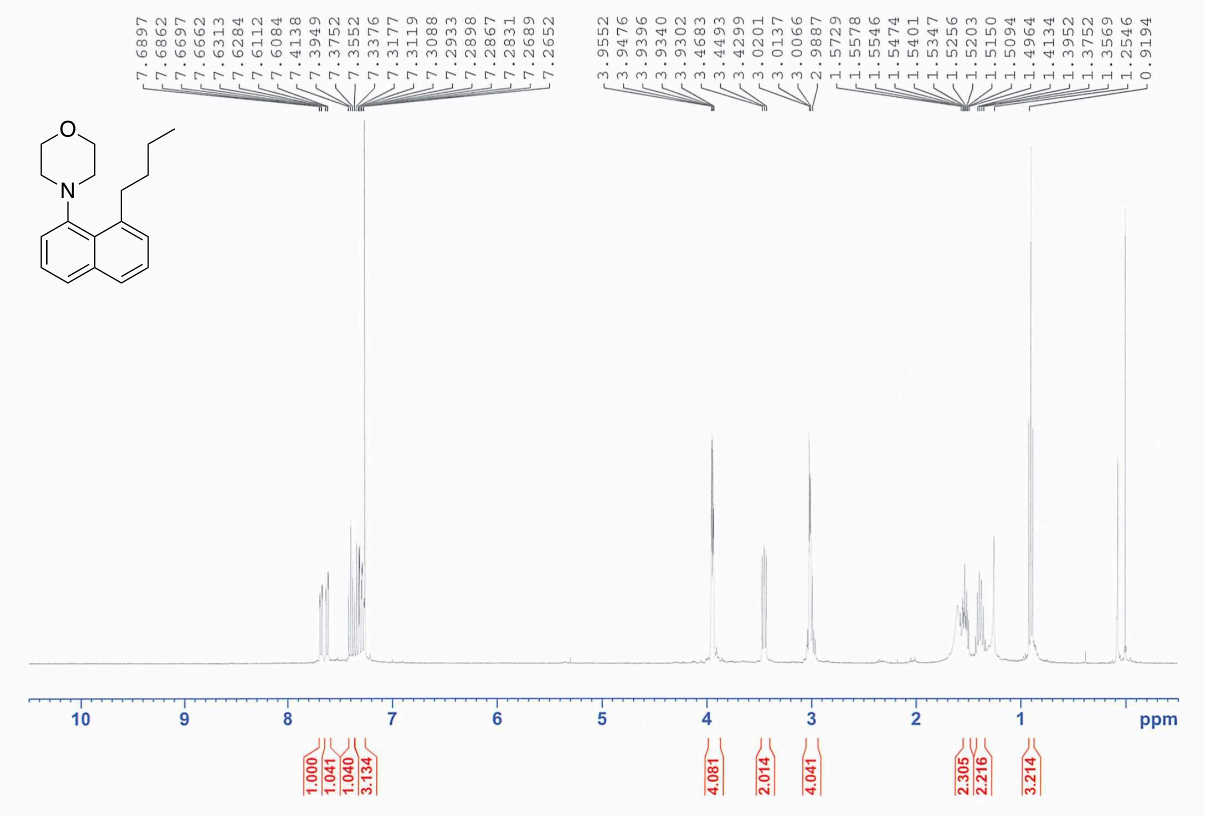

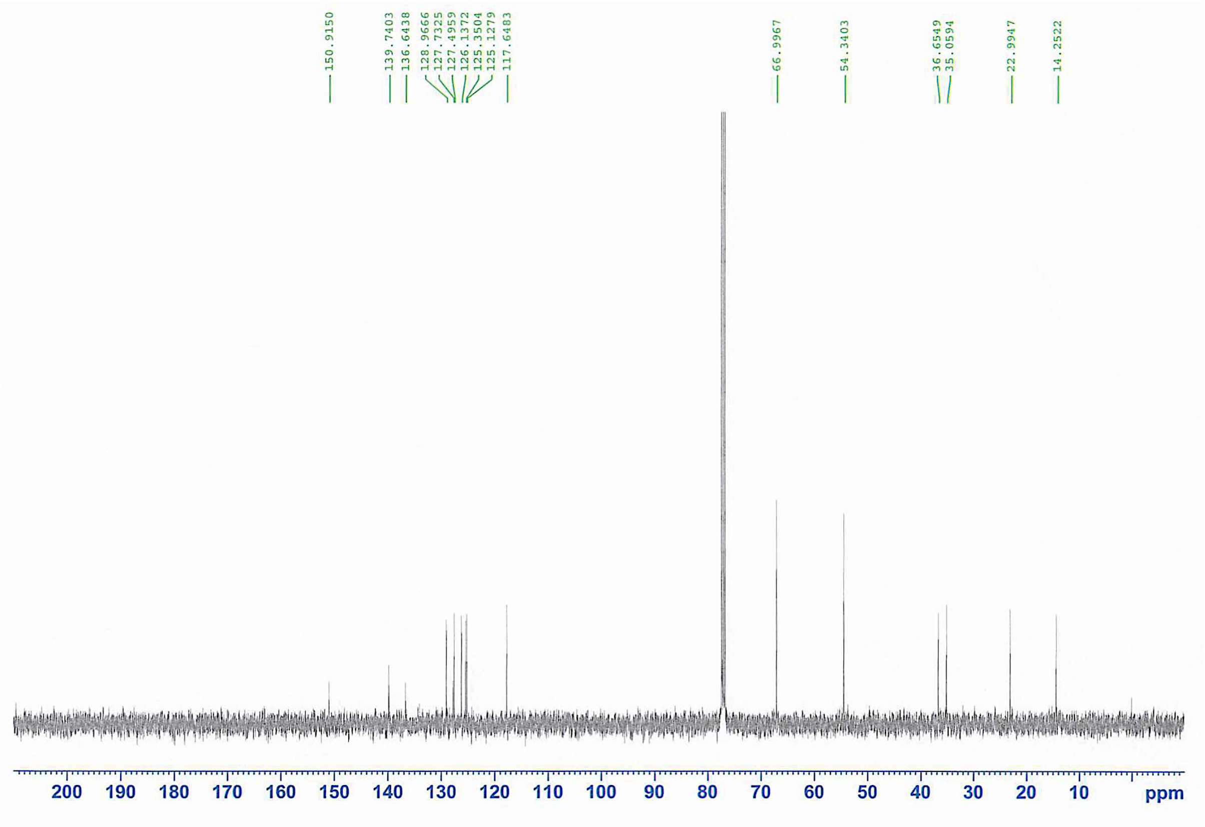

^{1}H NMR (400 MHz) and ^{13}C NMR (101 MHz) spectra of 4-(5-butylnaphthalen-1-yl)morpholine (**7j**) ($CDCl_3$)

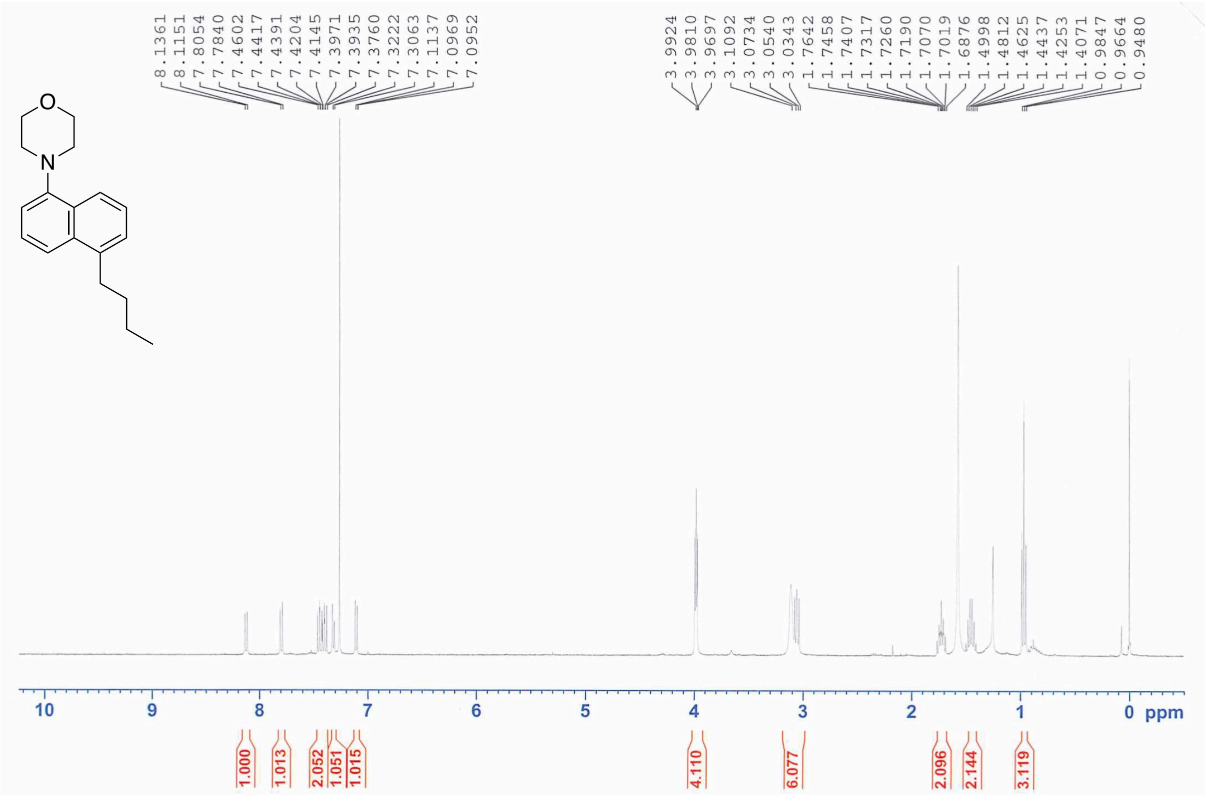

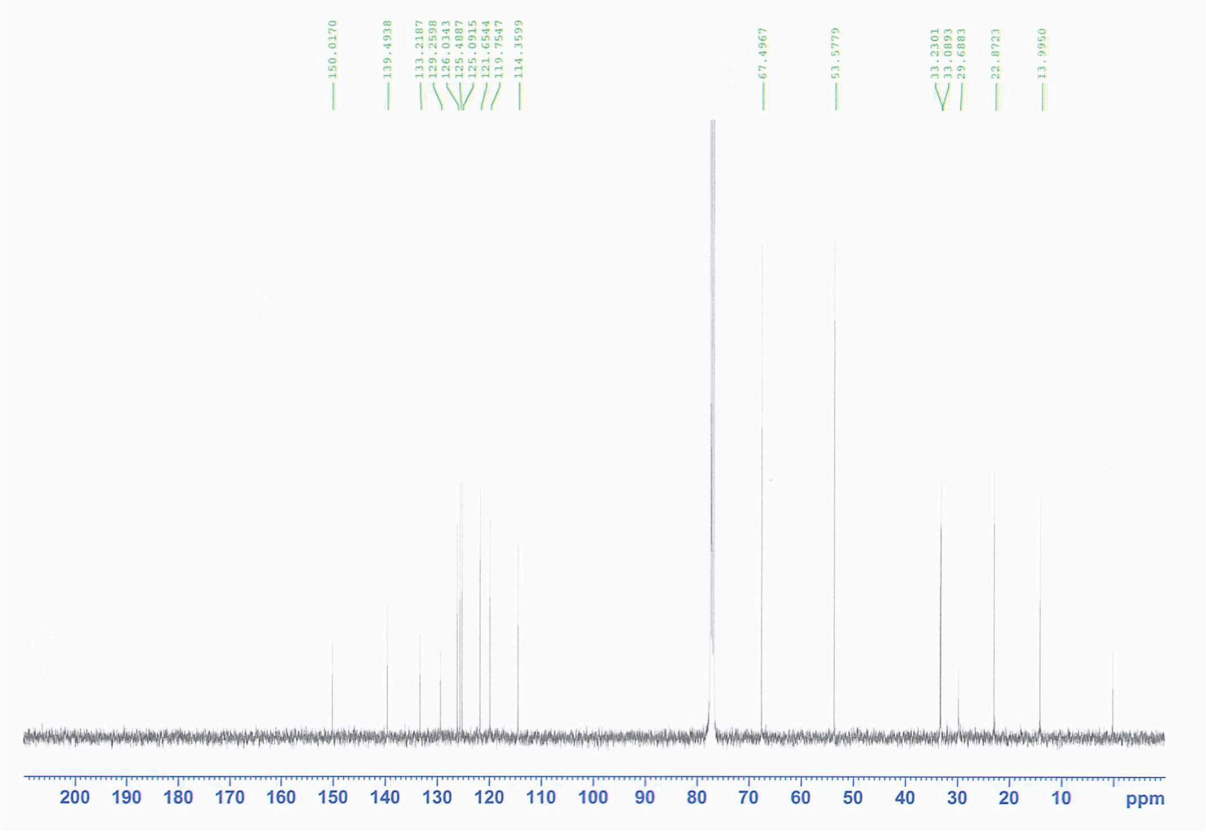

^{1}H NMR (400 MHz) and ^{13}C NMR (101 MHz) spectra of Methyl 6-methyl-8-morpholino-1-naphthoate (**7l**) ($CDCl_3$)

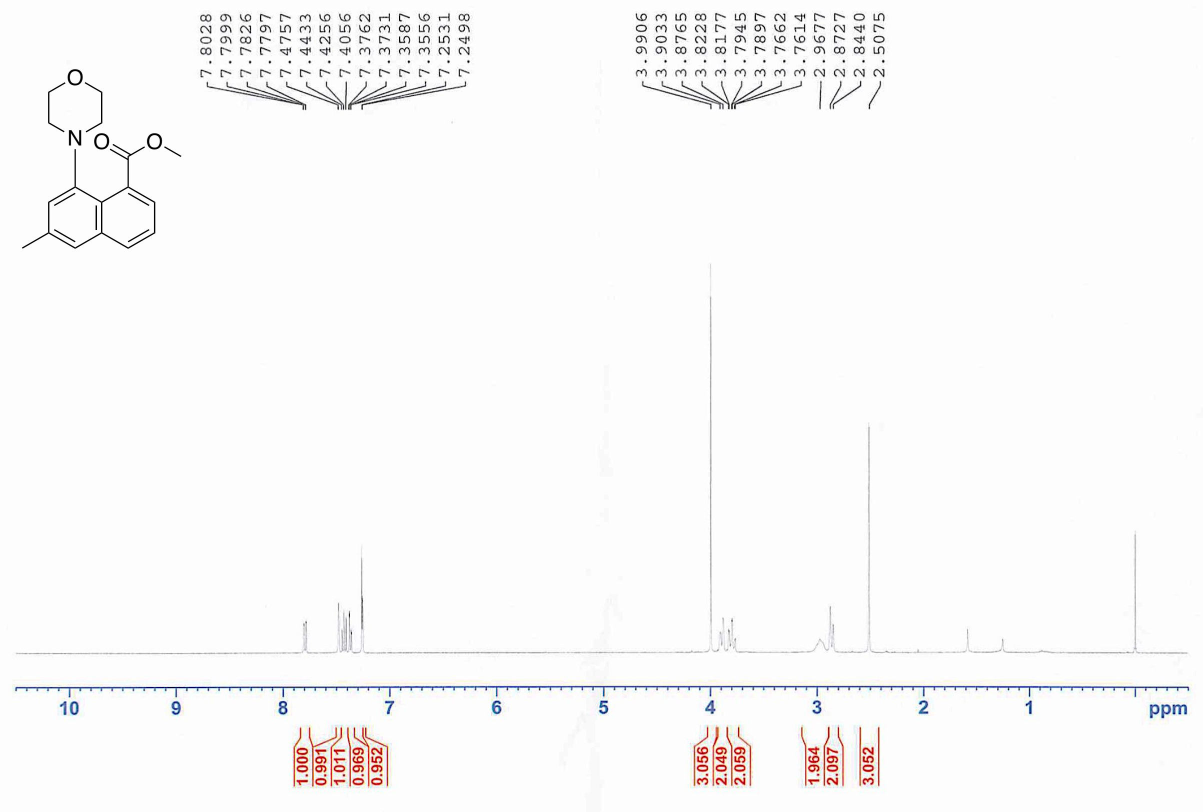

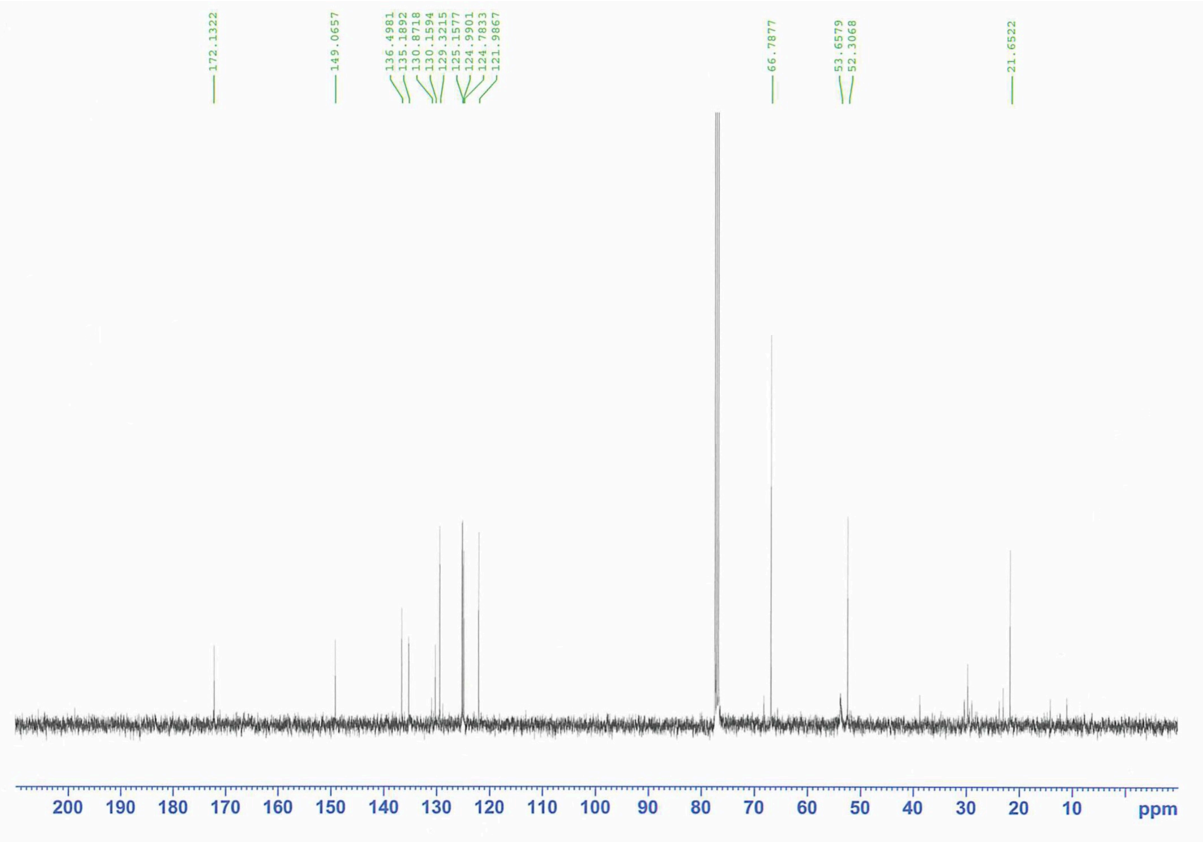

^{1}H NMR (400 MHz) and ^{13}C NMR (101 MHz) spectra of methyl 7-methyl-5-morpholino-1-naphthoate (**7m**) ($CDCl_3$)

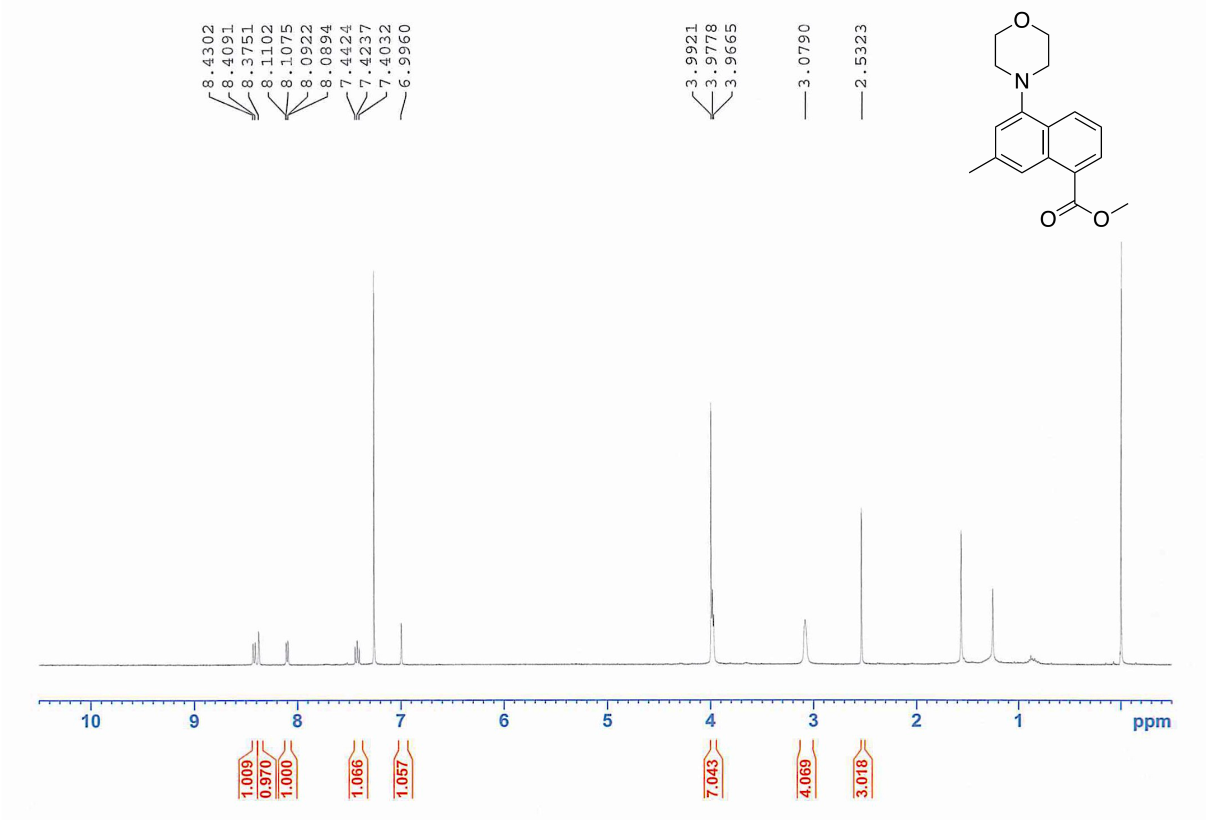

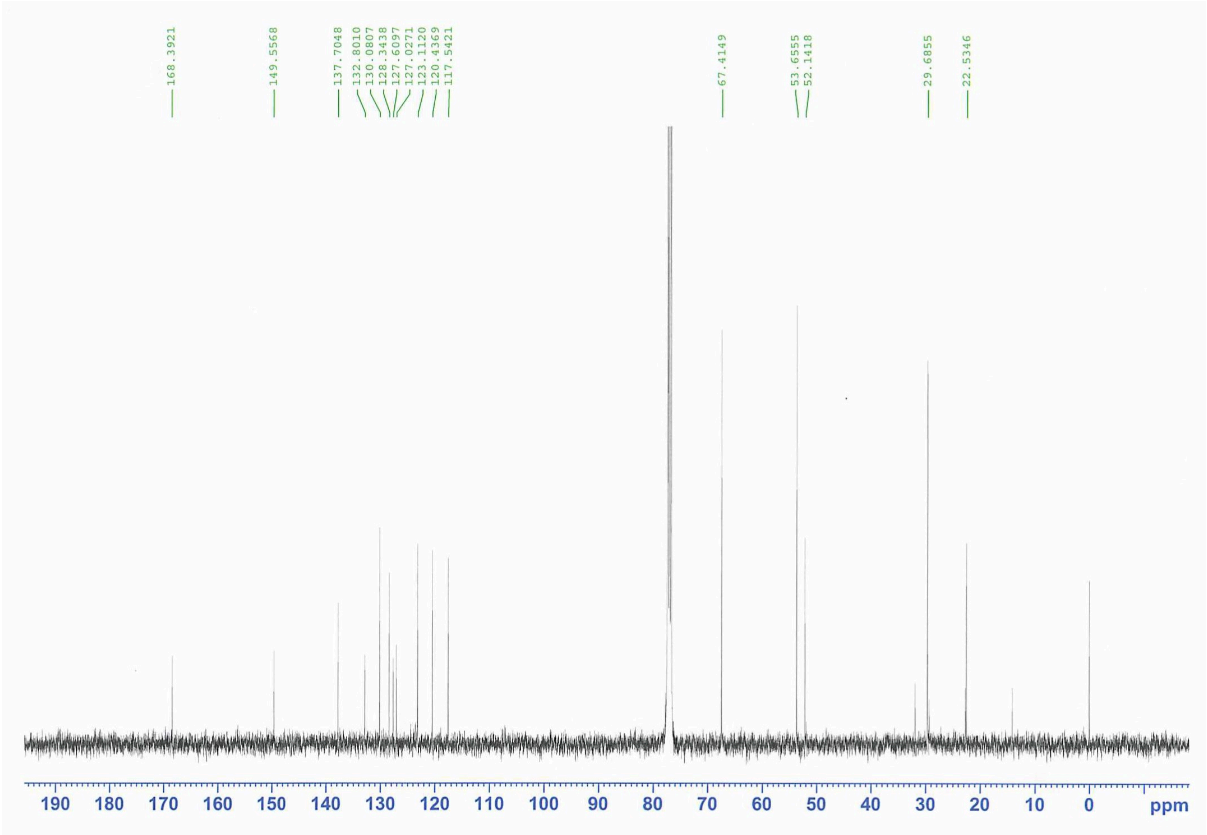

^{1}H NMR (400 MHz) and ^{13}C NMR (101 MHz) spectra of methyl 1-hydroxy-5-morpholino-2-naphthoate (**7n**) ($CDCl_3$)

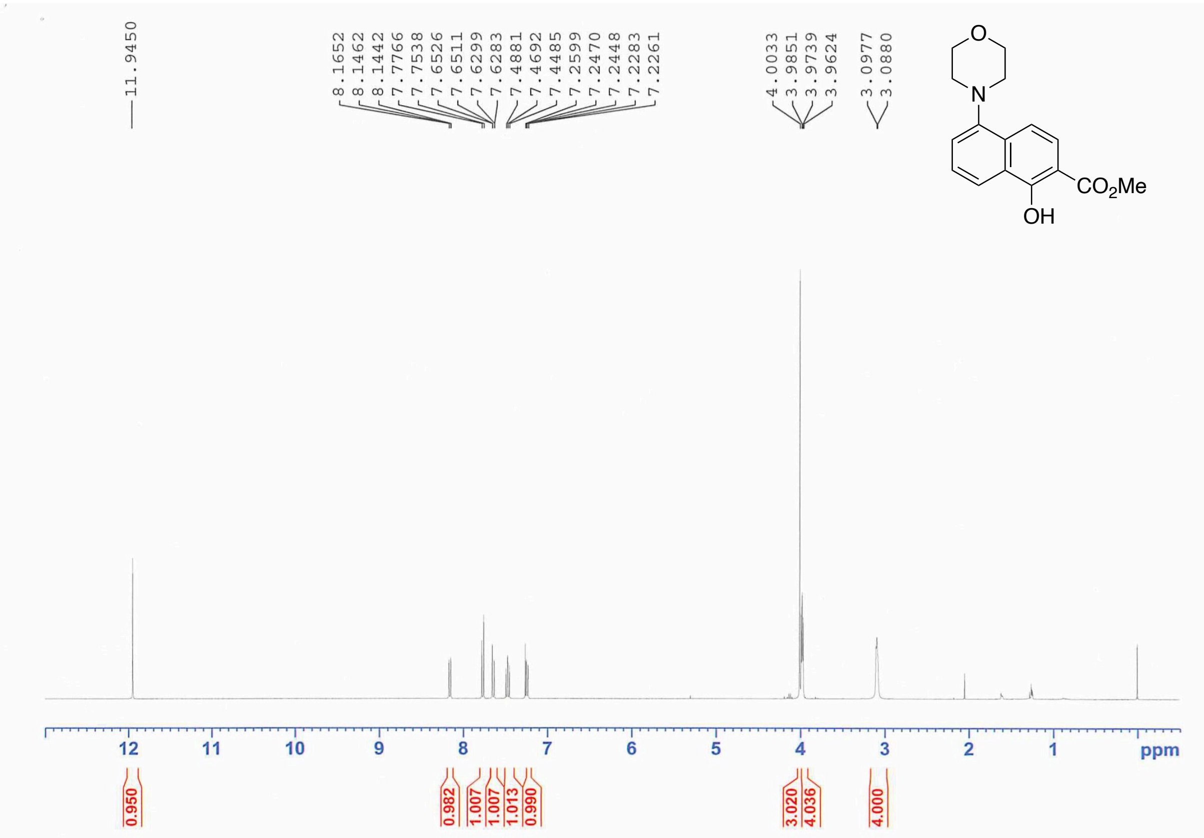

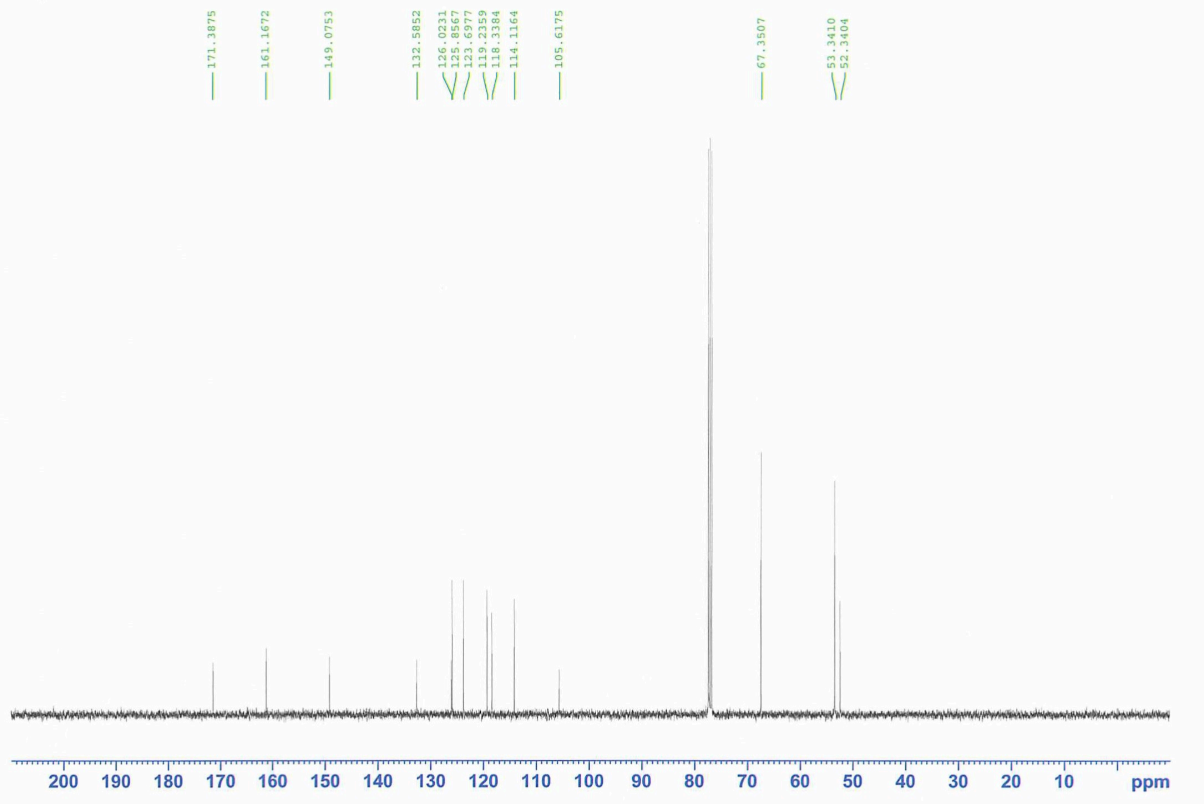

^{1}H NMR (400 MHz) and ^{13}C NMR (101 MHz) spectra of methyl 4-hydroxy-5-morpholino-1-naphthoate (**7o**) ($CDCl_3$)

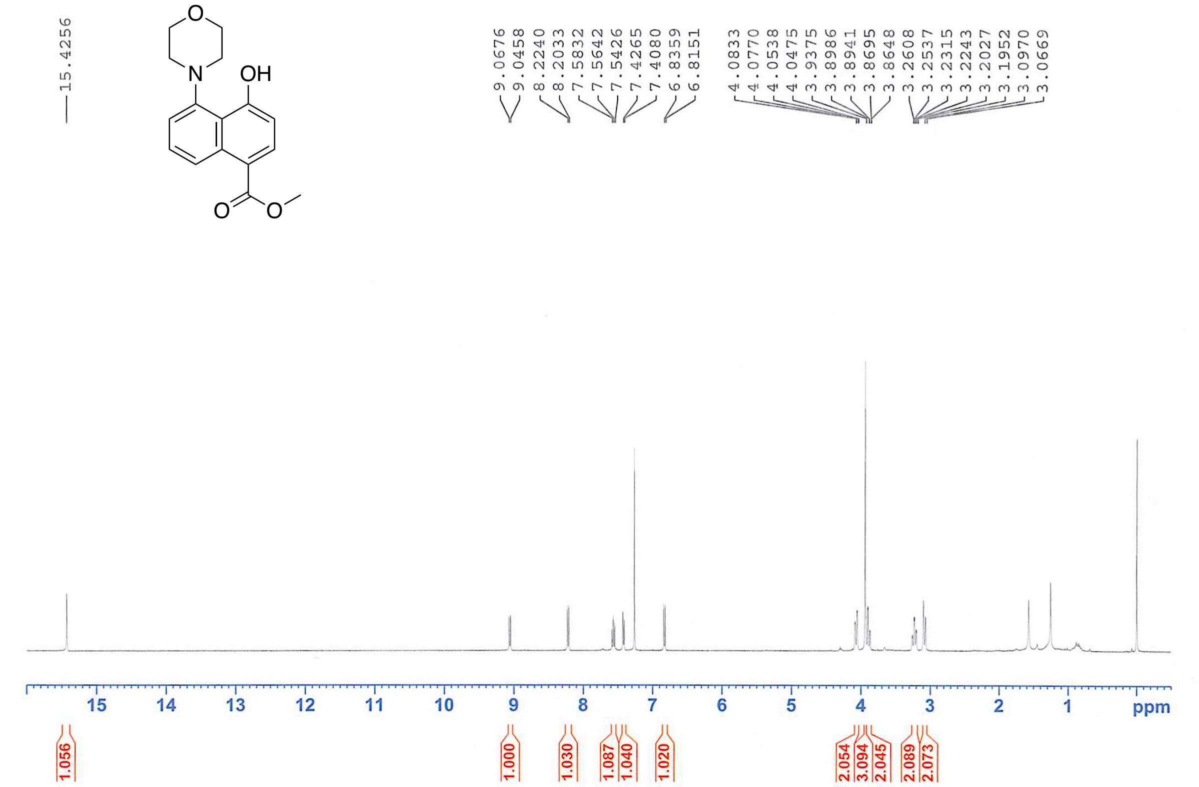

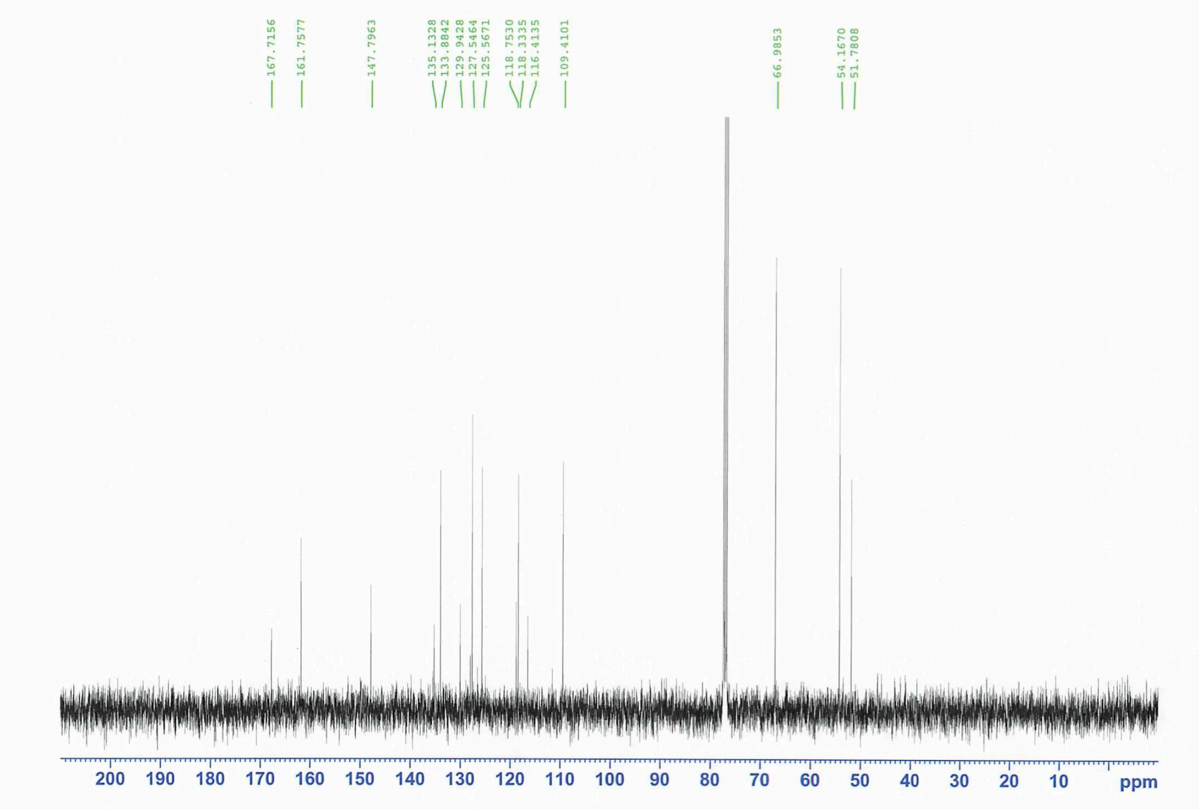

^{1}H NMR (400 MHz) and ^{13}C NMR (101 MHz) spectra of methyl 5-morpholino-3-(*p*-tolyl)-1-naphthoate (**7p**) ($CDCl_3$)

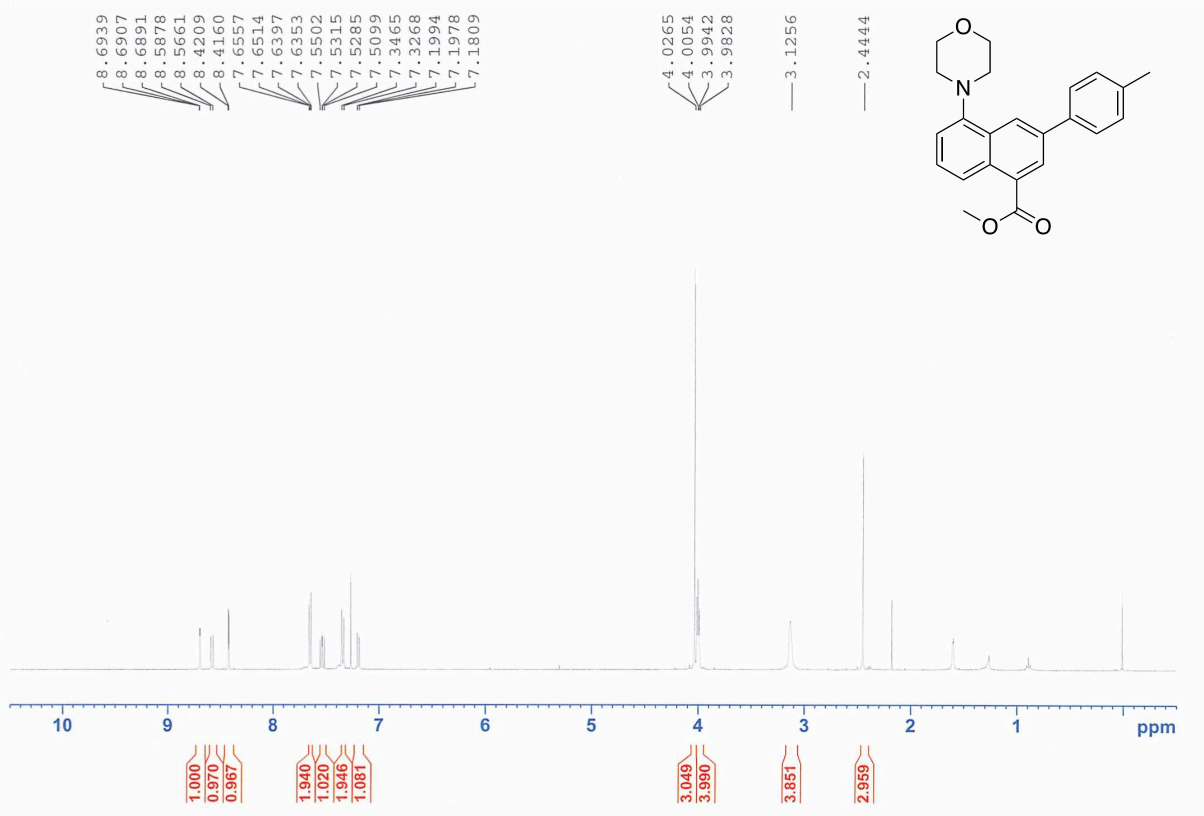

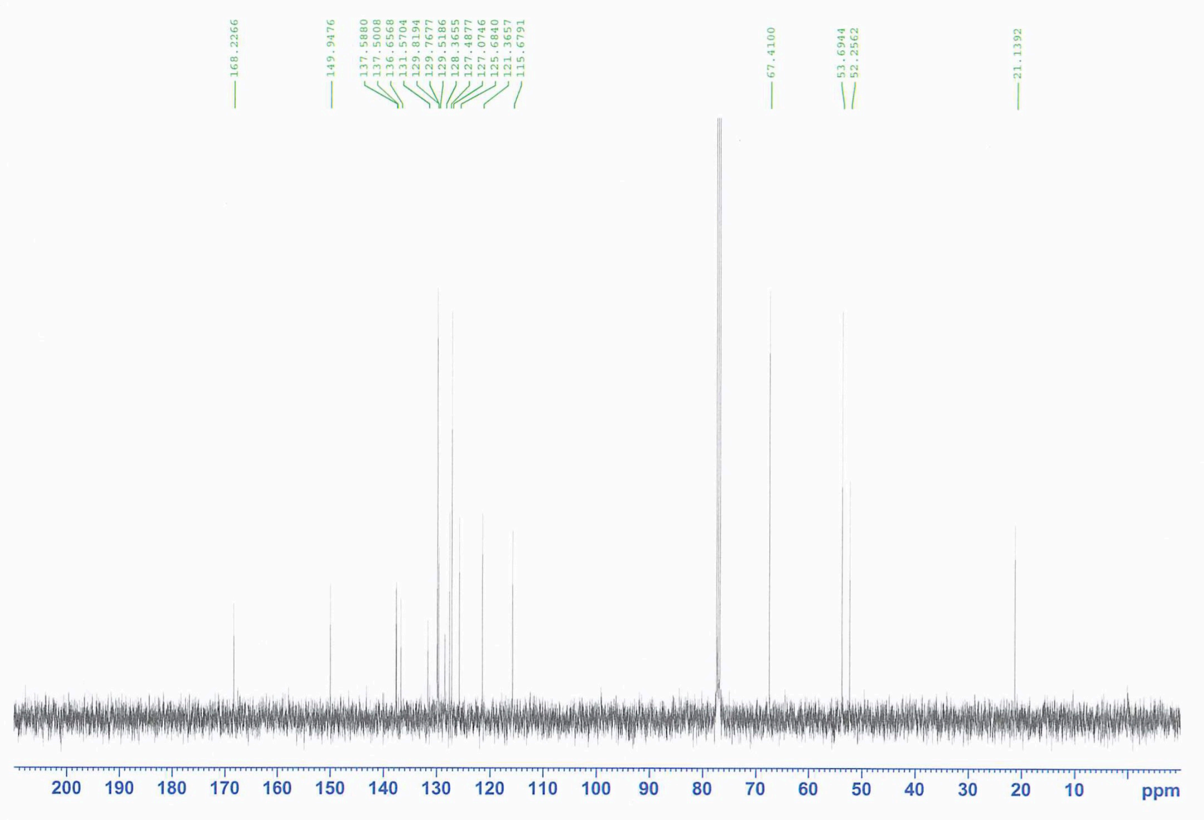

^{1}H NMR (400 MHz) and ^{13}C NMR (101 MHz) spectra of methyl 3-(4-((*tert*-butyldimethylsilyl)oxy)phenyl)-5-morpholino-1-naphthoate (**7q**) ($CDCl_3$)

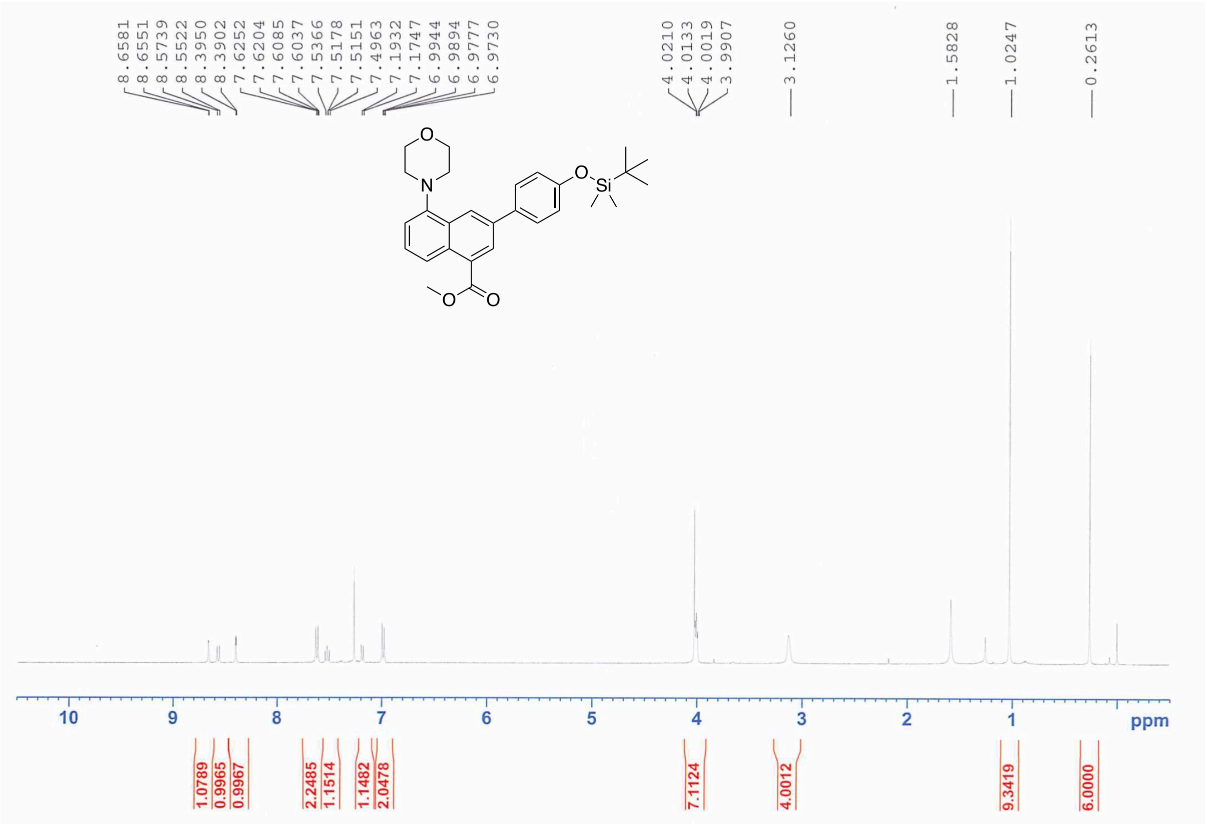

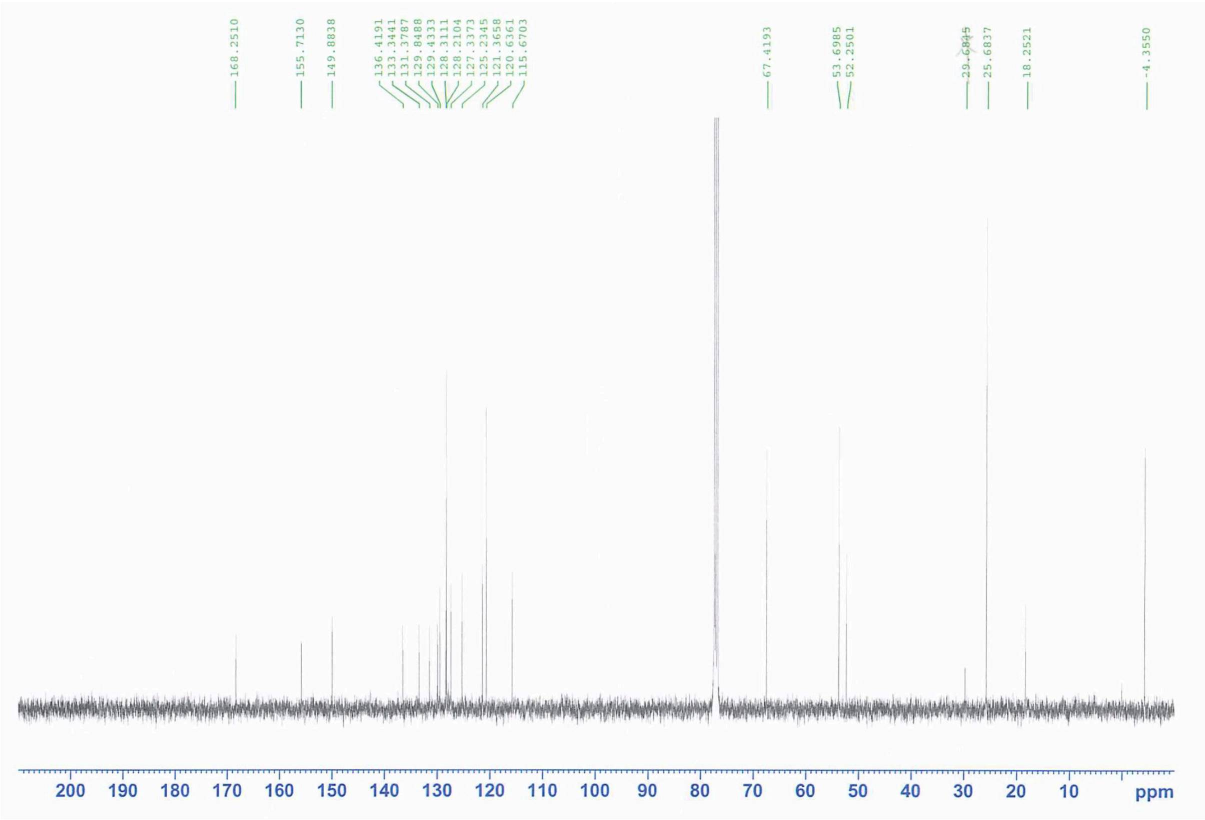

^{1}H NMR (400 MHz) and ^{13}C NMR (101 MHz) spectra of methyl 3-(4-((benzylsulfonyl)oxy)phenyl)-5-morpholino-1-naphthoate (**15**) ($CDCl_3$)

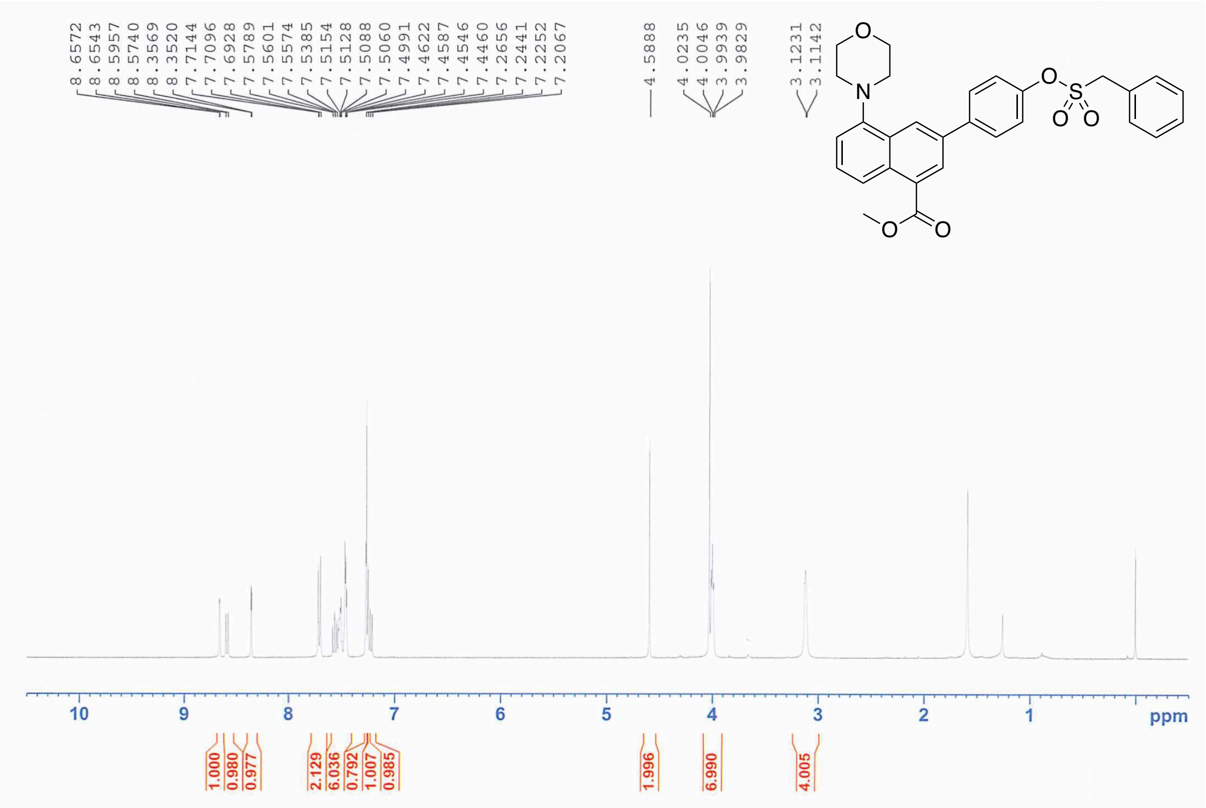

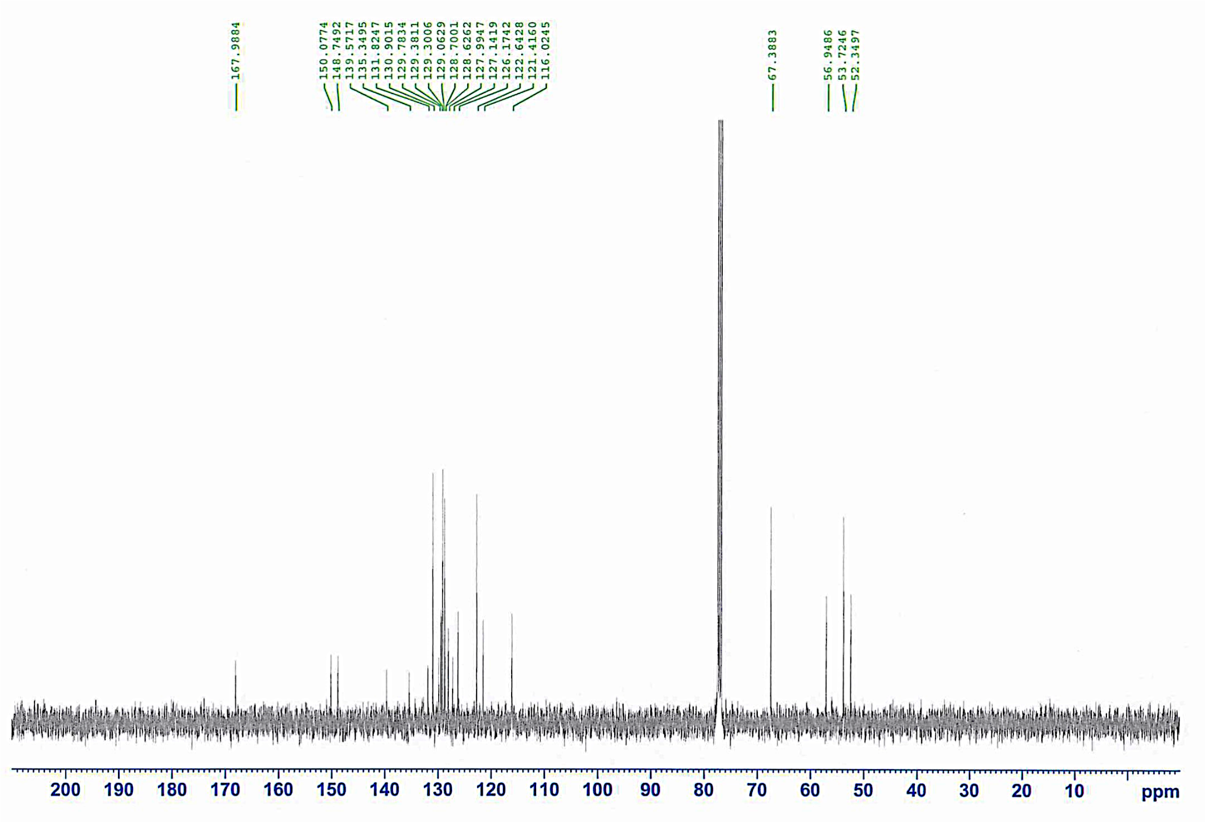

Supplement: Supplementary file 1 [file jo5c03079_si_001.pdf]
